# Engineering Morphology and Dimensionality in 1D and 2D Cu(I)‑I Coordination Polymers for Enhanced Photocatalysis, Luminescence, and Selective Antibiotic Sensing

**DOI:** 10.1021/acsomega.6c06025

**Published:** 2026-09-16

**Authors:** Julian Avila-Duran, Carlos Tabernero-Sestines, Fernando Aguilar-Galindo, Pilar Amo-Ochoa

**Affiliations:** † Inorganic Chemistry Department, Faculty of Sciences, Autonomous University of Madrid (UAM), 28049 Madrid, Spain; ‡ Chemistry Department, Faculty of Sciences, Autonomous University of Madrid (UAM), 28049 Madrid, Spain; § Institute for Advanced Research in Chemical Sciences (IAdChem), Autonomous University of Madrid (UAM), 28049 Madrid, Spain

## Abstract

Copper­(I) iodide coordination polymers (CPs) based on pyridine- and pyrazine-derived ligands are luminescent materials typically synthesized under solvothermal conditions using organic solvents. Herein, the archetypal compounds [CuI­(4-methylpyridine)]_n_ (**CP1**) and [Cu_2_I_2_(2-methylpyrazine)]_n_ (**CP2**) were synthesized through one-step room-temperature methods, reducing the need for conventional high-temperature solvothermal procedures. Particle size modulation using polyvinylpyrrolidone and postsynthetic treatments, by sonication or grinding, enabled control over the morphology while preserving the crystal structure. Sonication additionally produced stable aqueous suspensions with enhanced dispersibility and processability. Both CPs exhibited size-dependent optical properties, with band gaps ranging from 2.1 to 3.2 eV for **CP1** and from 2.2 to 2.4 eV for **CP2**. Reduced particle size and increased effective surface area led to enhanced photocatalytic activity toward methyl orange degradation, achieving complete degradation within 6 and 3 min, respectively, for the smallest particles. **CP1** and **CP2** displayed intense luminescence centered at 440 and 700 nm, respectively, and acted as luminescent sensors for tetracycline in water, with limits of detection (LODs) of 410 and 2800 nM, respectively. Furthermore, the improved processability of the sonicated materials enabled their incorporation into 3D-printed photopolymer composites and PLA films. These results highlight the potential of sustainable Cu­(I)-iodide CPs for environmental remediation, antibiotic sensing, and portable functional devices.

## Graphical abstract

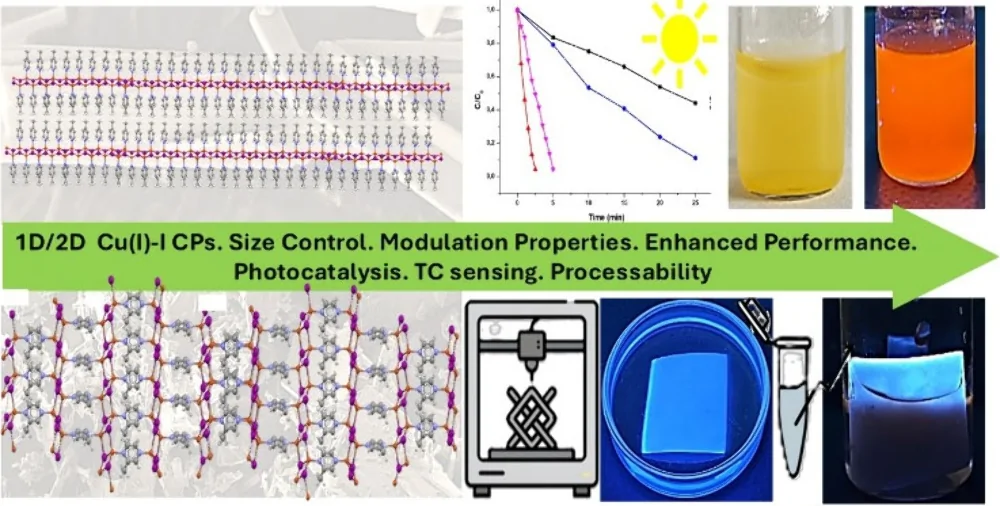

## Introduction

Coordination polymers (CPs) represent a broad class of extended solids constructed from metal centers linked by organic ligands, offering virtually limitless structural diversity. The systematic investigation of CPs did not emerge until the mid-20th century, culminating in foundational reviews in the 1960s.^1^ A defining characteristic of many CPs is their low solubility, particularly in water, which has historically limited their processing but simultaneously enabled their use as robust functional materials.^2^ Among these systems, Cu­(I)-iodide CPs incorporating pyridine- or pyrazine-derived ligands have attracted sustained attention due to their rich structural chemistry and versatile photophysical behavior.^3,4^

From the 1970s through the end of the 20th century, research on these materials focused primarily on elucidating their optoelectronic properties and understanding structure–property relationships at the crystallographic level.^5^ Numerous Cu­(I)-I CPs were shown to exhibit luminescence, stimuli-responsive behavior, and sensitivity to external perturbations such as gases, temperature, or pressure.^6–8^ More recently, attention has shifted toward controlling crystal size and morphology as an additional parameter for tuning these properties, recognizing that reduced dimensions can markedly influence optical response, surface reactivity, and material processability.^9–11^ This evolution parallels broader trends in materials chemistry that emphasize scalable fabrication and integration into functional devices.^12,13^

At the same time, increasing emphasis on sustainable synthesis has motivated the replacement of traditional solvothermal routes, often requiring elevated temperatures, pressures, long reaction times, and organic solvents, with milder, environmentally benign methodologies. For Cu­(I)-I CPs, this shift enables preparation under ambient conditions, using milder solvent systems, and through single-step processes, significantly reducing energy input and chemical waste.^4^ Such strategies also provide an opportunity to revisit classical compounds reported decades ago and reassess their properties in forms better suited for modern applications.

[CuI­(4-methylpyridine)]_n_ (**CP1**) known for its responsiveness to gaseous environments, was selected as a representative Cu­(I)-iodide CP prepared by a previously reported room-temperature aqueous route that allows particle-size control.^6^ Another representative example is [Cu_2_I_2_(2-methylpyrazine)]_n_ (**CP2**), originally obtained as crystalline material via solvothermal synthesis in acetonitrile at 130 °C over several days.^14^ Size reduction increases the effective surface area and can enhance performance in heterogeneous photocatalysis, as previously demonstrated for related systems.^15^ Moreover, modulation of particle dimensions enables stable aqueous suspensions, facilitating processing into functional forms while preserving the intrinsic water insolubility of the coordination framework.

In this context, controlling size and morphology provides a powerful means to tailor the optical band gap, luminescence, and interfacial reactivity of Cu­(I)-I CPs, thereby expanding their applicability in environmental remediation, chemical sensing, and device fabrication. The present work explores how milder synthetic access combined with postsynthetic size modulation can revalorize these archetypal materials, linking dimensional control to photocatalytic activity, analyte detection in water, and improved processability into composite platforms.

## Experimental Details

### Materials

All reagents were used as obtained from the suppliers. Acetonitrile (≥99.93%), ethanol (≥99.9%), and isopropanol (≥99.8%) were purchased from Scharlau. Copper­(I) iodide (≥99%) and 2-methylpyrazine (≥99%) were obtained from Sigma-Aldrich. Potassium iodide (≥99.5%) was purchased from Fluka. 4-Methylpyridine (≥99%) was supplied by Thermo Scientific. Dyes and the antibiotics used in this work: methyl orange (MO, >85%), methylene blue (MB, >82%), rhodamine B (Rh, ≥95%), sulfamethazine (SMZ, >99.0%), chloramphenicol (CAP, >98%), and tetracycline (TC, >98%), all purchased at Sigma-Aldrich. *tert*-Butanol (99.5%) was supplied by Acros. Copper­(II) sulfate (≥99%), sodium azide (>99.5%), acrylamide (≥99%), and ammonium oxalate (98%) were obtained from Sigma-Aldrich. Polyvinylpyrrolidone K90 (PVP) and polylactic acid-biopolymer compounds (PLA) and granules (3 mm in nominal granule size) were obtained from Sigma-Aldrich. Standard Photopolymer Translucid Resin was purchased from ELEGOO (Lewes, DE, USA).

### Synthesis of [CuI­(4-Methylpyridine)]_n_ (**CP1**) and [Cu_2_I_2_(2-Methylpyrazine)]_n_ (**CP2**)

#### **CP1** at Room Temperature (**CP1-RT**)

CuI (190.5 mg, 1 mmol) was dissolved into a saturated aqueous KI solution (4 mL) under magnetic stirring (800 rpm) at room temperature. After that, 4-methylpyridine (93.1 mg, 1 mmol) was added over that solution. Immediately a white precipitate was formed, and the reaction is stirred at 25 °C for 15 min. Then, the obtained precipitate was filtered off under vacuum and washed with concentrated aqueous KI (10 mL × 2) and H_2_O (15 mL × 3) respectively. Finally, the precipitate was dried under vacuum for 24 h. Yield: 75% (213 mg).

Elemental analysis (%) calculated for [CuI­(C_6_H_7_N)]_n_: C, 25.41; H, 2.49; N, 4.94. Experimental: C, 25.46; H, 2.57; N, 4.94. IR (cm^–1^): 1680 (w), 1611 (m), 1557 (w), 1495 (m), 1420 (m), 1382 (w), 1329 (w), 1227 (w), 1216 (w), 1206 (w), 1067 (w), 1013 (m), 805 (s), 718 (m) and 672 (w). PXRD and IR (Figures S1 and S2).

#### **CP2** at Room Temperature (**CP2-RT**)

CuI (400 mg, 2.05 mmol) was dissolved into CH_3_CN (15 mL) under magnetic stirring (800 rpm) at 25 °C. After that, 2-methylpyrazine (70 mg, 0.74 mmol) was added. Immediately, an orange precipitate was formed, and the reaction was stirred at 25 °C for 15 min. The obtained precipitate was filtered off under vacuum and washed with CH_3_CN (2 mL × 4) and ethanol (2 mL × 4) respectively. Finally, the precipitate was dried under vacuum for 24 h. Yield: 93% (442.2 mg).

Elemental analysis (%) calculated for [Cu_2_I_2_(C_5_H_6_N_2_)]_n_: C, 12.64; H, 1.27; N, 5.9. Experimental: C, 12.75; H, 1.53; N, 5.93. IR (cm^–1^): 3084 (w), 3019 (w), 1594 (w), 1511 (w), 1472 (m), 1460 (w), 1442 (s), 1385 (w), 1294 (m), 1173 (w), 1150 (s), 1074 (m), 1036 (m), 987 (w), 968 (w), 919 (w), 831 (s), 737 (w) and 665 (w). PXRD and IR (Figures S1 and S2).

#### **CP2** Solvothermal Conditions (**CP2-ST**)

Solutions of CuI (405 mg, 2.12 mmol) in CH_3_CN (15 mL) are prepared under magnetic stirring (800 rpm) at room temperature. After that, ligand of 2-methylpyrazine (140 mg, 1.48 mmol) is added. This solution is heated at 130 °C for 40h and allowed to cool slowly to room temperature for 80h. The yellow precipitate is filtered off under vacuum and washed with CH_3_CN (2 mL × 4) and ethanol (2 mL × 4) respectively. Finally, the precipitate is dried under vacuum for 24 h. Yield: 83% (393.3 mg).

Elemental analysis (%) calculated for [Cu_2_I_2_(C_5_H_6_N_2_)]_n_: C, 12.64; H, 1.27; N, 5.9. Experimental: C, 12.72; H, 1.42; N, 5.89. IR (cm^–1^): 3057 (w), 2989 (w), 2921 (w), 1666 (w), 1567 (w), 1484 (w), 1446 (m), 1431 (w), 1416 (s), 1355 (m), 1264 (m), 1150 (w), 1124 (s), 1055 (s), 1006 (s), 964 (w), 942 (w), 888 (w), 962 (w), 805 (s), 782 (w), 710 (w), 673 (w) and 638 (w). PXRD and IR (Figures S1 and S2).

### Size Reduction of [CuI­(4-Methylpyridine)]_n_ (**CP1**) and [Cu_2_I_2_(2-Methylpyrazine)]_n_ (**CP2**): Bottom-Up Approach

#### Use of Polyvinylpyrrolidone (PVP)

##### CP1-PVP

PVP K90 (25 mmol, 2.800 g) was dissolved in 40 mL of water (7% w/v PVP). Two mL of a saturated aqueous KI solution was prepared and used to dissolve CuI (0.5 mmol, 0.095 g). The CuI/KI solution was added dropwise to the PVP solution, which was left to stir for 2 h (800 rpm) at room temperature. The 4-methylpyridine (2 mmol, 0.186 g) was added dropwise to the flask containing CuI/KI/PVP. The contents of the flask become instantly cloudy, showing a white colored suspension. The suspension formed is washed with ethanol (10 mL × 2) and water (10 mL × 2) by centrifugation at 4600 rpm for 10 min. Finally, the precipitate was dried under vacuum for 24 h. Yield: 84% (120 mg).

Elemental analysis (%) calculated for [CuI­(C_6_H_7_N)]_n_: C, 25.41; H, 2.49; N, 4.94. Experimental: C, 26.30; H, 2.70; N, 4.95. IR (cm^–1^): 1680 (w), 1614 (m), 1555 (w), 1498 (m), 1422 (m), 1383 (w), 1329 (w), 1227 (w), 1216 (w), 1206 (w), 1071 (w), 1016 (m), 805 (s), 721 (m) and 700 (w). PXRD and IR (Figures S1 and S2).

##### CP2-PVP

PVP K90 (25 mmol, 2.800 g) was dissolved in 40 mL of water (7% w/v PVP). On the other hand, CuI (0.5 mmol, 0.095 g) was dissolved into 7 mL of CH_3_CN, under magnetic stirring (800 rpm) at 25 °C. The CuI solution was added dropwise to the PVP solution, which was left to stir for 2 h (800 rpm) at 25 °C. The 2-methylpyrazine (2 mmol, 0.035 g) was then added dropwise to the flask containing CuI/PVP. The contents of the flask become instantly cloudy, showing an orange-colored suspension. The suspension formed is washed with ethanol (10 mL × 2) and water (10 mL × 2) by centrifugation at 4600 rpm for 10 min. Finally, the precipitate is dried under vacuum for 24 h. Yield: 55% (65.4 mg).

Elemental analysis (%) calculated for [Cu_2_I_2_(C_5_H_6_N_2_)]_n_: C, 12.64; H, 1.27; N, 5.9. Experimental: C, 13.81; H, 1.54; N, 5.95. IR (cm^–1^): 3057 (w), 2989 (w), 2890 (w), 1647 (w), 1567 (w), 1484 (m), 1446 (s), 1431 (w), 1412 (s), 1363 (m), 1265 (m), 1150 (w), 1127 (s), 1048 (s), 1006 (m), 960 (w), 942 (w), 888 (w), 805 (s), 714 (w) and 634 (w). PXRD and IR (Figures S1 and S2).

### Size Reduction of [CuI­(4-Methylpyridine)]_n_ (**CP1**) and [Cu_2_I_2_(2-Methylpyrazine)]_n_ (**CP2**): Top-Down Approach

#### Ultrasound (US): **CP1-US** and **CP2-US**

Dispersions of 10 mg of **CP1-RT** and **CP2-RT** respectively, in Milli-Q water at 25 °C were prepared at a ratio of 1 mg/1 mL. These were placed in an 80% ultrasonic bath for 1 h. The remaining solutions were centrifuged before separating the supernatant. The solids were allowed to dry for 24 h under vacuum.

**CP1-US**: IR (cm^–1^):1679 (w), 1612 (m), 1555 (w), 1497 (m), 1421 (m), 1383 (w), 1329 (w), 1227 (w), 1215 (w), 1206 (w), 1071 (w), 1016 (m), 805 (s), 721 (m) and 700 (w). PXRD and IR (Figures S1 and S2).

**CP2-US**: IR (cm^–1^): 3057 (w), 2989 (w), 2921 (w), 1666 (w), 1567 (w), 1484 (w), 1446 (m), 1431 (w), 1416 (s), 1355 (m), 1264 (m), 1150 (w), 1124 (s), 1055 (s), 1006 (s), 964 (w), 942 (w), 888 (w), 962 (w), 805 (s), 782 (w), 710 (w), 673 (w) and 638 (w). PXRD and IR (Figures S1 and S2).

#### Grinding (GR): **CP1-GR** and **CP2-GR**

20 mg of each **CP-RT** was ground for 5 min in an agate mortar until homogeneous mixtures were obtained.

**CP1-GR**: IR (cm^–1^): 1678 (w), 1612 (m), 1555 (w), 1497 (m), 1421 (m), 1383 (w), 1329 (w), 1226 (w), 1215 (w), 1206 (w), 1070 (w), 1016 (m), 805 (s), 721 (m) and 700 (w). PXRD and IR (Figures S1 and S2).

**CP2-GR**: IR (cm^–1^): 3076 (w), 3008 (w), 2910 (w), 1586 (w), 1499 (w), 1469 (m), 1449 (m), 1431 (s), 1400 (w), 1374 (m), 1283 (m), 1166 (w), 1143 (s), 1066 (s), 1025 (m), 976 (w), 960 (w), 907 (w), 824 (s), 729 (w) and 654 (w). PXRD and IR (Figures S1 and S2).

### Processed with Polylactic Acid (PLA) Composite Sheets: **CP1@DC** and **CP2@DC**

#### Method 1

0.08 g of PLA was placed in a vial and dissolved in 2 mL of 1,4-dioxane under magnetic stirring at 800 rpm for 24 h. Then, 0.8 mg (1% wt/wt) of **CP1-US** and **CP2-US** particles was introduced, and a uniform mixture was obtained after 10 min of stirring and 1 min of sonication in an ultrasonic bath at 24 °C at 80% power. A 1 mL aliquot of this suspension was poured dropwise onto 2.5 × 2.5 cm^2^ glass substrates, which were kept at room temperature for 24 h to evaporate the solvent.

#### Method 2

0.08 g of PLA was placed in a vial and dissolved in 2 mL of acetonitrile at 60 °C under magnetic stirring at 800 rpm for 3 h. Then, 2.4 mg (3% wt/wt) of **CP1-US** and **CP2-US** particles was introduced, and a uniform mixture was obtained after 5 min of stirring and 1 min of sonication in an ultrasonic bath at 24 °C at 80% power. A 1 mL aliquot of this suspension was poured dropwise onto 2.5 × 2.5 cm^2^ glass substrates, which were kept at room temperature for 24 h to evaporate the solvent.

### Processed with Commercial Resin for 3D Printing: **CP1@3D** and **CP2@3D**

A 2.5 × 2.5 cm^2^ film, 0.5 mm thick and loaded with 1% of sonicated **CP1** or **CP2** particles, was produced from a standard commercial 3D printing resin. Specifically, 3 mL of resin were combined with 0.5 mL of an Milli-Q water suspension containing **CP1-US** or **CP2-US** at 66 mg/mL. This mixture was then sonicated in an ultrasonic bath at 80% power for 1 min. Following sonication, the resulting dispersion was introduced into the 3D printing tank to form the final film.

### Methods and Equipment

The methods and equipment used for characterization, property study, and processing of the compounds, such as infrared, elemental analysis, powder X-ray diffraction, diffuse reflectance, scanning electron microscopy, atomic force microscopy, emission measurements, photocatalysis, proton magnetic resonance, sonication, 3D printing, and theoretical calculations, are described in detail in the Supporting Information.

## Results and Discussion

### Modification of Synthetic Conditions to Reduce Particle Size

Scheme 1 illustrates the various synthetic routes and reaction conditions employed to obtain the CPs, starting from CuI and 4-methylpyridine (**CP1**) and 2-methylpyrazine (**CP2**), respectively.

**Scheme 1:**
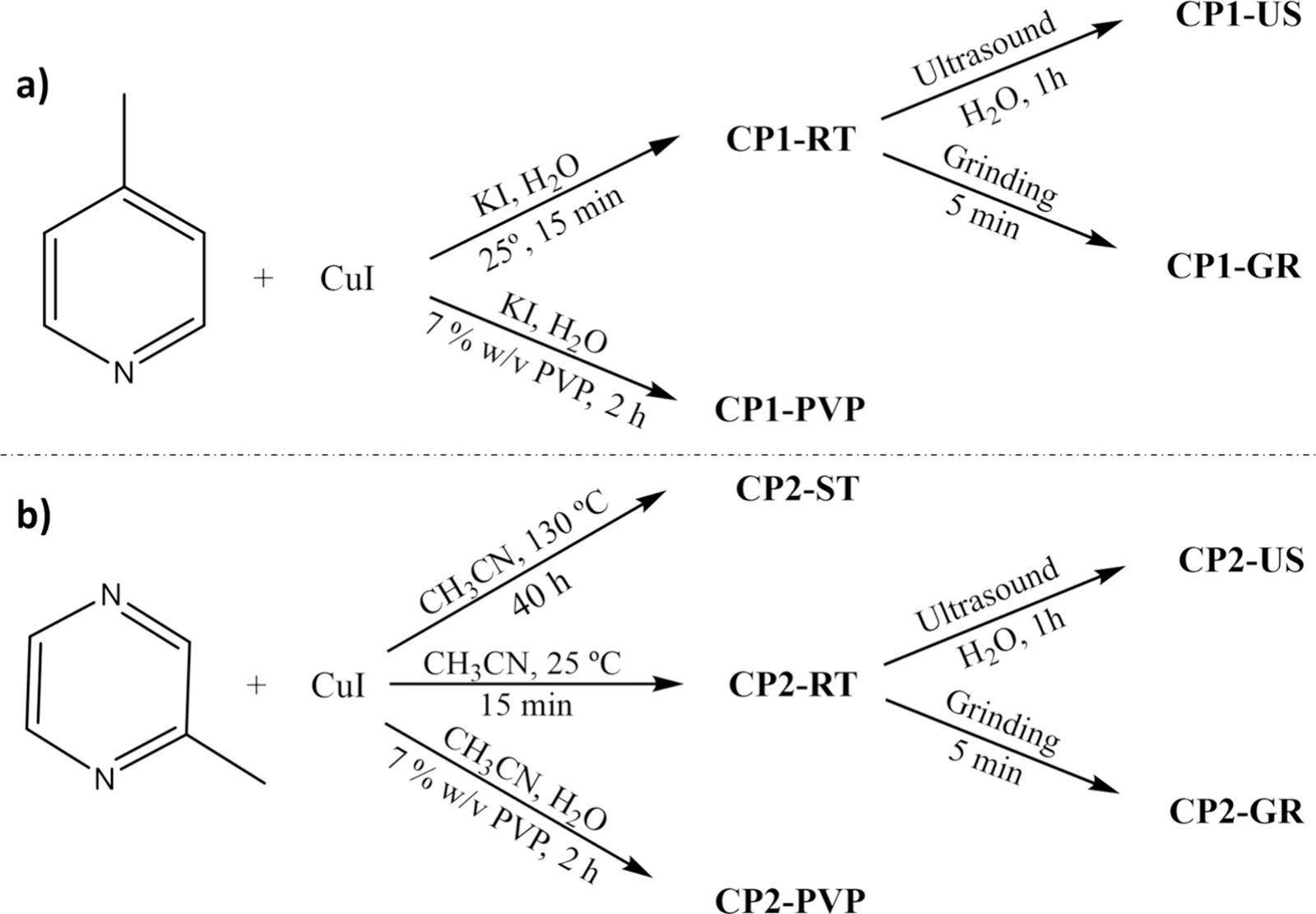
Synthetic Routes and Experimental Conditions Used for the Preparation of **CP1** and **CP2**

**CP1** ([CuI­(4-methylpyridine)]_n_) and **CP2** ([Cu_2_I_2_(2-methylpyrazine)]_n_),^6,14^ are built from Cu­(I)-iodide secondary building units but differ in their structural dimensionality: **CP1** adopts a one-dimensional (1D) chain-like arrangement, whereas **CP2** forms a two-dimensional (2D) layered architecture (Figure 1). Despite this distinction, both materials share electronically coupled Cu­(I)–I cores and π-conjugated N-donor ligands, enabling efficient light absorption and charge-transfer processes.^16,17^ These characteristics have made Cu­(I)-iodide CPs attractive platforms for optoelectronic and photocatalytic applications.^18^ Dimensionality is expected to influence their electronic structure and surface accessibility, as 2D frameworks typically provide more exposed active sites and improved carrier mobility compared to 1D analogues.^19^

**Figure 1:**
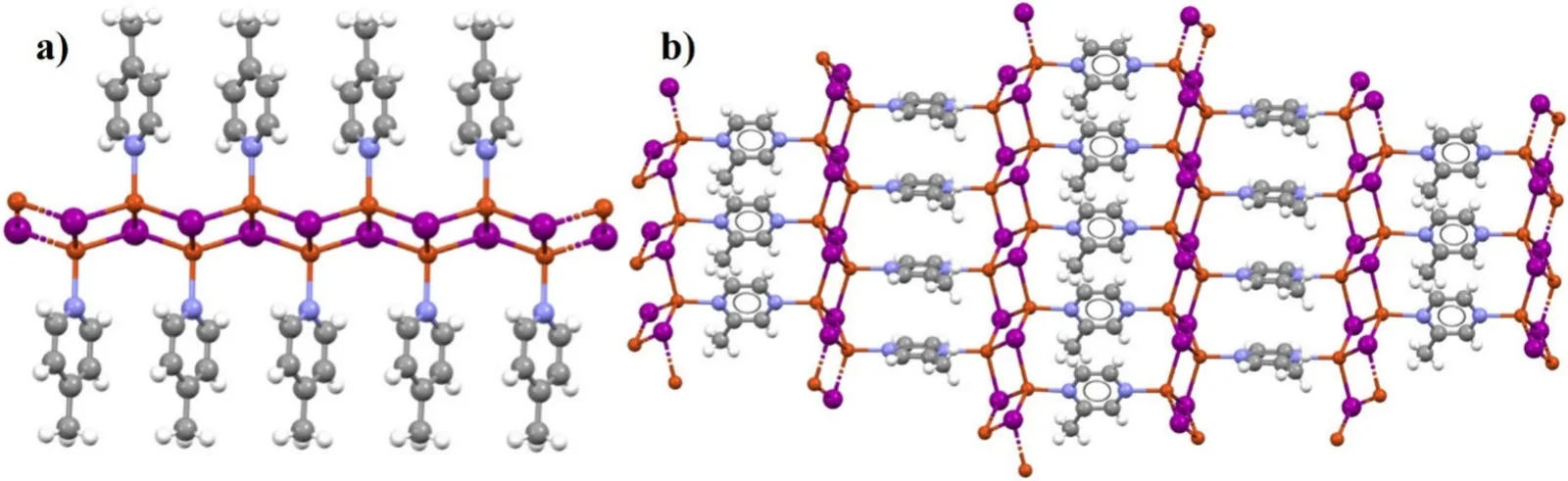
Fragment of the single-crystal structures of (a) 1D **CP1** and (b) 2D **CP2**, generated using Mercury from previously reported crystallographic information deposited in the crystallographic database.^6,14^ The different colors of the spheres indicate the atom type: orange, copper; violet, iodine; blue, nitrogen; gray, carbon; and white, hydrogen.

Under conventional solvothermal synthesis (**CP2-ST**), **CP2** crystallizes via slow nucleation and prolonged crystal growth to yield large single crystals (tens of micrometers, 32 μm length) (Figure 2). While suitable for structural characterization, such large particles exhibit limited surface area and poor dispersibility. Reducing particle size is therefore expected to enhance photocatalytic behavior by increasing the surface-to-volume ratio and strengthening light–matter interactions.^20,21^ Furthermore, nanometric dimensions can improve charge separation and reduce recombination rates by shortening the diffusion path of photogenerated carriers. In semiconducting CPs, particle size and band gap should be regarded that can influence and potentially optimize the photocatalytic activity.^20,22^

**Figure 2:**
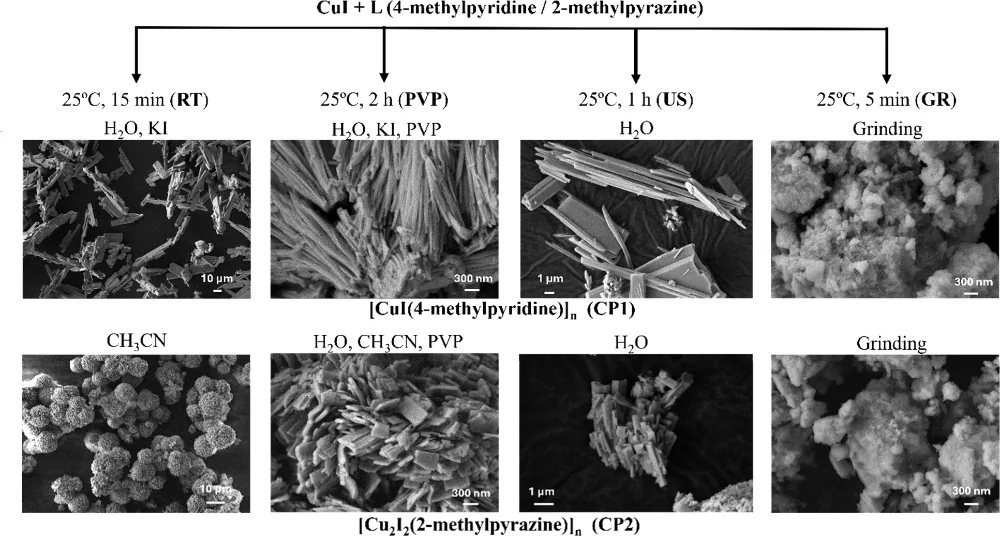
Summary diagram of the different particles obtained for **CP1** and **CP2**, and SEM images.

In this work, particle-size regulation of **CP1** and **CP2** was achieved by combining a bottom-up synthetic strategy, with a complementary top-down size-reduction step. This dual approach enables control over crystal growth while maintaining the structural identity of the CPs. Both CPs were synthesized at room temperature (**CPs-RT**) and in the presence of polyvinylpyrrolidone (**CPs-PVP**), replacing traditional solvothermal conditions.

Morphological evolution reflects the intrinsic structural anisotropy of each CP. **CP1-RT** forms elongated prismatic ribbons (≈18 ± 8 μm), consistent with its 1D chain propagation. In contrast, **CP2-RT** produces microspherical aggregates composed of stacked lamellae (≈1.15 ± 0.04 μm), in agreement with its 2D layered structure (Figures 2, S3 and S4).

When synthesized with 7% w/v PVP, both materials form aggregates of nanofibers (**CP1-PVP**) or nanosheets (**CP2-PVP**) (Figures 2, 3, S3 and S4), which can be readily dispersed by brief US (5 min at 80% power). This treatment disrupts the van der Waals interactions responsible for the cohesion of the aggregates (Figures 2, 3, S3 and S4). As a result, a substantial reduction in particle size was achieved, reaching a length and height (1.5 ± 0.4 × 0.06 ± 0.02) μm for **CP1-PVP** and (0.30 ± 0.04 × 0.07 ± 0.04) μm for **CP2-PVP** (Figures 3, S3 and S4). Once again, a clear correlation with the structural dimensionality of each CP can be observed: **CP1-PVP** predominantly forms nanofibers, whereas **CP2-PVP** yields nanosheets, in good agreement with the one-dimensional crystalline structure of **CP1** and the two-dimensional crystalline structure of **CP2**. Demonstrating that surfactant-assisted synthesis enables substantial downsizing while preserving the crystalline framework.

**Figure 3:**
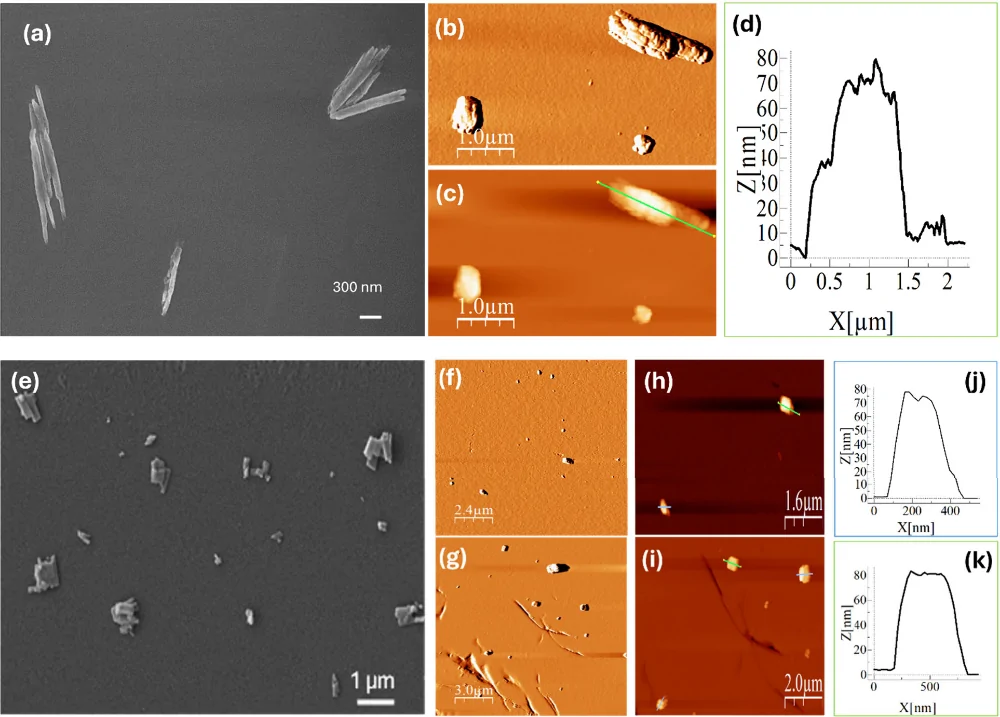
SEM images of (a) **CP1-PVP** and (e) **CP2-PVP**. (b, c) AFM images of **CP1-PVP** and (d) its height profile. (f–i) AFM images of **CP2-PVP** and (j, k) its height profiles.

While water provides a sustainable reaction medium and promotes rapid nucleation, PVP plays a decisive role in limiting crystal growth and preventing aggregation, functions as a structure-directing and stabilizing agent during particle formation. The carbonyl groups of PVP can weakly interact with the surface of the forming Cu­(I)-iodide coordination polymer nuclei, generating a steric barrier that hinders further particle coalescence. At the same time, the nonionic and water-soluble nature of PVP ensures colloidal stabilization of the resulting particles, allowing the formation of homogeneous and persistent aqueous suspensions The PVP concentration was selected through preliminary optimization experiments using 1, 3, 5, and 7% w/v PVP. Lower concentrations produced less homogeneous morphologies and poorer colloidal stabilization, whereas 7% w/v consistently yielded nanometric particles and homogeneous, persistent aqueous suspensions. Accordingly, 7% w/v PVP was selected as the optimal condition for reproducible particle downsizing and dispersion stability. Elemental analysis suggests that a small amount of PVP remains associated with the particle surface after washing. If the carbon excess is fully attributed to residual PVP, the estimated polymer content is ca. 1 wt %. Thus, PVP is present as a minor surface contribution rather than as a major organic coating of the material. PXRD and FTIR-ATR analyses confirm that incorporation of PVP does not alter the crystalline phase of either CP, although slight peak broadening indicates an increase in defect density (Figures S1 and S2). This finding indicates that the reduction in crystal size leads to a higher density of structural defects, which can be attributed to the growth-inhibiting effect of PVP acting as a capping agent during material formation, as widely reported for polymer-assisted syntheses.^23,24^

To further tune particle dimensions, complementary top-down methods were applied. Ultrasonic treatment of **CPs-RT** samples (**CPs-US**) in a bath for 1 h at room temperature and a power of 80% in induces cavitation-driven fragmentation and partial exfoliation. **CP1-US** shows breakage of micrometric rods into thinner lamellar fragments, whereas **CP2-US** undergoes disassembly of microspherical aggregates into nanosheets. (Figures 2, S3 and S4). AFM measurements reveal sheet thicknesses as low as ≈60 ± 40 nm for **CP2-US** (Figure 4). PXRD and FTIR-ATR analyses confirm that the coordination frameworks remain intact, although Bragg peak broadening indicates smaller coherent domains and increased defect density. (Figures S1 and S2). These findings indicate that ultrasonication not only reduces the particle size of both CPs to the submicrometric regime but also promotes exfoliation and may induce defect formation (particularly at edges/terminations), which can significantly impact their optoelectronic properties.^25,26^

**Figure 4:**
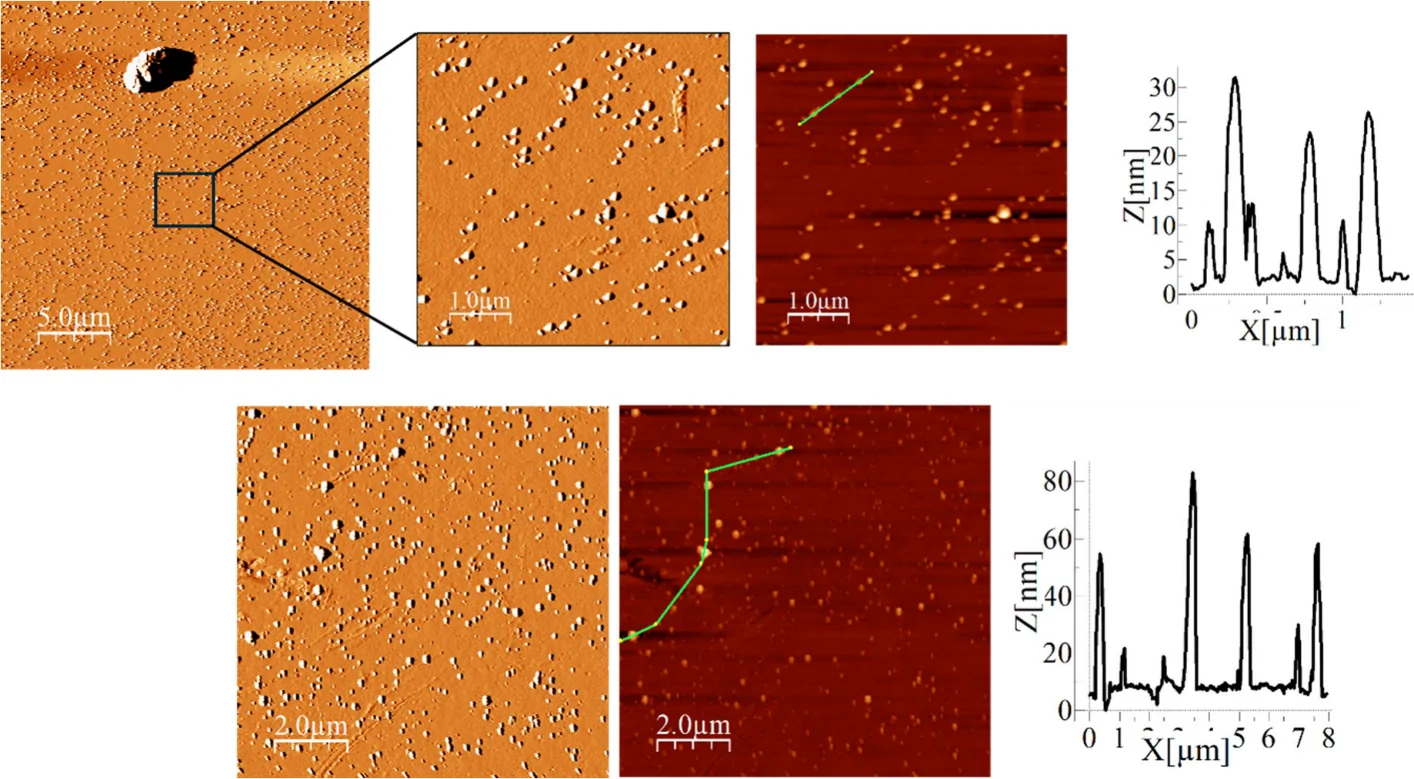
AFM images of **CP2-US**, depicting the height, length, and width of randomly selected particles deposited on a quartz surface.

Finally, mechanical grinding (**CPs-GR**) provides an alternative route to nanoscale particles (≈200 ± 100 nm) through shear and compressive forces. PXRD patterns show attenuation and broadening of reflections, indicating partial loss of long-range order (Figures 2, S3 and S4) while preserving local coordination environments. This mechanically induced disorder introduces additional structural defects that may modulate optoelectronic properties, (Table 1).^27,28^

**Table 1:** Morphology, Dimensions, Band Gap, and Emission Values of **CP1** and **CP2** Obtained Using Different Approaches

Compound	Morphology	Dimensions: length × width × height (μm)	Band gap (eV)	Excitation/emission (nm)
**CP1-RT**	Ribbons	18 ± 8 × 2.2 ± 0.7	2.51	345/440
**CP1-PVP**	Nanofibers	1.5 ± 0.4 × 0.10 ± 0.03 × 0.06 ± 0.02	2.07	355/445 and 615 (shoulder)
**CP1-US**	Ribbons/sheets	14 ± 6 × 1.9 ± 0.6	2.80	330/438
**CP1-GR** ^a^	Grains	0.2 ± 0.1	3.18	330/515
**CP2-RT**	Microspheres/sheets	1.15 ± 0.04 × 0.73 ± 0.03	2.32	500/700
**CP2-PVP**	Nanosheets	0.30 ± 0.04 × 0.20 ± 0.03 × 0.07 ± 0.04	2.23	500/685
**CP2-US**	Nanosheets	1.5 ± 0.1 × 0.63 ± 0.08 × 0.06 ± 0.04	2.35	500/700
**CP2-GR**	Grains	0.2 ± 0.1	2.42	460/720
**CP2-ST**	Sheets	32 ± 3 × 15 ± 1	2.28	500/590

a **CP1-GR** loses its emission after 48 h.

### Photoluminescence and Band-Gap Modulation as a Function of Size and Structural Perturbation

It has been well established that variations in particle morphology and size profoundly influence their electronic and optical properties (Table 1), primarily due to the higher defect density and increased surface-to-volume ratio achieved at the nanometer or submicrometer scales.^28^ As particle dimensions decrease, the rearrangement of energy levels induces both band gap shifts and the formation of localized electronic states, which can either enhance or attenuate the material’s luminescence.^29^

Particle size reduction also increases surface contact area and promotes mixture homogeneity, thereby facilitating the formation of composite materials. In liquid–solid suspensions, such mixtures remain stable over extended periods, enabling seamless integration into device fabrication processes across various technological platforms. Furthermore, smaller particles can be more easily incorporated into porous supports or substrates, considerably expanding the potential for novel device architecture.

Within this framework, the rational design of nanometric particles not only modulates and optimizes their electronic and optical features but also ensures homogeneous dispersion. This results in enhanced efficiency in applications such as analytical detection and photocatalytic degradation of pollutants or antibiotics.

#### Luminescence Analysis

Cu­(I)-I CPs with double-ladder motifs exhibit emission properties governed by the interplay between charge-transfer states involving halide and copper centers, as well as by cuprophilic (Cu···Cu) interactions. The photophysics of such systems are typically described in terms of XMLCT/XLCT transitions (halide/metal → ligand), cluster-centered (CC) states within the Cu–I core, and where relevant, metal-to-ligand charge transfer (MLCT) contributions. The relative contributions of these processes strongly depend on the material’s dimensionality and the specific arrangement of Cu_2_I_2_ units within the coordination network.^30,31^

In **CP1**, which incorporates a pyridine ligand, the Cu_2_I_2_ motifs remain relatively isolated along the double chains. This separation favors high-energy (blue) emissions associated with XMLCT and CC states, characterized by limited structural relaxation and reduced Cu···Cu coupling (Table 1 and Figures S5 and S6).^32^ Both **CP1-RT** and **CP1-US** exhibit maximum emission near 440 nm (Table 1 and Figures S5 and S6). By contrast, **CP1-PVP** displays an additional band centered at 615 nm, while **CP1-GR** shows a broad emission band around 515 nm (Table 1 and Figures S5 and S6). This behavior indicates a greater contribution of Cu···Cu interactions to the electronic transitions. The angular compression of the Cu–I–Cu bridges increases the orbital overlap and strengthens cuprophilic Cu···Cu interactions, stabilizing lower-energy excited states and shifting the emission toward longer wavelengths. In addition, structural defects generated within the CP can introduce localized emissive states associated with distortions of the Cu–I framework. In samples prepared with PVP, the formation of nanoparticles mainly introduces surface-related defects that act as additional emissive centers, giving rise to a shoulder at longer wavelengths. In contrast, mechanical grinding produces a higher degree of structural disorder throughout the material, generating a wider distribution of defect states and consequently a significantly broadened emission band (Table 1 and Figures S5 and S6). These effects promote extended Cu···Cu coupling, shifting emission toward lower energies.^33^ Notably, the emission of **CP1-GR** progressively disappears after approximately 48 h under ambient conditions. PXRD and FTIR-ATR analyses recorded immediately after grinding, when **CP1-GR** was still emissive, and after 48 h, when the emission had disappeared, showed no significant changes in the main crystalline and vibrational features. This suggests that the emission loss is not associated with major crystalline phase degradation, bulk framework collapse, or detectable ligand loss. Instead, it is consistent with the gradual relaxation or reorganization of metastable local emissive environments generated by mechanical treatment, involving local Cu–I distortions and changes in Cu···Cu interactions, which contribute to the broad 515 nm emission band. Similar mechanoresponsive Cu­(I) and Cu­(I)-iodide systems have shown that mechanical stimuli can strongly modify luminescence through local framework distortions and changes in CC excited states without necessarily inducing complete structural collapse.^34,35^

In **CP2**, which contains a pyrazine ligand, the N-donor bridging within the two-dimensional sheets enhances Cu­(I) connectivity and strengthens cuprophilic interactions, leading to lower-energy (orange-red) emissions typical of CC states with pronounced Cu···Cu participation (Table 1 and Figures S5 and S6).^18^ This behavior is consistent with the known sensitivity of Cu­(I)-iodide CP emission to subtle structural variations in the inorganic framework and to the balance between charge-transfer and cluster-centered excited states. The major difference is observed for the solvothermally synthesized **CP2-ST** sample, which exhibits a higher-energy yellow emission at 590 nm (Table 1 and Figures S5 and S6). In contrast, **CP2-RT**, **CP2-PVP**, **CP2-US** and **CP2-GR** emit in the orange-red region, with maxima between 685 and 720 nm. Thus, these samples show a pronounced red shift relative to **CP2-ST**, although the differences among them are comparatively smaller. The magnitude of this shift suggests that **CP2-ST** and the remaining **CP2** samples differ not only in particle size, but also in their local Cu–I emissive configuration; the solvothermal route appears to favor a more ordered, higher-energy emissive state, whereas the room-temperature and processed samples favor stronger excited-state relaxation and a larger contribution of low-energy cluster-centered/cuprophilic emission. This behavior suggests that, unlike **CP1**, the emissive response of **CP2** is mainly governed by its layered Cu–I connectivity, which maintains effective electronic communication between the clusters and, consequently, a similar low-energy emission profile across the room-temperature and differently processed samples despite particle downsizing and mechanical treatment.

#### Band-Gap Analysis

Diffuse-reflectance spectroscopy (DRS), analyzed via the Kubelka–Munk transformation and Tauc plots, owed morphology-dependent variations in the optical band gap energy (Figures S7–S10). For both CP families, the best fits correspond to a direct electronic transition model. The resulting apparent optical band gaps reflect the balance between structural coherence, electronic coupling, and defect-related states.

**CP1** morphologies show a marked increase in the optical band gap upon mechanical processing. This behavior correlates with the progressive reduction of coherent domain size and the higher density of structural defects introduced during particle downsizing.^28^ In the double-ladder architecture of **CP1**, these perturbations can partially disrupt the connectivity between Cu_2_I_2_ motifs, weakening cuprophilic Cu···Cu interactions and reducing electronic delocalization along the chain. Therefore, the effective electronic coupling between clusters decreases, leading to a widening of the optical band gap. The increase in the optical band gap observed after grinding or sonication is more consistently associated with the loss of structural coherence and the weakening of Cu···Cu/Cu–I electronic interactions, which decreases electronic delocalization within the Cu–I framework. In these mechanically processed samples, the resulting defects are likely distributed throughout the bulk of the material, disrupting cluster connectivity and long-range electronic communication across the network. By contrast, nanoparticle formation in the presence of PVP likely introduces mainly surface-localized defect states that generate sub-band gap absorption, leading to a lower apparent optical band gap. These surface defects have a more localized character and mainly affect the outer regions of the particles without significantly disrupting the overall electronic connectivity of the coordination framework. Therefore, the final trend results from the competition between defect-induced localized states that reduce the apparent optical gap and the extent of electronic communication preserved in the Cu–I network, whose disruption leads to band gap widening (Table 1).

**CP2** morphologies exhibit a similar but less pronounced trend when compared with **CP1**. The highly ordered solvothermal sample (**CP2-ST**) is taken as the reference material and displays a band gap of ≈2.28 eV, consistent with its extended two-dimensional connectivity that promotes strong Cu···Cu interactions and efficient electron delocalization across the Cu–I layers. In **CP2-PVP**, particle downsizing is accompanied by a slight decrease in the band gap energy, likely associated with the formation of surface-related defect states that introduce intermediate electronic levels within the gap. In contrast, the morphologies obtained through mechanical processing exhibit a small increase in the band gap energy due to the structural disorder introduced in the Cu–I framework, which partially weakens the electronic coupling between clusters. However, these variations remain modest compared to **CP1**, as the layered topology of **CP2** preserves efficient cluster communication even in the presence of local defects, as also observed for the luminescence behavior. In contrast, the 1D architecture of **CP1** is more susceptible to the loss of structural coherence, leading to a more pronounced increase in the band gap energy.

#### Theoretical Characterization of the Electronic Structure

The optical behavior of **CP1** and **CP2** can be rationalized from their electronic structures by considering the dimensionality and defect-induced states on their surface. In these Cu–I CPs, the valence-band edge (HOMO orbital) is mainly composed of hybridized I and Cu states, while the conduction-band edge (LUMO) includes is mainly characterized by the ligand states. As a result, the band gap is highly sensitive to both Cu–I connectivity and local coordination defects. For pristine **CP1**, the calculated DOS shows a well-defined gap, consistent with a relatively localized electronic structure associated with its 1D arrangement, as shown in Figure S11. This limited delocalization and the absence of a high dimensional network make **CP1** particularly sensitive to structural perturbations. When one iodine atom is removed, new states appear between the valence and conduction band, leading to a clear reduction of the calculated band gap (Figure S12), due to local rearrangement of the Cu–I chain (Figure 5). In this compound, with periodicity along only 1D, defects are more common, leading to smaller band gaps, in good agreement with the experimental measurements. The relatively low formation energy of the iodine vacancy (0.9 eV), as calculated by DFT, supports the feasible formation of these defects.

**Figure 5:**
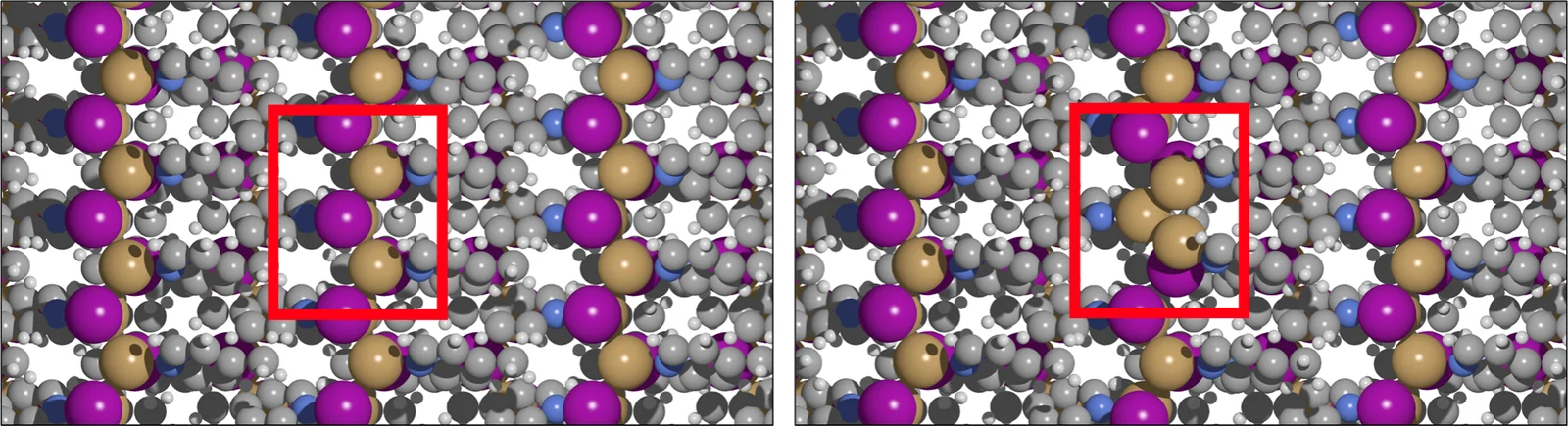
Optimized structure of **CP1** without defects (left) and with a I vacancy (right). The red square indicates the region where the atomic rearrangement takes place. Color code: H, white; C, gray; N, blue; Cu; brown; and I, purple.

On the other hand, in the case of **CP2**, the structure is more robust due to the 2D network and less sensible to defect formation, reason why the band gap barely changes with the synthetic procedure (Table 1). In this compound, as in **CP1**, the valence band is mainly defined by Cu–I orbitals, and the conduction band is localized on the organic ligand (Figure S13). **CP2** exhibits a lower band gap, due to the lowest LUMO energy of the pyrazine ligands.

### Application as Heterogeneous Photocatalysts in Organic Dyes: Methyl Orange Degradation

The band gap values and water stability of **CP1** and **CP2** make these CPs suitable heterogeneous photocatalysts for the degradation of persistent organic dyes. Photocatalytic experiments were conducted by dispersing the CPs in aqueous solutions of methylene blue (MB), methyl orange (MO), and rhodamine B (RhB) under white-light irradiation. Dye degradation was monitored by measuring the decrease in UV–vis absorbance at the characteristic absorption band after catalyst removal.

The structural stability of the CPs under photocatalytic conditions was independently evaluated by ^1^H NMR spectroscopy. These experiments were performed at a higher MO concentration (10^–3^ M) than the UV–vis degradation tests and were intended to assess possible ligand leaching, rather than to quantify dye degradation. Spectra recorded at t = 0 and after 90 min irradiation in D_2_O, both with and without MO, showed no signals attributable to free ligands, confirming framework preservation without detectable ligand leaching (Figures S14 and S15).

The kinetics of MO degradation were evaluated using samples with different particle sizes. Under the selected conditions, MO degradation was best described by an apparent zero-order kinetic model. This behavior should not be interpreted as evidence that the reaction rate is intrinsically independent of dye concentration or that the catalyst surface remains fully saturated throughout the process. Over the short reaction times investigated, and at fixed catalyst loading and irradiation, the observed rate may instead be governed by photon absorption, charge-carrier generation, oxygen activation, or reactive-species availability. Within the Langmuir–Hinshelwood framework, apparent zero-order behavior may arise at high surface coverage, but similar responses can also result from limitations in photon flux, active-site availability, or oxygen mass transfer.^36^ Accordingly, the zero-order fit is considered an operational description of the kinetic regime observed under the present experimental conditions. **CP1-RT** and **CP1-US** achieved complete disappearance of the characteristic MO absorption band within ∼25 min, whereas the smaller-particle materials **CP1-PVP** and **CP1-GR** required only 5 and 3 min, respectively, displaying kinetic constants approximately 1 order of magnitude higher (Table 2, Figure 6a). For **CP2**, the effect of particle size was less pronounced, due to the rapid degradation time. **CP2-ST** showed slower degradation (8 min) compared with ∼3 min for the others (Table 2, and Figure 6b).

**Figure 6:**
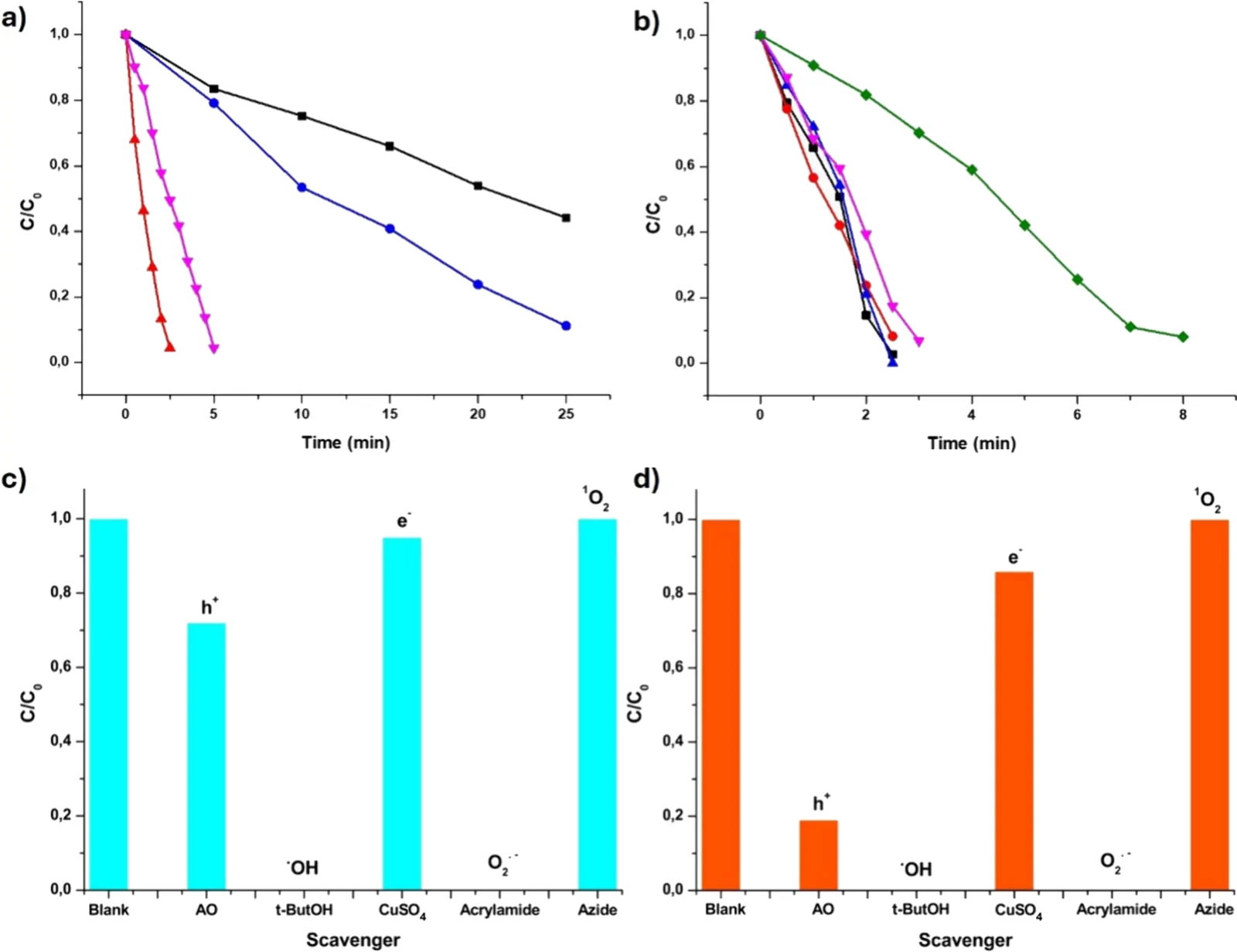
Decay curves for MO at different morphologies of (a) **CP1** and (b) **CP2**: **CP-RT** (black line), **CP-PVP** (red line), **CP-US** (blue line), **CP-GR** (pink line) and **CP2-ST** (green line). Control experiments in the presence of scavengers in the MO degradation by (c) **CP1** and (d) **CP2**.

**Table 2:** MO Photodegradation Performance of Some Semiconductors Published in the Literature^a^

Compound	MO (mg/L)	MO degradation time (min)/cycles	mg photocatalyst/mL dye	Band gap (eV)
TiO_2_ ^39^	2000	240/8	2000/80	3.20
[Cu_2_(OH)(mbtx)(sip)(H_2_O)_2_]_n_ /{[Cu_3_(OH)_2_(mbtx)(nip)_2_]·H_2_O_n_ ^40^	10	180/n.r.	6.4/200	2.52/2.59
{[Cu(HL)(5-MeIPA)]·0.25H_2_O}_n_ ^41^	20	300/n.r.	5/30	3.08
{[Zn_4_(H_2_O)_2_(4,4′-bipy)_**4**_(glu)_3_](NO_3_)_2_}_n_ ^42^	2.5	300/2	400/100	3.56
Cu(dm)-(bim)^43^	18.7	45/3	30/10	2.49
[Cu_2_I_2_(BPEE)]_n_ ^44^	3	60/6	2/2	2.04
[(CuI)(4-dpye)_0.5_]_n_ ^45^	20	240/3	10/50	2.68
[(Cu_4_I_4_)(3-bpah)_2_]·3CHCl_3_·MeCN}_n_ ^45^	20	240/3	10/50	2.45
**CP1-RT**	10	25/3	2/2	2.51
**CP2-RT**	10	3/7	2/2	2.32

a n.r.= not reported; HL = 4′-(4-hydroxyphenyl)-4,2′:6′,4″-terpyridine; 5-Me-IPA = 5-methyl-iso-phthalic acid; glu = glutarate; 4,4′-bipy = 4,4′-bipyridine; mbtx = 1,3-bis­(1,2,4-triazol-4-yl)­benzene; sip = sulfoisophthalate; nip = 5-nitro­iso­phth­alate; dm-bim = 5,6-dimethyl­benz­imid­azolate; BPEE = 1,2-di­(pyridin-2-yl)­ethane; 4-dpye = *N*,*N*′-bis­(4-pyri­dine­carb­oxamide)-1,2-ethane; 3-bpah = *N*,*N*′-bis­(3-pyri­dine­carb­oxamide)-1,2-cyclohexane.

Since equal catalyst masses contain slightly different amounts of Cu, the photocatalytic performance of **CP1-RT** and **CP2-RT** was additionally compared after correction for their theoretical Cu contents. **CP1** and **CP2** contain 22.41 and 26.75 wt % Cu, respectively, corresponding to 7.05 and 8.42 μmol of Cu in the 2 mg catalyst loading. Taking the Cu amount in **CP1-RT** as the reference, the degradation time of **CP2-RT** was corrected from 3.0 to 3.58 min (3.0× (8.42/7.05)). Thus, **CP2-RT** remains approximately 7 times faster than **CP1-RT** after Cu-content normalization. This comparison suggests that the photocatalytic response is not determined solely by the amount of Cu present but also depends on framework- and morphology-related properties, including Cu–I connectivity, dimensionality, electronic structure, particle dimensions, and accessibility of the external particle-solution interface.

SEM and AFM analyses confirmed a marked reduction in particle dimensions. The enhanced photocatalytic performance of the smaller-particle samples may therefore be associated with improved accessibility of the external particle-solution interface and more effective contact with MO molecules. Although processing also modifies the electronic structure and, in some cases, increases the optical band gap through defect formation and loss of long-range connectivity, all samples absorb within the irradiation range employed. The observed activity therefore likely reflects a balance between morphology-dependent interfacial accessibility and processing-induced changes in the electronic structure. This coherent particle-size-dependent trend suggests that residual surface PVP does not dominate the photocatalytic response.

For direct comparison with the UV–vis experiments, ^1^H NMR analyses were performed under identical conditions (MO concentration: 10^–5^ M; catalyst loading: 2 mg; solution volume: 2 mL), and the corresponding spectra are shown in Figures S17 and S18. After irradiation with **CP1** or **CP2**, no aromatic signals attributable to residual MO or free ligands were detected, consistent with MO degradation without detectable ligand leaching. Weak signals appeared mainly in the aliphatic region, supporting disruption of the azo-conjugated structure. However, individual products could not be assigned because they are likely present as a low-concentration mixture with overlapping resonances. The different spectral profiles obtained with **CP1** and **CP2** therefore suggest differences in the relative abundance of the intermediates or products formed.

Recyclability tests revealed a gradual loss of activity for **CP1-RT**, reaching ∼45% degradation efficiency after three cycles (Figure S19). In contrast, **CP2-RT** maintained higher photocatalytic performance over multiple cycles, retaining more than 50% efficiency up to the seventh cycle (Figure S20). PXRD and FTIR-ATR analyses after catalysis confirmed the overall preservation of the coordination frameworks. However, recycled **CP1-RT** shows weak additional reflections in the PXRD patterns, suggesting a partial structural rearrangement or the formation of a minor secondary crystalline phase during cycling (Figure S21). Although this phase could not be unambiguously assigned from PXRD alone, the preservation of the main **CP1** reflections, together with FTIR-ATR and ^1^H NMR stability experiments, indicates that it is not associated with complete framework collapse or detectable ligand leaching. This partial reorganization of the Cu–I framework may contribute to catalyst deactivation. By contrast, **CP2-RT** remains essentially unchanged after catalysis, indicating higher structural stability, likely associated with its more robust 2D pyrazine-bridged architecture (Figure S22). Therefore, **CP2** deactivation is more plausibly related to progressive blocking of active sites by adsorbed intermediates/products rather than framework transformation.

Control experiments performed in the absence of either light or catalyst showed no degradation, confirming the photocatalytic nature of the process. Reactive-species trapping experiments were carried out to elucidate the reaction mechanism (Figure 6c and 6d).^37,38^ For both **CP1-RT** and **CP2-RT**, MO degradation was strongly suppressed in the presence of NaN_3_ (singlet oxygen scavenger) and CuSO_4_ (electron scavenger), while partial inhibition was observed with ammonium oxalate (hole scavenger). These results indicate that electrons (e^–^), holes (h^+^), and singlet oxygen (^1^O_2_) are involved in the degradation mechanism. Upon irradiation, photoexcitation of the CPs generates electron–hole pairs. Photogenerated holes can directly oxidize adsorbed MO molecules, weakening the azo (—N=N—) chromophore, as supported by the inhibition observed with ammonium oxalate. In parallel, photogenerated electrons participate in oxygen activation, as indicated by the strong inhibition caused by CuSO_4_. The formation of ^1^O_2_, supported by the NaN_3_ trapping experiment, may arise from energy transfer from photoexcited Cu­(I)-iodide/ligand-based states to ground-state triplet oxygen. However, the effect of CuSO_4_ indicates that electron-mediated oxygen activation also contributes, and therefore ^1^O_2_ formation should not be assigned exclusively to a classical triplet-sensitization pathway. Although processing-induced changes in the optical band gap may affect light absorption and charge-separation efficiency, all CP samples absorb within the broad 300–600 nm irradiation used, so both energy-transfer and electron-mediated oxygen-activation pathways remain accessible under the experimental conditions. The synergistic action of h^+^, electron-mediated oxygen activation and ^1^O_2_ results in progressive dye degradation into lower-molecular-weight products. The preferential degradation of MO over MB and RhB may arise from the combined effect of the anionic nature of MO, which may favor its interaction with the CP surface compared with the cationic dyes, and the greater susceptibility of the azo chromophore to oxidative cleavage by photogenerated holes and ^1^O_2_.

Comparison with previously reported photocatalysts for MO degradation shows that **CPs-RT** exhibit comparable or superior performance, together with good stability and recyclability (Table 2). Their relatively low band gaps enable efficient activation under visible-light irradiation, highlighting their potential for practical photocatalytic applications.

### Antibiotic Sensor Application

#### Tetracycline Detection, Selectivity, and Mechanism

Antibiotic detection through emission quenching was evaluated using **CP1-US** and **CP2-US**, as these materials provide the optimal balance between colloidal stability and luminescence retention (Figure S23). Sonication produces smaller, fragmented particles that form stable aqueous suspensions without altering the intrinsic emissive state. In contrast, **CP1-PVP** and **CP1-GR** samples exhibit an additional emission shoulder associated with enhanced cuprophilic (Cu···Cu) interactions, which complicates quenching analysis; therefore, these materials were excluded from sensing studies. The sensing performance of **CP-US** particles was investigated using three widely used antibiotics from different families: sulfametazine (SMZ), chloramphenicol (CAP), and tetracycline (TC). Aqueous suspensions maintained nearly constant emission intensity after addition of SMZ or CAP (0.80 μmol, 10^–3^ M, 0.8 mL). In contrast, the presence of TC (0.80 μmol, 400 μM, 2.1 mL) induced a pronounced decrease in luminescence intensity, demonstrating selective TC detection in water and highlighting **CP-US** materials as effective luminescent sensors.

The interaction between **CP-US** particles and TC was quantified by monitoring the normalized emission intensity as a function of analyte concentration in deionized water. Within the 0–250 μM range, the data follow a linear Stern–Volmer relationship,^46^ indicating an efficient apparent fluorescence quenching response under the selected sensing conditions (Figures 7 and S24–S29). The apparent Stern–Volmer constants (*K*_*SV*_) were determined as *K*_*SV*_ = 1.95 × 10^4^ M^–1^ for **CP1-US** and *K*_*SV*_ = 5.29 × 10^3^ M^–1^ for **CP2-US**. Detection limits (LODs), calculated according to the IUPAC criterion,^47^ were 410 nM and 2806 nM for **CP1-US** and **CP2-US**, respectively. Sensor recyclability was evaluated over ten consecutive TC exposure cycles. PXRD and IR spectroscopy analyses after cycling retained the characteristic features of both materials, confirming preservation of structural integrity and suitability for repeated sensing applications (Figures S30 and S31).

**Figure 7:**
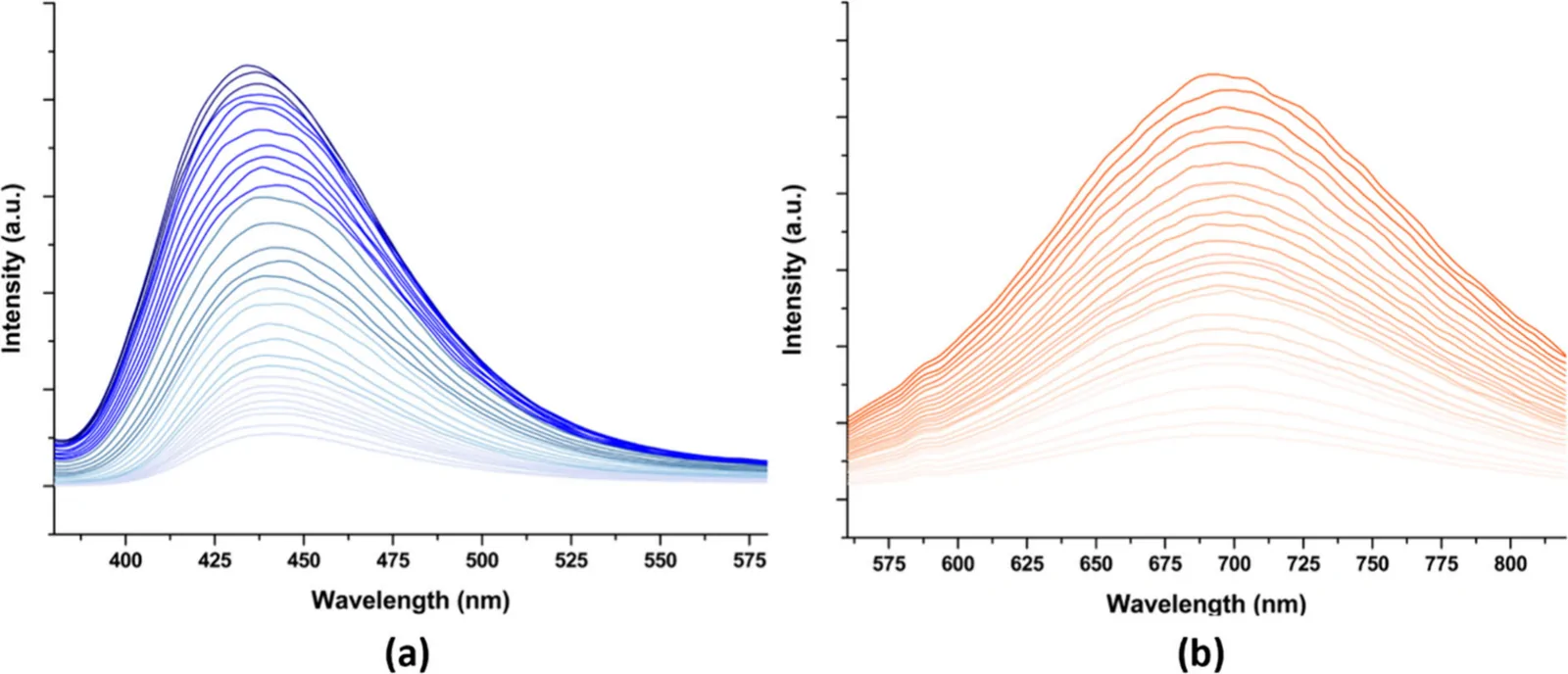
Emission spectrum of aqueous suspensions of (a) **CP1-US** and (b) **CP2-US** as a function of the concentration of different TC additions. Measurements were performed in deionized water at room temperature, using an excitation wavelength of λ_ex_ = 365 nm. The TC concentration range was 0–250 μM. The CP suspension concentration was 1 mg mL^–1^. Suspensions were freshly sonicated before use and vortexed before each measurement to maintain homogeneity.

Sensor selectivity was further assessed against SMZ and CAP, commonly encountered interferents in aqueous TC detection studies. No significant emission changes were observed upon exposure to these antibiotics, whereas TC produced strong luminescence quenching (Figures S32 and S33), confirming the high selectivity of both systems. Among the fluorescence quenching pathways commonly reported for CP-based sensing systems, photoinduced electron transfer (PET) and inner filter effect (IFE) are the most relevant mechanisms for antibiotic detection. PET can occur when suitable energetic alignment between the excited CP and the analyte enables charge transfer, thereby suppressing radiative recombination.^48^ DFT calculations for **CP1** indicate a valence band at −4.00 eV and a band gap of 2.70 eV, corresponding to an estimated conduction band position of −1.30 eV. For **CP2**, the valence and conduction band energy levels were calculated at −5.06 and −3.35 eV, respectively. Reported HOMO/LUMO levels for TC are −6.11 and −2.62 eV, respectively.^12^ Since the conduction band of **CP1** lies at a higher electronic energy level than the LUMO of TC, electron transfer from photoexcited **CP1** to TC is energetically favorable, supporting a PET-mediated quenching pathway (Figure S34). In contrast, the energetic alignment between **CP2** and TC does not favor an efficient PET process. The contribution of IFE was also evaluated through spectral overlap analysis.^49^ As shown in Figures S35 and S36, in both **CP1-US** and **CP2-US**, the absorption spectrum of TC significantly overlaps with the absorption region of the CPs around the excitation wavelength (365 nm), indicating that TC can effectively compete for the excitation light and induce a primary IFE process. This overlap is complete for **CP1-US**, whereas for **CP2-US** it is partial but remains particularly significant at the excitation wavelength. By contrast, only minor overlap is observed between the TC absorption spectrum and the emission band of **CP1-US**, while no appreciable overlap is detected for **CP2-US**, indicating a negligible contribution from secondary IFE processes. Accordingly, the fluorescence quenching of **CP1-US** is mainly attributed to primary IFE, with a possible additional PET contribution due to the favorable energetic alignment between **CP1** and TC. For **CP2-US**, where PET is energetically unfavorable, the quenching process is most plausibly dominated by primary IFE, consistent with its lower apparent Stern–Volmer constant compared to **CP1-US**. Therefore, the sensing response should be interpreted as an apparent quenching process mainly arising from spectral filtering, with possible contributions from CP-TC interactions, rather than as a purely molecular-scale static or dynamic quenching mechanism.

The obtained *K*_*SV*_, detection limits, and cycling stability compare favorably with previously reported coordination polymer sensors (Table 3). Many literature examples exhibit higher detection limits, limited recyclability, or require more demanding synthetic conditions. Consequently, **CP1-US** and **CP2-US** particles emerge as efficient, selective, and reusable luminescent sensors for tetracycline detection in aqueous media.

**Table 3:** Comparison of *K*_*SV*_, Recyclability, and LOD Obtained in This Work for the Detection of TC in Aqueous Media with Other Published Data^a^

Coordination compounds	*K*_*SV*_ (M^–1^)	LOD (nM)	Recyclability
MOF Eu-TCPE^50^	1.49 × 10^5^	14.0	n.r.
[Zr_6_O_4_(OH)_4_(ETTC)_3_][Bibr ref51]	9.84 × 10^5^	30	n.r.
[Zn_3_(L)_2_(1,4-bimb)_3_]_n_ ^52^	1.98 × 10^5^	150	n.r.
[Cd(opda)(tib)]·H_2_O^48^	3.85 × 10^5^	560	n.r.
[Cu_4_I_4_(ETBT)_4_][Bibr ref53]	3.23 × 10^3^	4150	5
**CP1-US**	1.95 × 10^4^	410	10
**CP2-US**	5.29 × 10^3^	2806	10

a n.r. = not reported; TCPE = tetrakis­(4-carboxy­phenyl)­ethylene; ETTC = 1,1,2,2-tetrakis­(4-carboxy­phenyl)­ethene; L = 5-(2-carboxyl­phenoxy)­iso­phth­alate; 1,4-bimb = 1,4-bis­(imidazol-l-ylmethyl)­benzene; H_2_opda = phenylenediacetates; tib = 1,3,5-tris­(1-imidazolyl)­benzene; ETBT = 2-ethylbenzo­[d]­thiazole.

#### Processing to Form Sensor Devices

**CP1-US** and **CP2-US** form stable aqueous suspensions (Figure S23), enabling their processing into solid-state sensing platforms through different fabrication strategies. Composite laminar films were prepared using polylactic acid (PLA) as a biodegradable polymer matrix. Drop-casting films (**CPs@DC**) were obtained by dissolving PLA in either dioxane or acetonitrile at 60 °C, followed by incorporation of the corresponding CP. When dioxane was used, stable dispersions of CP particles were maintained within the polymer solution. In contrast, partial solution of the CP occurred in acetonitrile; subsequent solvent evaporation induced recrystallization of the material within the polymer matrix (Figures S37 and S38). Additionally, composite devices were fabricated using a commercial photopolymer resin via 3D printing (**CPs@3D**) (Figures 8 and S39).

**Figure 8:**
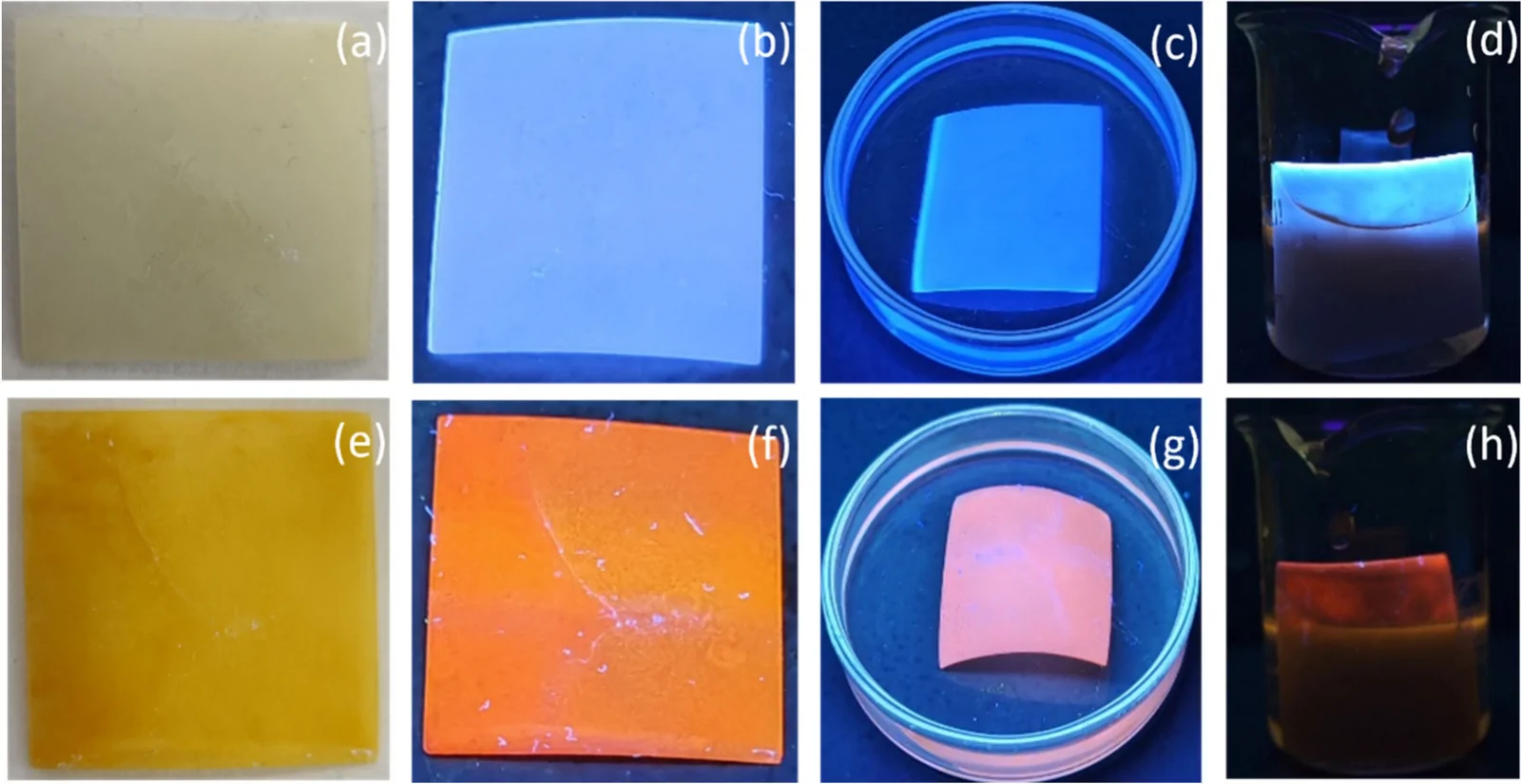
**CP1-US** (top) and **CP2-US** (bottom) portable devices: (a, e) **CPs@3D** viewed under visible light, (b, f)**CPs@3D** viewed under UV light at 365 nm, (c, g) **CPs@3D** in water, and (d, h) **CPs@3D** in 400 μM aqueous solution of TC.

PXRD patterns confirmed that both CPs retain their crystalline structure after incorporation into the polymer matrices, as the main characteristic reflections of the CPs remain observable (Figures S40–S43). Although the IR spectra are dominated by the bands of the polymeric matrices, the preservation of the CP frameworks is consistent with the PXRD results. Structural integrity was preserved for all matrices except for **CP1** films prepared from PLA/dioxane. In the case of **CP1@DC**, PXRD revealed a phase transition associated with the formation of a new crystalline phase, accompanied by a change in emission color. This behavior is consistent with the reported solvatochromic response of **CP1** ^6^ and indicates a direct correlation between structural modification and photophysical properties. Dioxane appears to induce a structural reorganization of **CP1** during film formation, modifying the Cu–I framework and leading to the observed emission change. No phase transformation was observed for **CP2@DC** under analogous conditions. The CP particles are homogeneously distributed in the 3D-printed composites, whereas films prepared from PLA/acetonitrile solutions show heterogeneous dispersion, attributed to partial solubilization/redistribution of the CP followed by the formation of recrystallized domains from multiple nucleation sites during solvent evaporation. The main PXRD reflections and emission color of these recrystallized domains are consistent with the corresponding parent CP phase, indicating that no dominant free ligand or different crystalline Cu–I phase is formed during solvent evaporation. This recrystallization-driven heterogeneous distribution is absent when particles remain suspended prior to film formation, as observed for films prepared in PLA/dioxane. SEM-EDX analysis confirmed successful incorporation of the CPs into all composite materials through detection of characteristic Cu and I signals within the polymer phase (Figures S44–S48).

As shown in Figures 8 and S49, device emission intensity remains unchanged after exposure to 20 mL of water, whereas rapid and complete attenuation of the detected luminescence occurs upon contact with a 400 μM aqueous tetracycline solution. This behavior supports an important contribution of IFE, since complete emission attenuation in the polymer composites cannot be explained solely by direct molecular contact between TC and all embedded emissive CP domains. In particular, a part of the CP is embedded within the polymer matrix, and SEM images show that the accessibility depends on the fabrication method: the 3D-printed composites display small pores/voids that may facilitate local access of TC to surface or near-surface CP particles, whereas the PLA drop-cast films do not show evident interconnected channels that would allow rapid penetration throughout the whole matrix. Therefore, if molecular contact were the only quenching pathway, these internal emissive domains would be expected to retain part of their emission. Instead, TC absorption at the excitation wavelength likely attenuates the incident light reaching the CP particles, consistent with a predominant primary inner filter effect, with possible additional contribution from surface-accessible CP particles. These results demonstrate preservation of the functional properties of the CPs within the composites and validate their applicability as solid-state sensing devices.

## Conclusions

In this work, we have demonstrated that rational control over particle size and morphology in 1D and 2D Cu­(I)-iodide CPs is an effective strategy for tuning their photophysical and catalytic properties. By utilizing one-step room-temperature synthetic routes, combined with bottom-up (PVP-assisted) and top-down (ultrasound and grinding) approaches, we successfully fabricated nanofibers and nanosheets while preserving the structural integrity of the coordination frameworks. Our findings establish that structural dimensionality significantly influences electronic response; while the 1D architecture of **CP1** is highly sensitive to defect-induced perturbations, the 2D layered topology of **CP2** maintains robust electronic communication even after significant downsizing. The reduction in particle dimensions enhanced photocatalytic performance by increasing the effective surface area and the density of active sites, with **CP1-PVP/GR** and **CP2-PVP/GR/US** achieving complete degradation of methyl orange within only 3–5 min. Furthermore, stable aqueous suspensions of sonicated particles serve as efficient and selective luminescent sensors for tetracycline detection via quenching mechanisms, offering competitive apparent *K*_*SV*_ and low detection limits. Finally, the integration of these materials into 3D-printed resins and PLA films validates their excellent processability and potential for developing portable, low-cost functional devices for environmental monitoring and remediation.

## Supplementary Material



## Notes

Description of the equipment and methodology used for characterization and processing; PXRD patterns, FTIR spectra, and SEM images of compounds; fluorescence and diffuse reflectance spectra; Tauc plots; density of states; ^1^H NMR spectra; reusability and cycling stability of compounds for MO degradation; FTIR and PXRD data after several cycles of MO degradation; emission spectra of compounds in the presence of TC; Stern–Volmer plots; FTIR and PXRD data of compounds after several cycles acting as TC sensors; interference plots with other antibiotics; energy values of the conduction band and valence band edges of **CP1** and **CP2** and the HOMO–LUMO values of TC; images of the devices obtained by drop casting and 3D printing; FTIR and PXRD data of the composites; SEM-EDX images; and images of the devices obtained under ultraviolet light in water and in the presence of CT (PDF)

## Notes

This work is dedicated to Juan Jesus Amo Ochoa, Marta Amo Ochoa, and Javier de la Fuente.
