# Supplementary material for: Engineering Morphology and Dimensionality in 1D and 2D Cu(I)‑I Coordination Polymers for Enhanced Photocatalysis, Luminescence, and Selective Antibiotic Sensing
Source: ACS Omega. 2026 Sep 16;11(38):57507–20. doi: 10.1021/acsomega.6c06025 (PMC13628993; doi:10.1021/acsomega.6c06025)
Supplement: Supplementary file 1 [file ao6c06025_si_001.pdf]

Supporting Information

Engineering Morphology and Dimensionality in 1D and 2D Cu(I)–I Coordination Polymers for Enhanced Photocatalysis, Luminescence, and Selective Antibiotic Sensing

*Julian Avila-Duran[α], Carlos Tabernero-Sestines[α], Fernando Aguilar-Galindo[β, γ], Pilar Amo-Ochoa[α, γ *]*

[α] *Inorganic Chemistry Department, Faculty of Sciences, Autonomous University of Madrid (UAM), 28049 Madrid, Spain.*

[β] *Chemistry Department, Faculty of Sciences, Autonomous University of Madrid (UAM), 28049 Madrid, Spain.*

[γ] *Institute for Advanced Research in Chemical Sciences (IAdChem). Autonomous University of Madrid (UAM), 28049 Madrid, Spain*

e-mail: pilar.amo@uam.es

Methods and equipment's

Infrared (FTIR-ATR). The infrared spectra were recorded on a PerkinElmer spectrophotometer equipped with a MIRacle Universal Attenuated Total Reflectance accessory (ATR).

Elemental Analysis (EA) measurements were carried out using a LECO CHNS-93217 elemental analyzer.

Powder X-ray Diffraction (PXRD) data were collected on a Rigaku Miniflex 600 HyPix-400 MF 2D together with 600W X-ray source diffractometer with geometry θ/2θ (Cu-Kα radiation; λ = 1.5418 Å). The samples were scanned over a range of 3° to 50° (θ/2θ), using an angular increment of 0.03° and a counting time of 10 °%per minute.

Diffuse Reflectance (DRS). A Varian Cary 500 spectrophotometer outfitted with an integrating sphere accessory was used to record the diffuse reflectance spectra. The reflectance data were then converted to an equivalent absorbance unit using the Kubelka–Munk function.[1, 2] This approach provides the foundation for employing the Tauc relationship,[3] which is commonly used to evaluate optical band gaps.

Scanning Electron Microscopy (SEM). Images were obtained using a Hitachi S-3000N SEM-EDX microscope, operating at an accelerating voltage of 5.0%kV and a chamber pressure of 10^{-9}%Pa. Before imaging, samples were coated with a thin chromium layer under a pressure of 10^{-3}%Pa. For the conglomerated nanoparticles, 1 mg of CP was suspended in 10 mL of ethanol

and sonicated at 80% power for 5 min to disaggregate the particles. Subsequently, 20 µL of the resulting suspension was deposited on a glass slide (0.5 × 0.5 cm) and, after air drying, the corresponding image was obtained.

Atomic force microscopy (AFM). Measurements were taken with an Agilent 5500 microscope using Cervantes Full Mode. The software used for image and data acquisition was Nanotec Electronica SL. WSxM. Samples were prepared by depositing 20 µL of a solution of 1 mg of CP in 10 mL of ethanol on a silicon oxide surface (1.5 x 1.5 cm). It is left to stand in air for 20 min and the supernatant is removed with an Ar flow.

Emission measurements emission spectra were collected for both solid and aqueous suspensions at room temperature using an FS5 spectrofluorometer. A xenon arc lamp provided UV excitation, and both emission and excitation spectra were recorded via Czerny-Turner monochromators equipped with a double grating turret and flat gratings, which ensured precise focusing over the entire wavelength range and minimized stray light. Tetracycline (TC) detection measurements were carried out employing a quartz cuvette.

Sonication (US) was performed in an Elma Transsonic Digital S ultrasonic bath, operated at 24%˚C and 80%% power. Centrifugation steps were conducted using an MPW-350R centrifuge.

Efficiency studies of the CPs in the **degradation studies** of the different organic dyes were evaluated by tracking the reduction in absorbance using an Agilent 8453 ultraviolet-visible (UV-Vis) spectrophotometer, recording spectra from 300%nm to 800%nm (λ).

The **photocatalytic performance** was investigated at 20%˚C under illumination from a 15%W purple LED installed in a photoreactor. The LED emission spectrum (λ%=%300–600%nm, integration time CR2-AP%+%200%ms, intensity 20.5674%W%m^{-2}) was recorded using a Stellarnet Blue-Wave UVNB50 spectroradiometer. To carry out the tests, 2%mg of each compound were placed in a vial containing 2%mL of a 3 × 10^{-5}%M dye solution. A separate vial with 2%mL of the same 3 × 10^{-5}%M dye served as a blank. Both the sample and the blank were placed in the photoreactor, equipped with a stirring system. After allowing a moderate amount of time to elapse, no changes in the blank are observed. The CPs are subsequently removed from the photoreactor, and the degradation of the dye is measured using UV-vis equipment.

The interaction of **CP-US** particles with tetracycline (TC) was evaluated in deionized water by measuring the variation in normalized emission intensity against analyte concentration. In the range 0-250 μM, the data were linearly fitted to the Stern-Volmer equation[4] ($\frac{I_0}{I_{corr}} = 1 + K_{SV}$ $[TC]$). During the construction of the calibration curve for the sensor by successive additions of TC, the fluorescence of the sensor was corrected for the dilution effect. For each point, a corrected intensity was calculated according to ($I_{corr} = I_{obs} \frac{(V_0+V_{ad})}{V_0}$).[5]

The **limit of detection (LOD)** was estimated following the IUPAC criterion[6] (LOD=$\frac{3\,\sigma}{m}$), where m is the slope of the Stern–Volmer calibration plot and σ is the standard deviation of the blank samples (I_0/I_{corr} in the absence of TC).

Proton Nuclear Magnetic Resonance (^{1}H-NMR) spectra were acquired at room temperature on a Bruker Avance III-HD Nanobay 300%MHz spectrometer (two-channel), fitted with a 5%mm

BBO ^{1}H/X probe and Z-gradient capability. For measurements in D_2O, 2%mg of each CP were combined with a 1500%ppm dye solution. ^{1}H-NMR tubes containing both CP and dye were compared at the initial time (t_0) and once complete degradation (t_f) was reached in the photoreactor.

3D printing. An ELEGOO Mars 2 Pro 3D printer was used. The software used for the models was Tinkercad. ELEGOO light-curing translucent resin (solidification wavelength = 405 nm, hardness = 84, viscosity = 150 - 200 mPa.s, liquid density = 1.100 g/cm3, solid density = 1.195 g/cm3, flexural strength = 59 - 70 MPa and tensile strength = 36 - 53 MPa) was used to generate the composites. And the solvent used to clean the composites is isopropanol. After 3D printing, the device was placed in an ELEGOO Mercury Plus curing station for 2 min, one minute on each side, to achieve post-curing to ensure complete solidification of the resin.

Theoretical calculations. Density Functional Theory (DFT) calculations were performed with the Vienna Ab initio Simulation Package (VASP),[7] including periodic boundary conditions. Geometry optimizations were performed with the OPTPBE[8] functional and, on the top of the minima of the potential energy surface, we obtained a more accurate description of the electronic structure through single point calculations with the HSE functional.[9] We expanded the electron density in a plane-wave basis with a kinetic cutoff of 400 eV. PAW pseudopotentials[10] were used, as downloaded from the VASP database. The convergence criterion for the self-consistent (electronic) energy and the Hellmann-Feynman forces were 10^{-5} eV and 0.01 eV/Å, respectively.

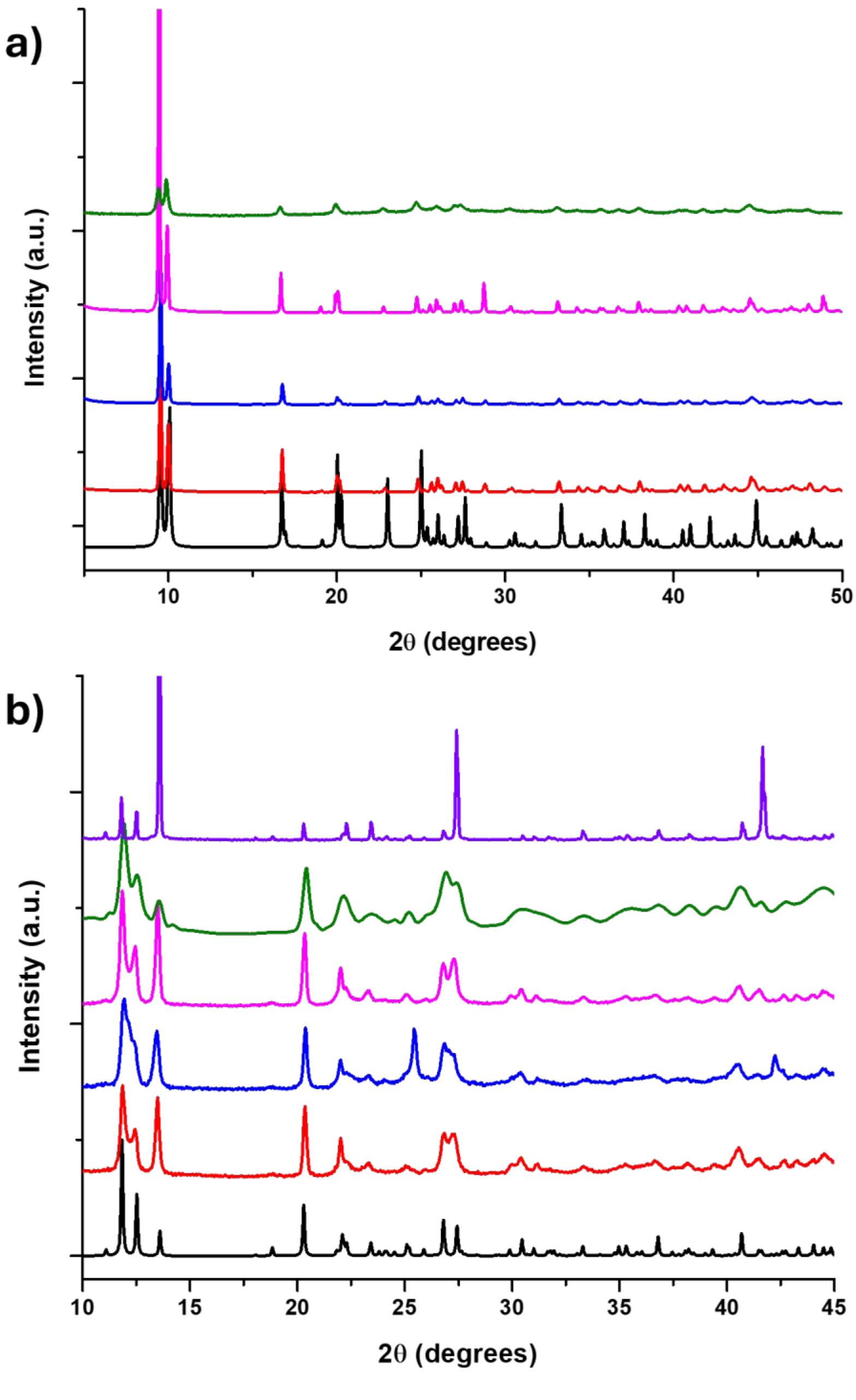

Figure S1. PXRD of theoretical (black line) and experimental **CP-RT** (red line), **CP-PVP** (blue line), **CP-US** (pink line), **CP-GR** (green line) and **CP2 ST** (violet line), of a) **CP1** and b) **CP2**.

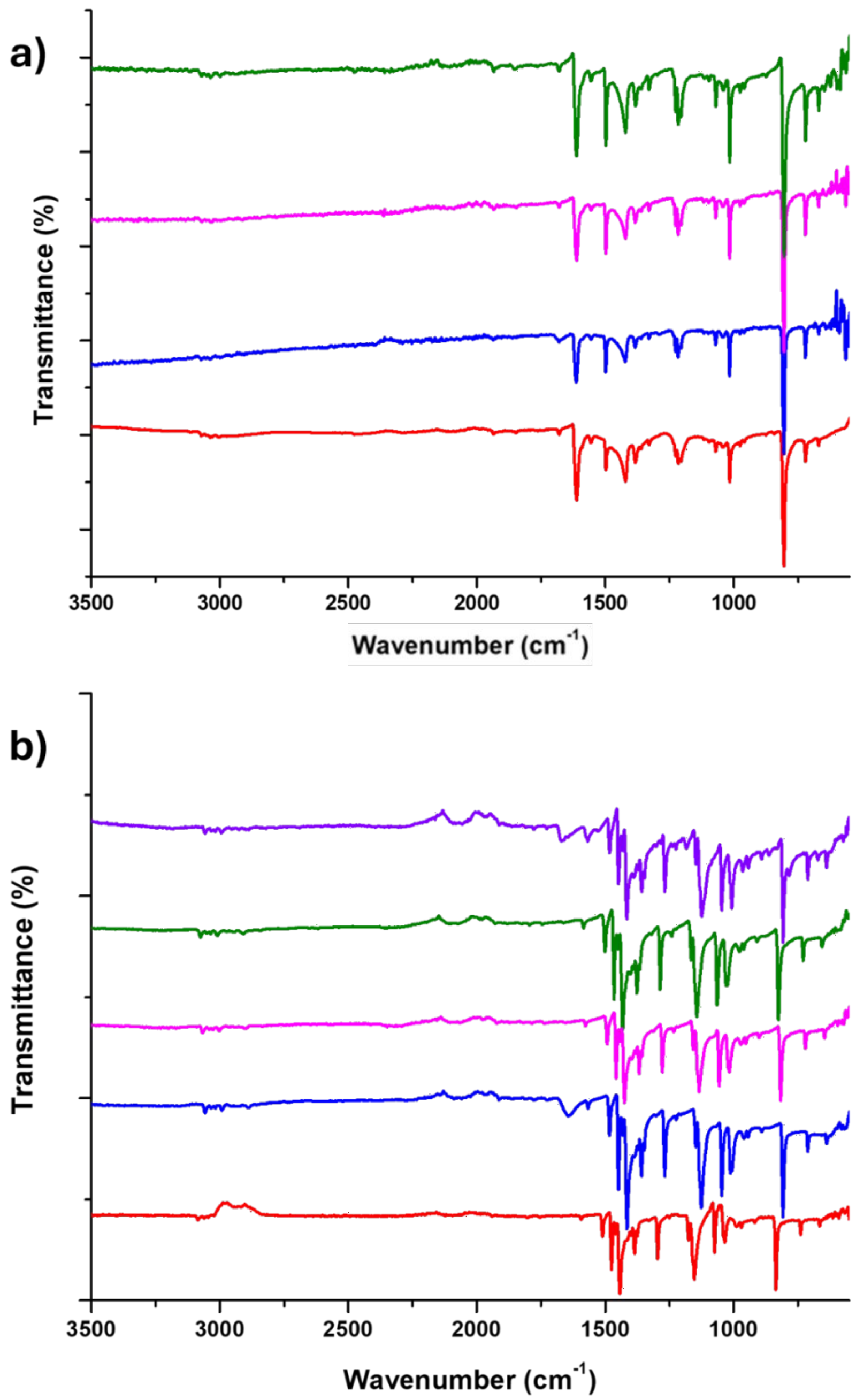

Figure S2. IR spectrum of experimental **CP-RT** (red line), **CP-PVP** (blue line), **CP-US** (pink line), **CP-GR** (green line) and **CP2-ST** (violet line), of a) **CP1** and b) **CP2**.

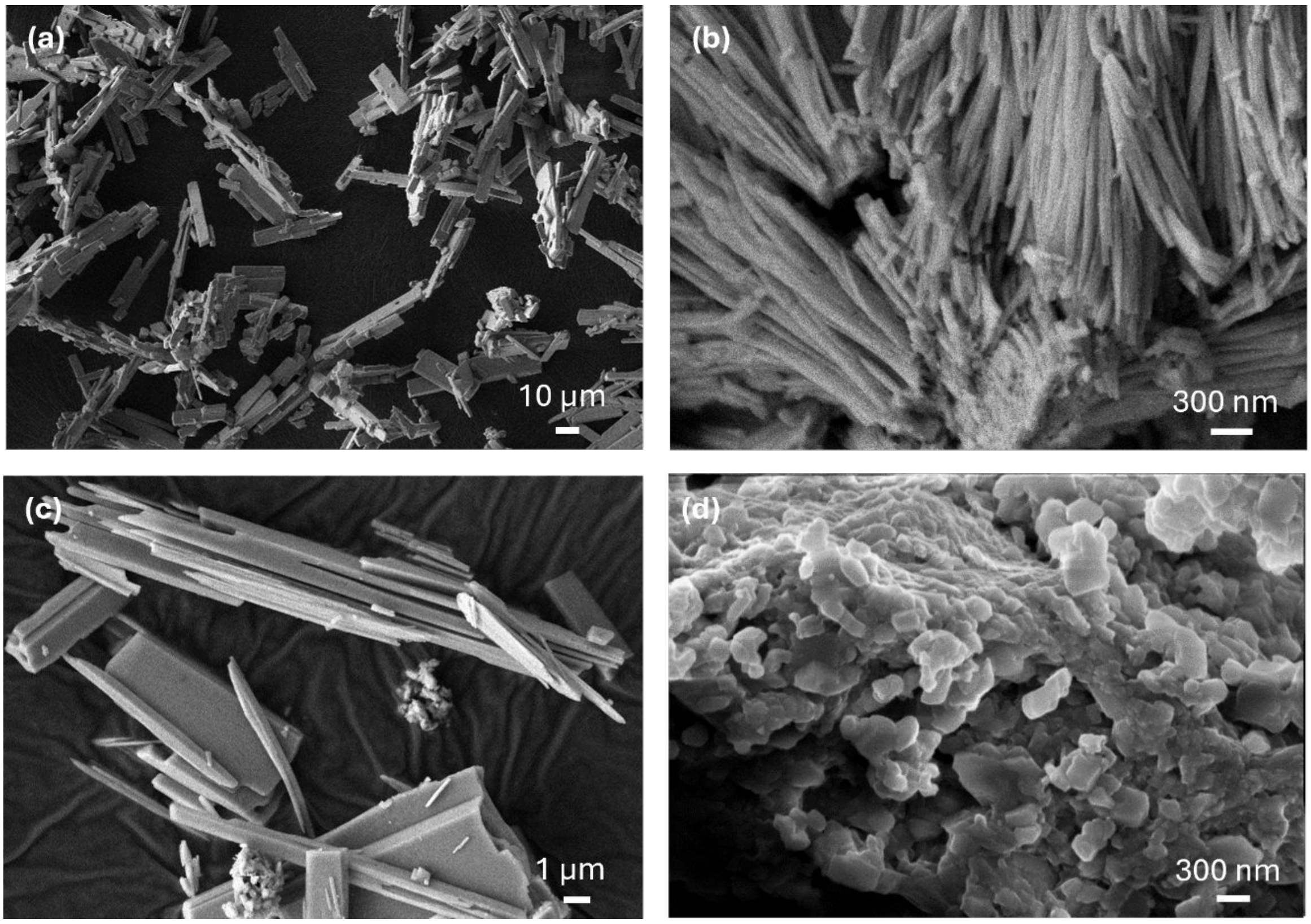

Figure S3. SEM images of **CP1-RT** (a), **CP1-PVP** (b), **CP1-US** (c) and **CP1-GR**(d).

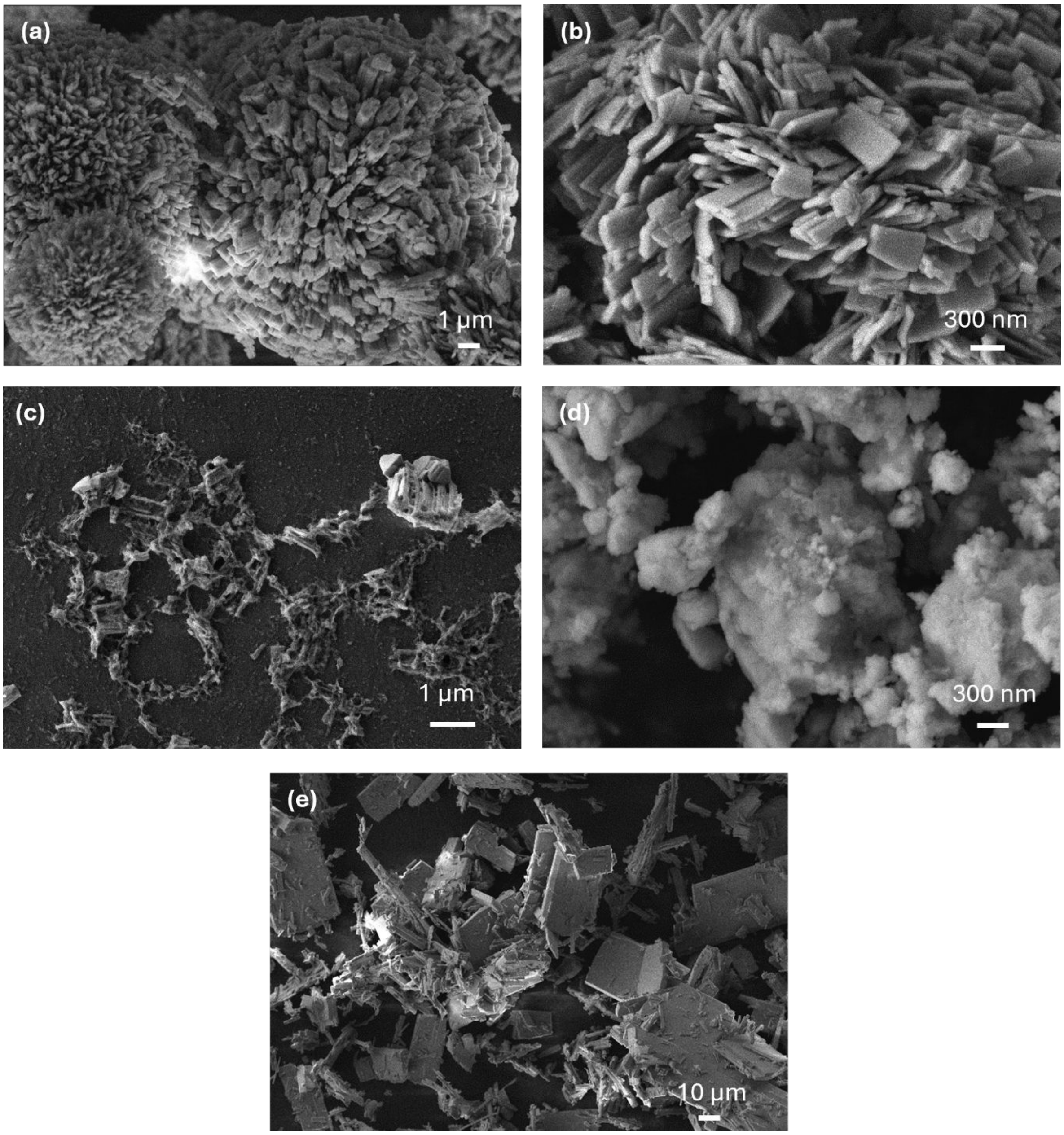

Figure S4. SEM images of **CP2-RT** (a), **CP2-PVP** (b), **CP2-US** (c), **CP2-GR** (d) and **CP2-ST** (e).

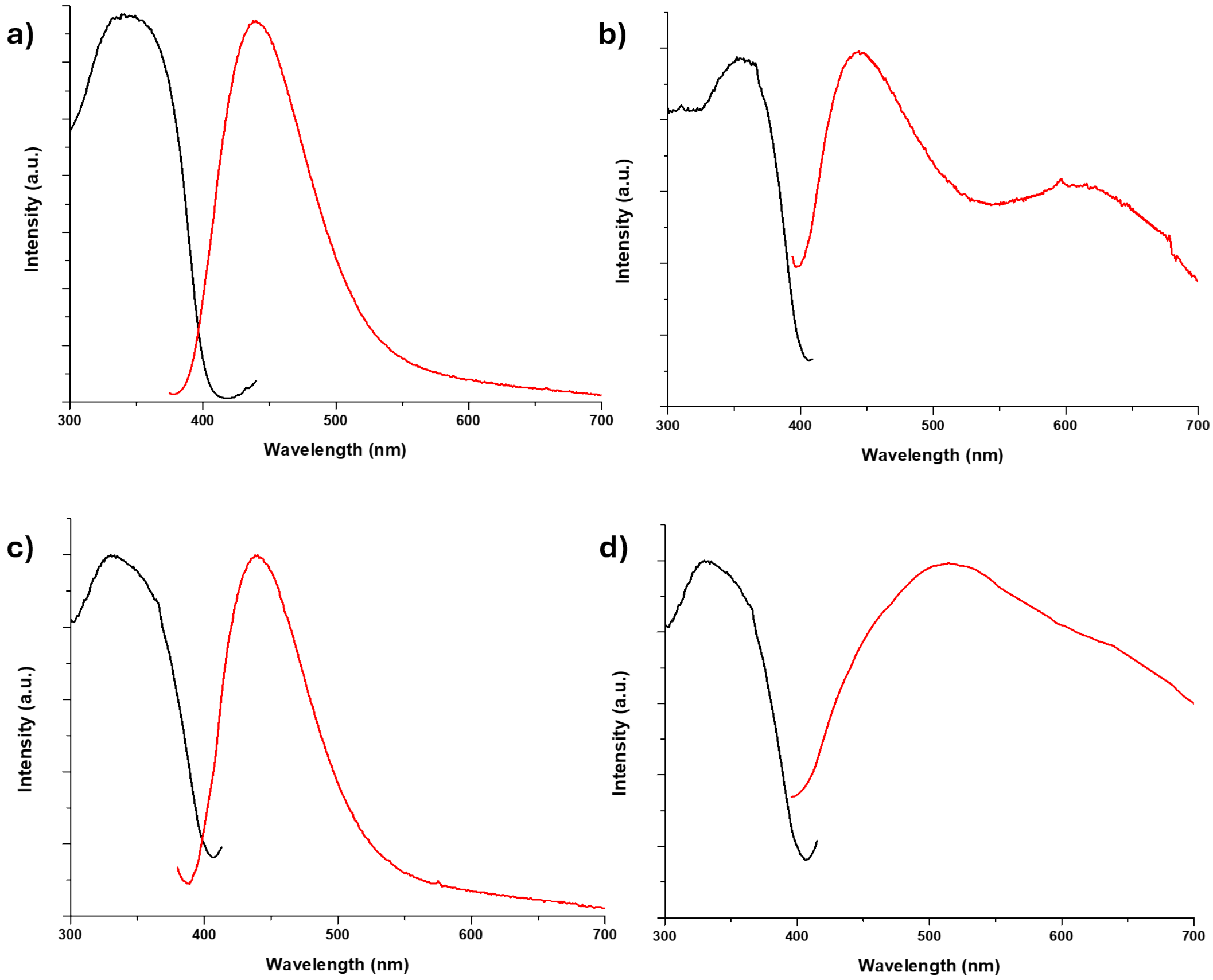

Figure S5. Fluorescence spectra in the 300-700 nm range of emission (red) and excitation (black) from a) **CP1-RT**, b) **CP1-PVP**, c) **CP1-US** and d) **CP1-GR**.

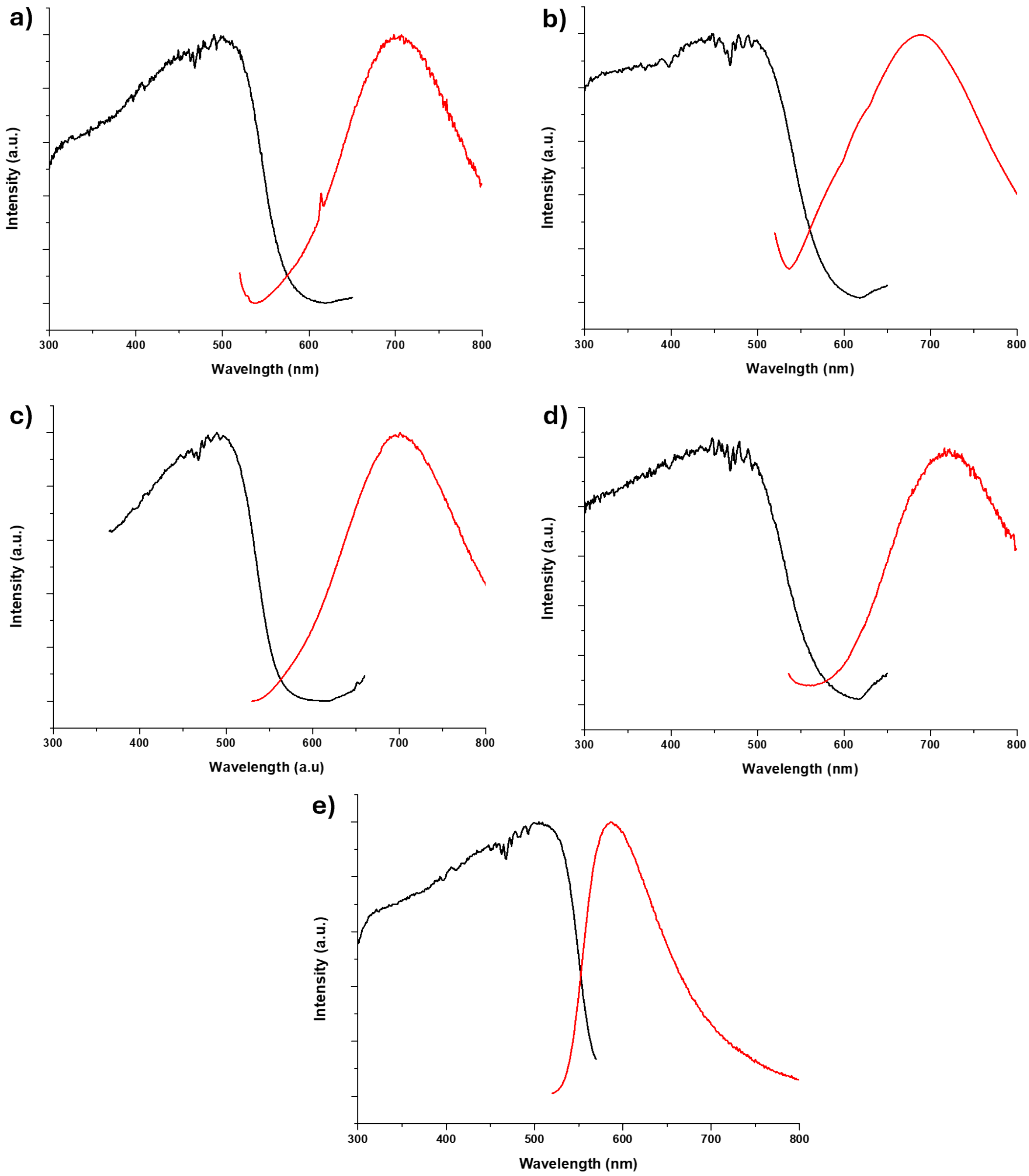

Figure S6. Fluorescence spectra in the 300-800 nm range of emission (red) and excitation (black) from a) **CP2-RT**, b) **CP2-PVP**, c) **CP2-US**, d) **CP2-GR** and e) **CP2-ST**.

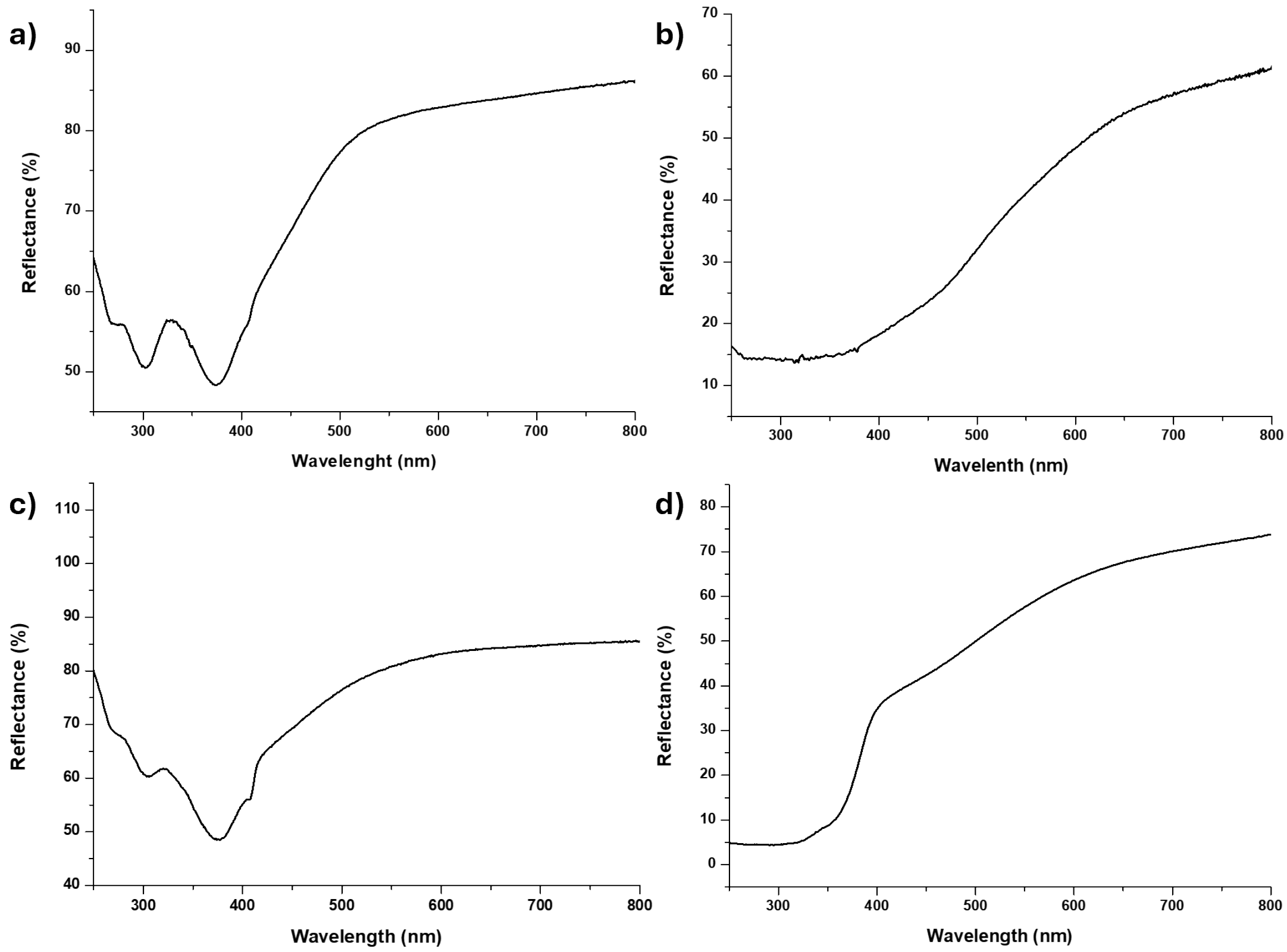

Figure S7. Experimental diffuse reflectance spectra of the a) **CP1-RT**, b) **CP1-PVP**, c) **CP1-US** and d) **CP1-GR**.

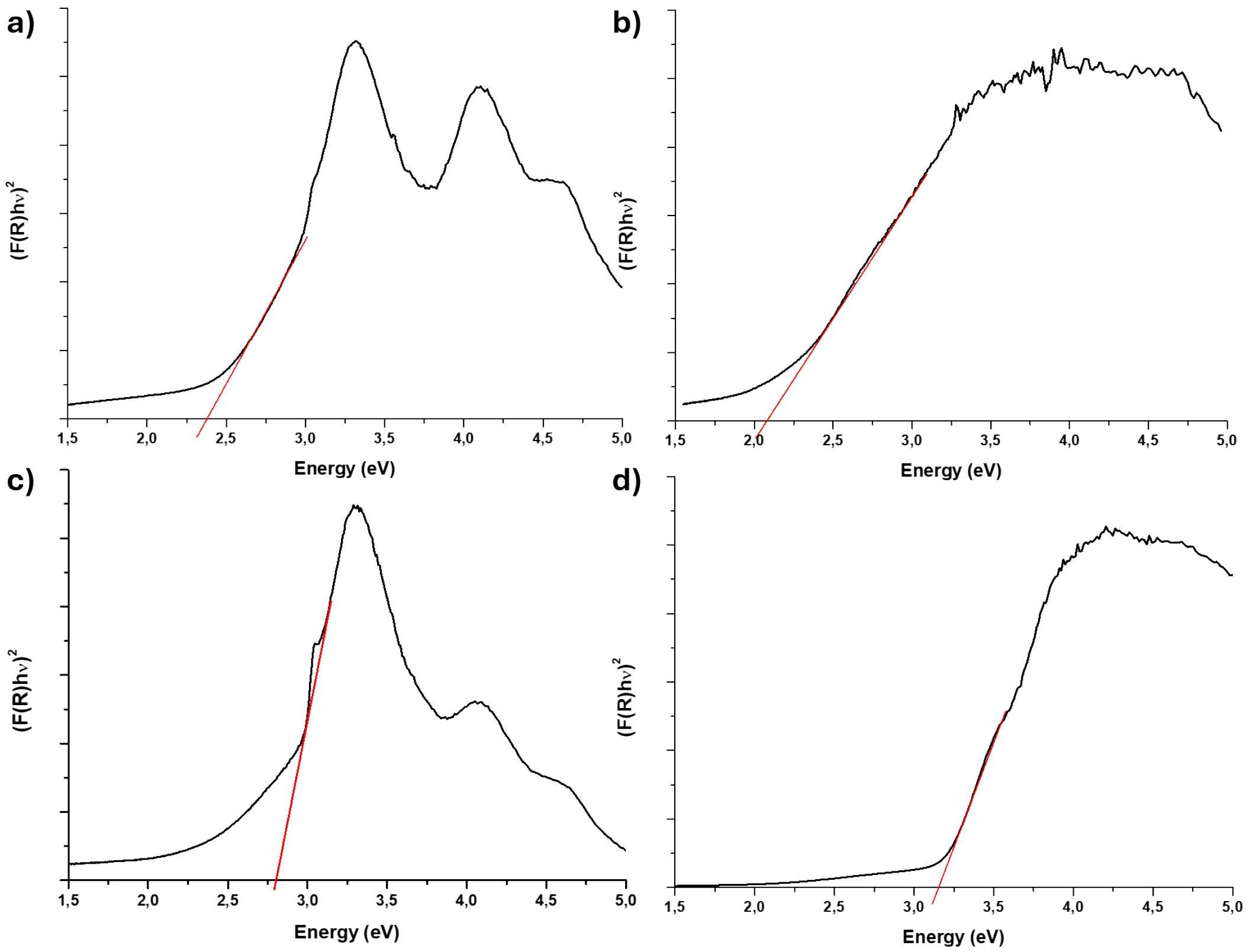

Figure S8. Tauc plots obtained with the Kubelka-Munk function and the linear fit for optical band gaps of the a) **CP1-RT**, b) **CP1-PVP**, c) **CP1-US** and d) **CP1-GR**.

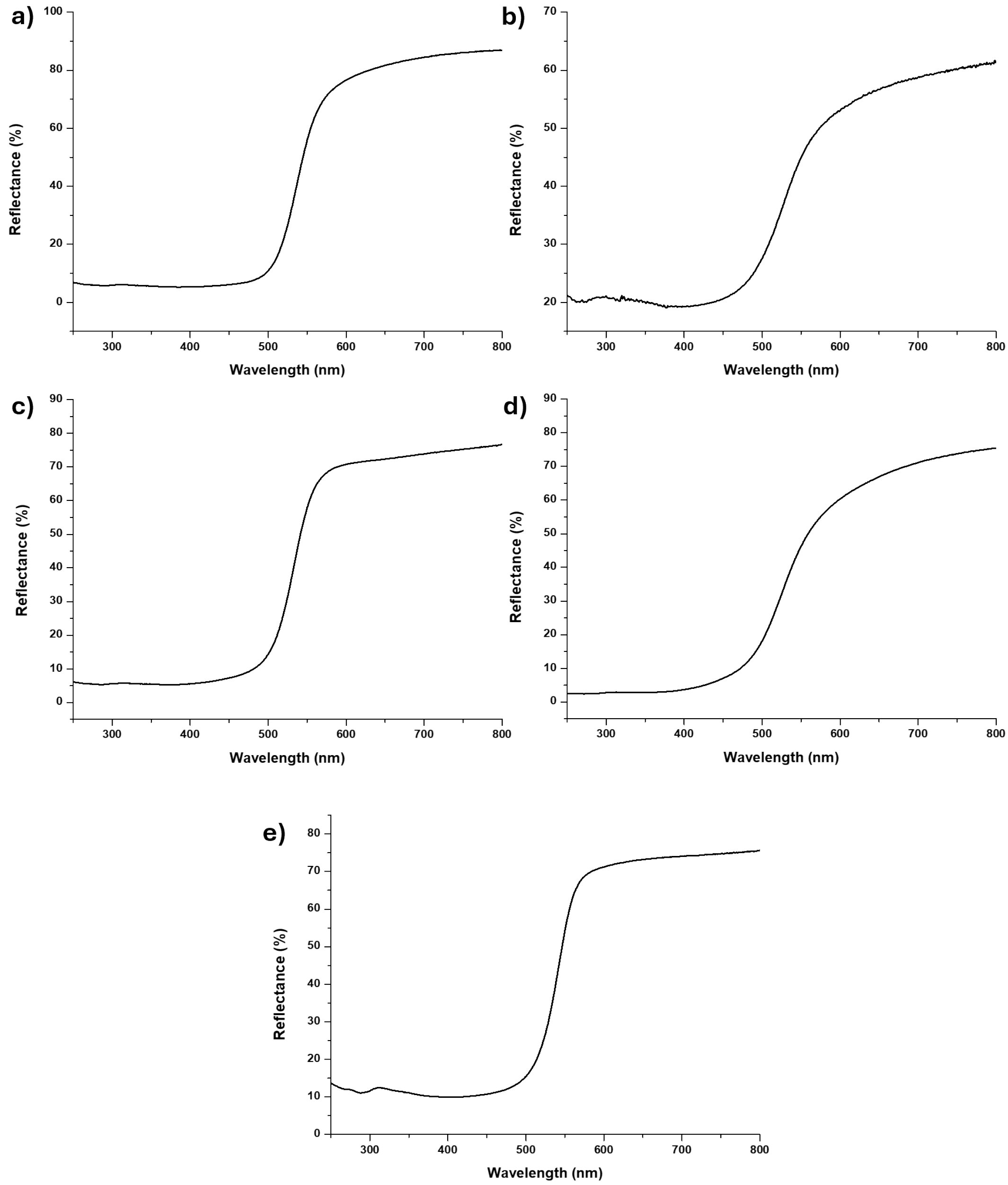

Figure S9. Experimental diffuse reflectance spectra of the a) **CP2-RT**, b) **CP2-PVP**, c) **CP2-US**, d) **CP2-GR** and e) **CP2-ST**.

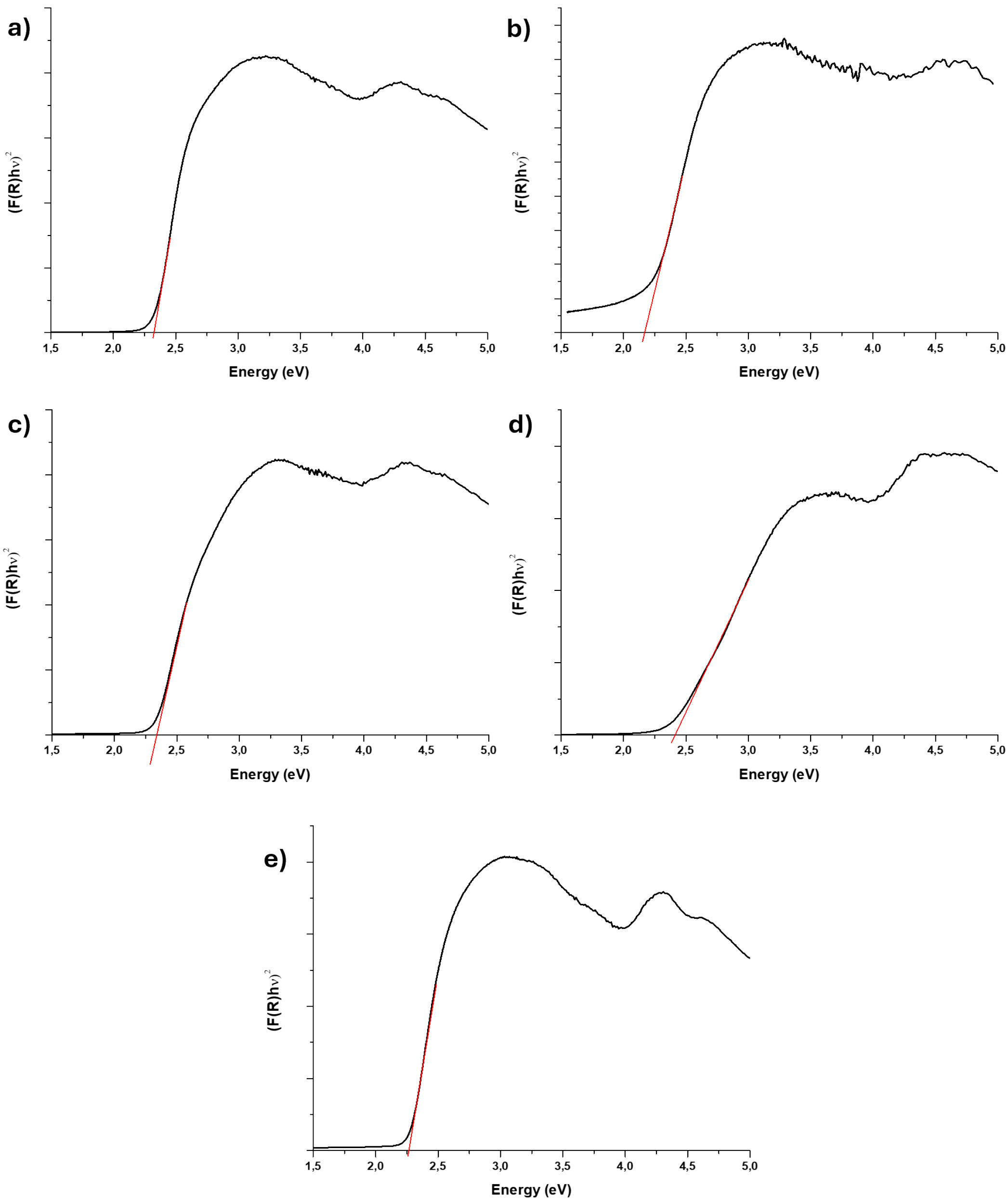

Figure S10. Tauc plots obtained with the Kubelka-Munk function and the linear fit for optical band gaps of the a) **CP2-RT**, b) **CP2-PVP**, c) **CP2-US**, d) **CP2-GR** and e) **CP2-ST**.

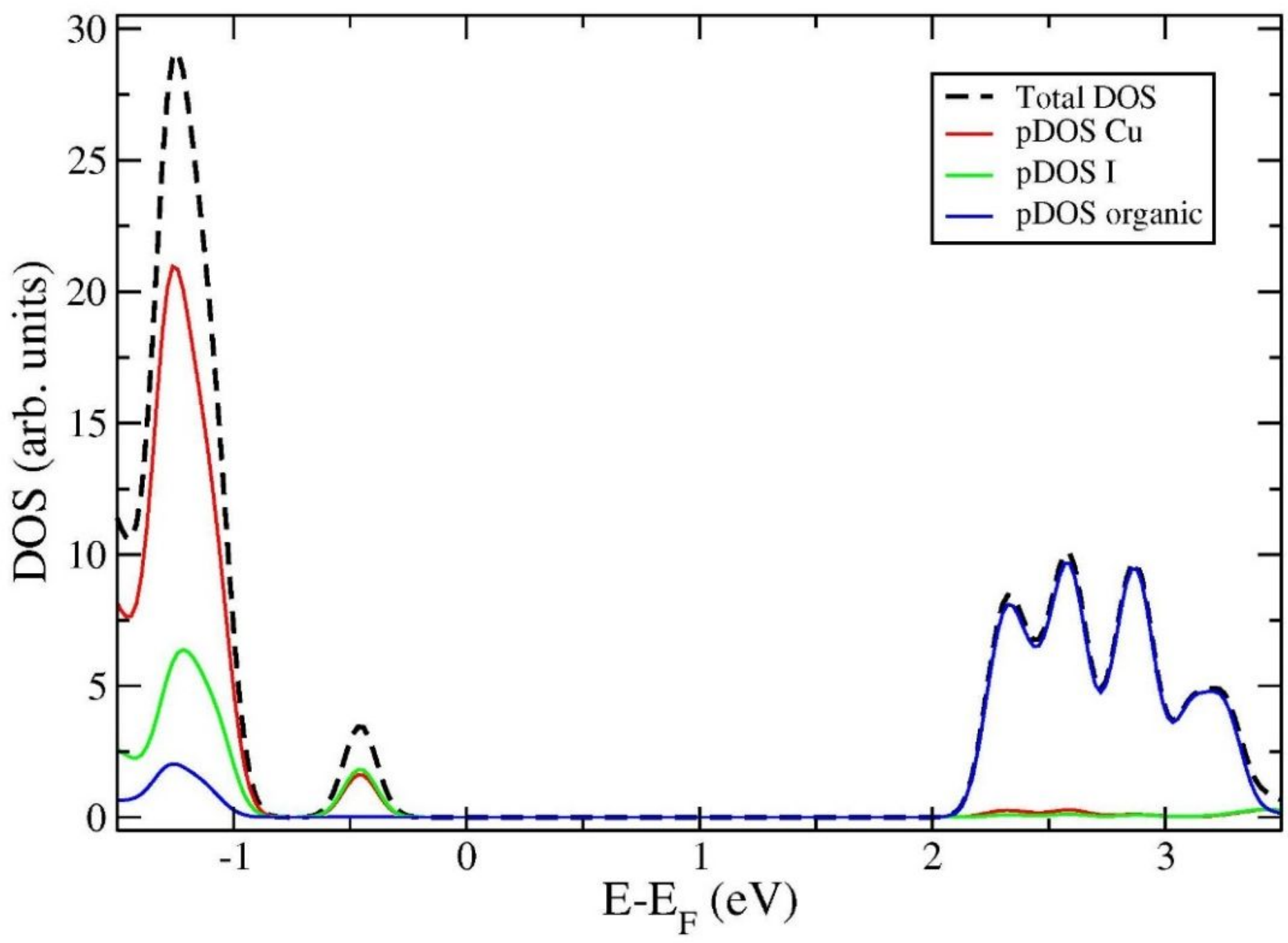

Figure S11. Density of states (DOS) of **CP1** with projection on the ligand atoms and on the Cu and I unit atoms.

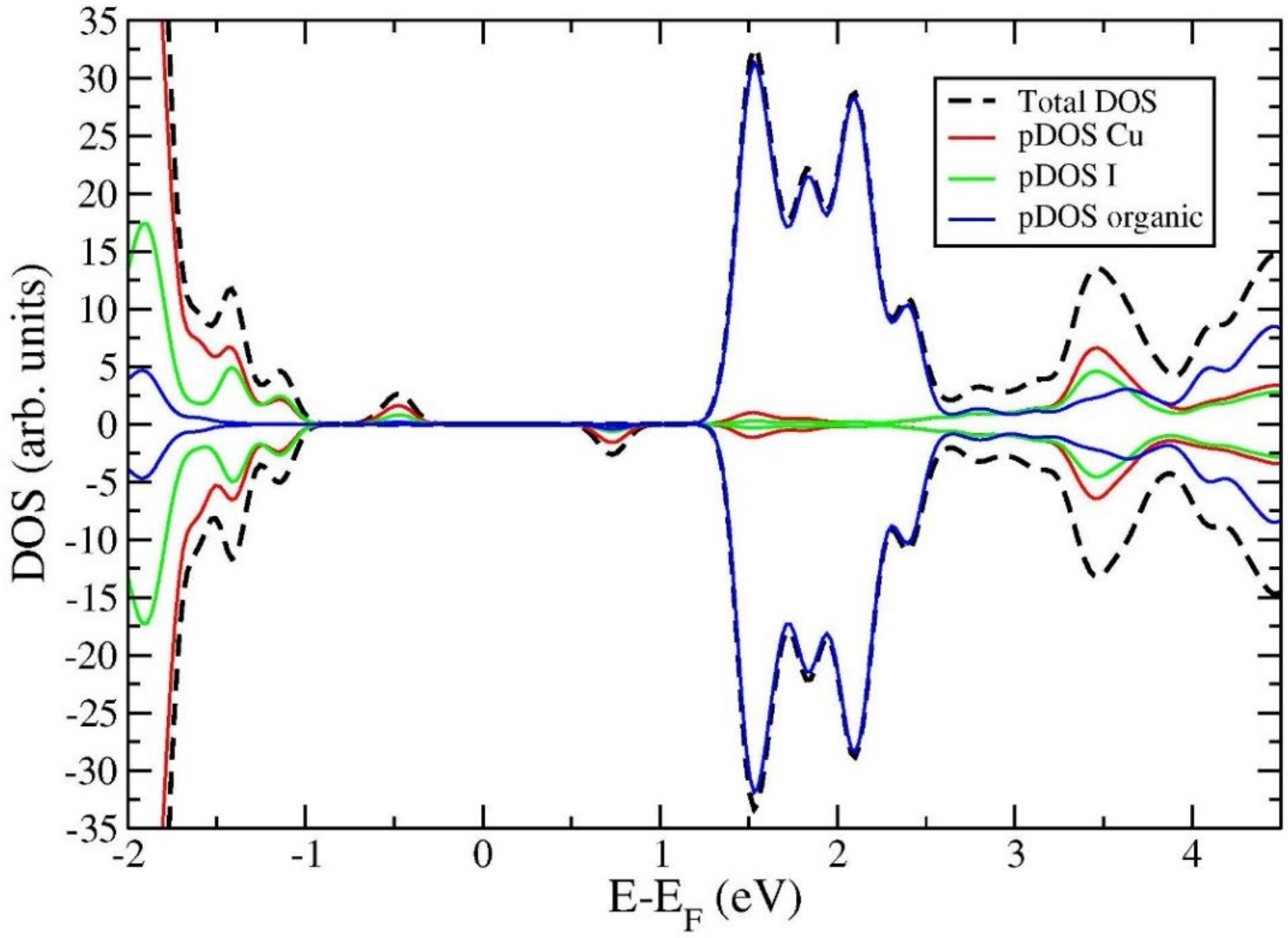

Figure S12. Density of states (DOS) of **CP1,** when one iodine atom is removed, with projection on the ligand atoms and on the Cu and I unit atoms.

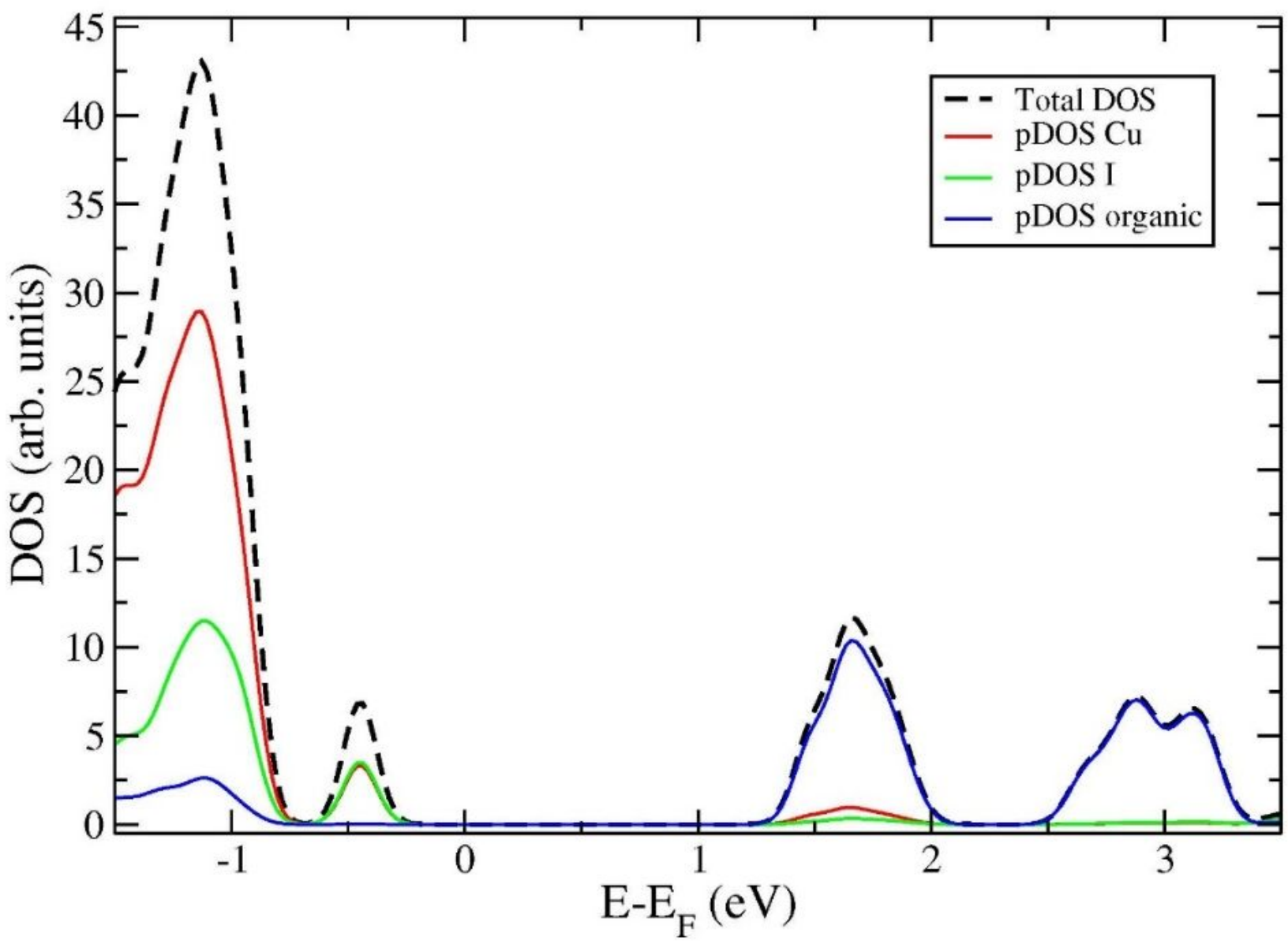

Figure S13. Density of states (DOS) of **CP2** with projection on the ligand atoms and on the Cu and I unit atoms.

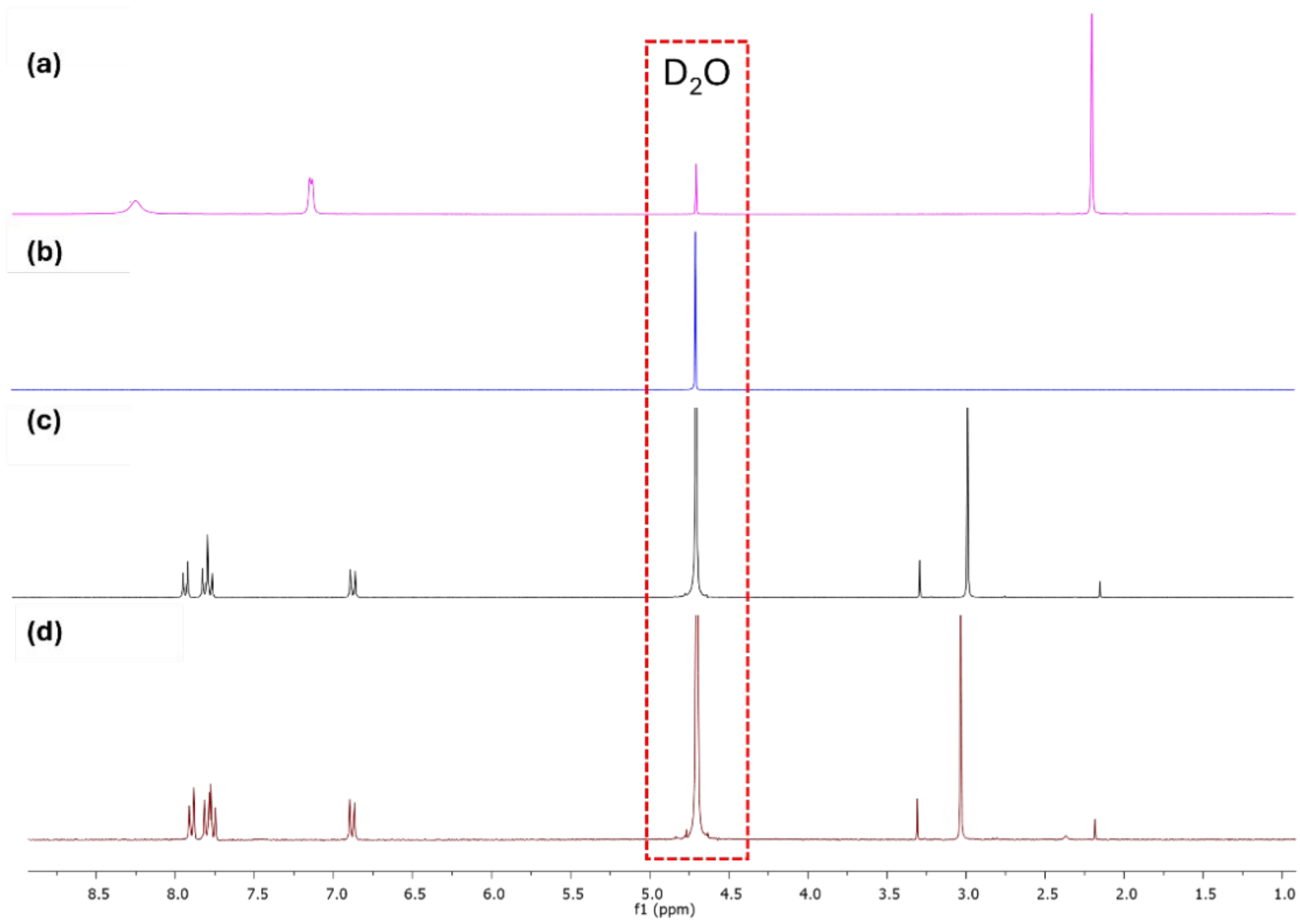

Figure S14. ^{1}H-NMR study of **CP1** stability in D_2O in the absence and presence of MO (10^{-3} M): 4-methylpyridine (a), **CP1** in water (90 min) (b), **CP1** + MO (t = 0 min.) (c) and **CP1** + MO (t = 90 min) (d) in D_2O respectively. The aromatic signals observed in (c) and (d)

correspond to MO, while no signals attributable to free 4-methylpyridine are detected after irradiation.

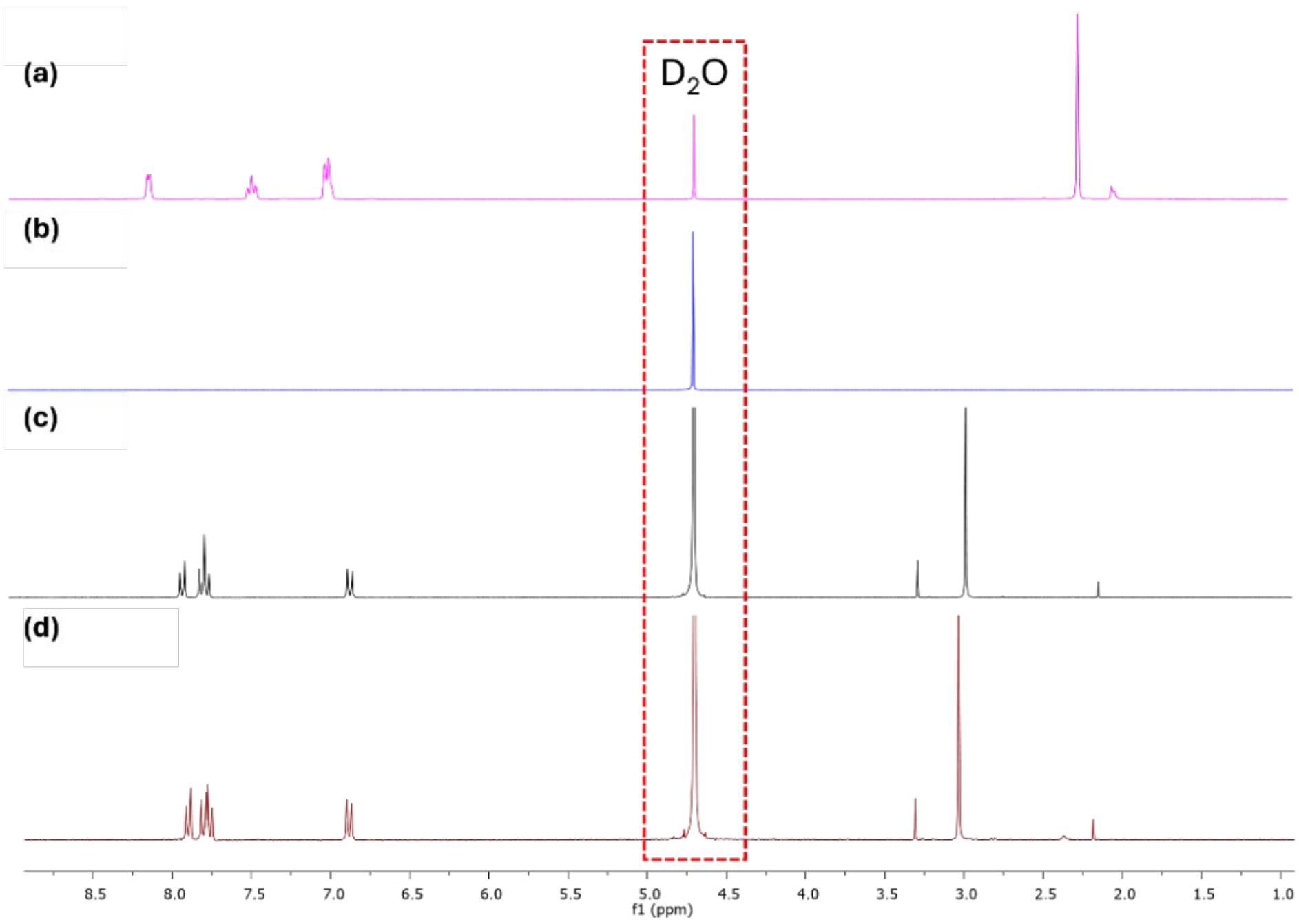

Figure S15. ^{1}H-NMR study of **CP2** stability in D_2O in the absence and presence of MO (10^{-3} M): 2-methylpyrazine (a), **CP2** in water (90 min) (b), **CP2** + MO (t = 0 min.) (c) and **CP2** + MO (t = 90 min) (d) in D_2O respectively. The aromatic signals observed in (c) and (d) correspond to MO, while no signals attributable to free 2-methylpyrazine are detected after irradiation.

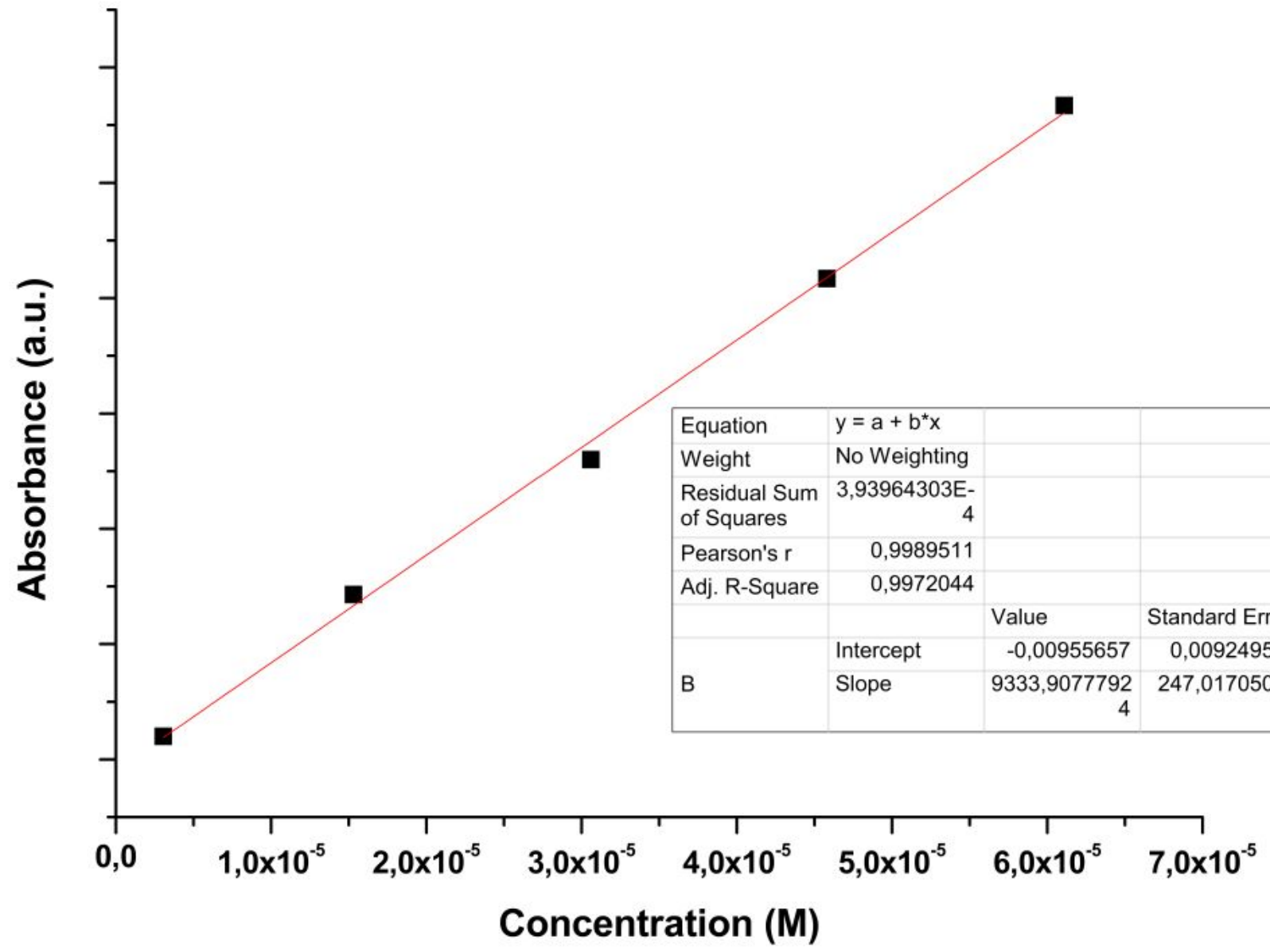

Figure S16. Methyl orange (MO) calibration curve.

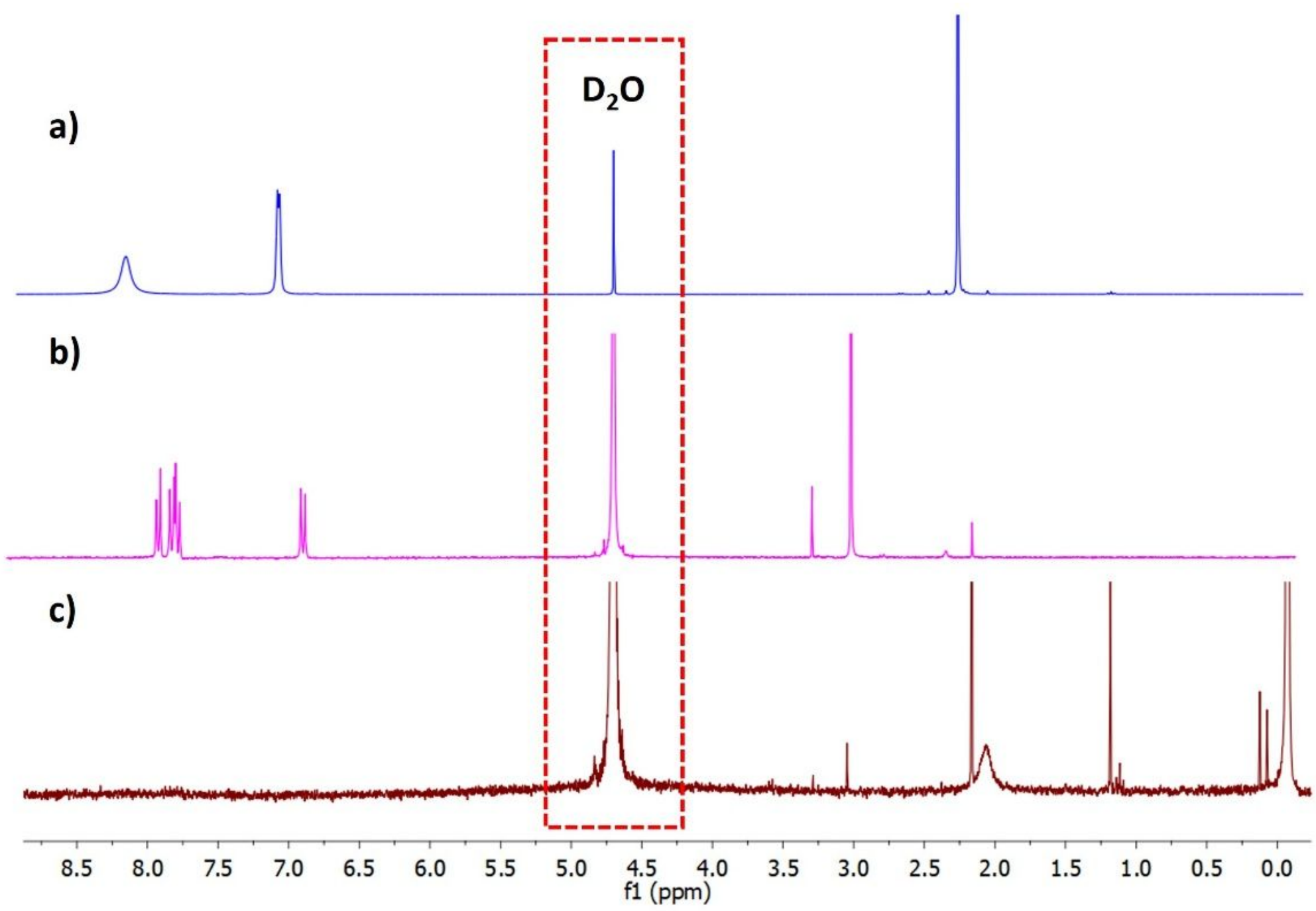

Figure S17. ^{1}H-NMR study of MO degradation by **CP1** under the photocatalytic conditions employed in the UV-Vis experiments (MO concentration: 10^{-5} M): 4-methylpyridine (a), MO (b), and the solution obtained after irradiation in the presence of **CP1** (c), recorded in D_2O.

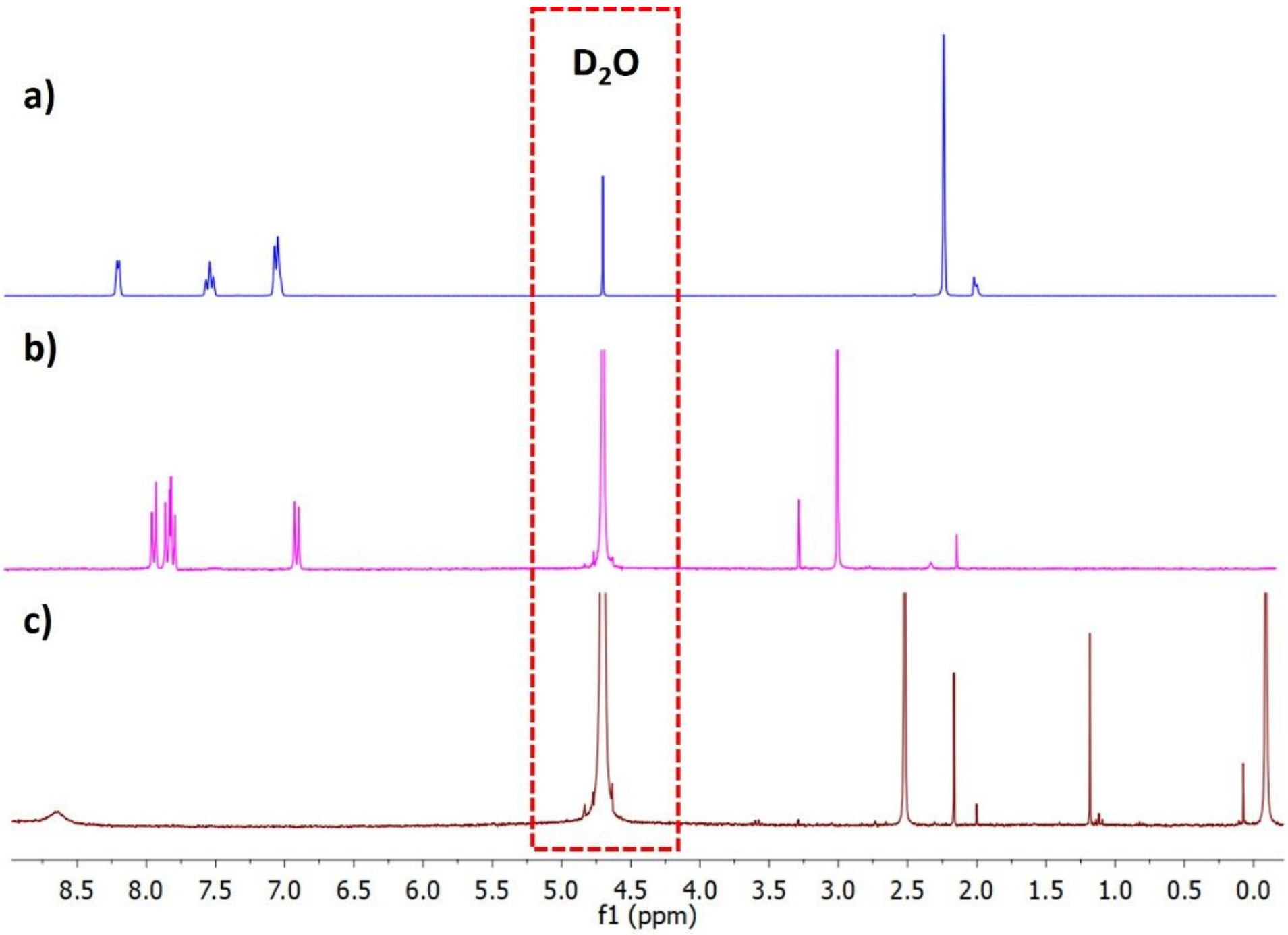

Figure S18. ^{1}H-NMR study of MO degradation by **CP2** under the photocatalytic conditions employed in the UV-Vis experiments (MO concentration: 10^{-5} M): 2-methylpyrazine (a), MO (b), and the solution obtained after irradiation in the presence of **CP2** (c), recorded in D_2O.

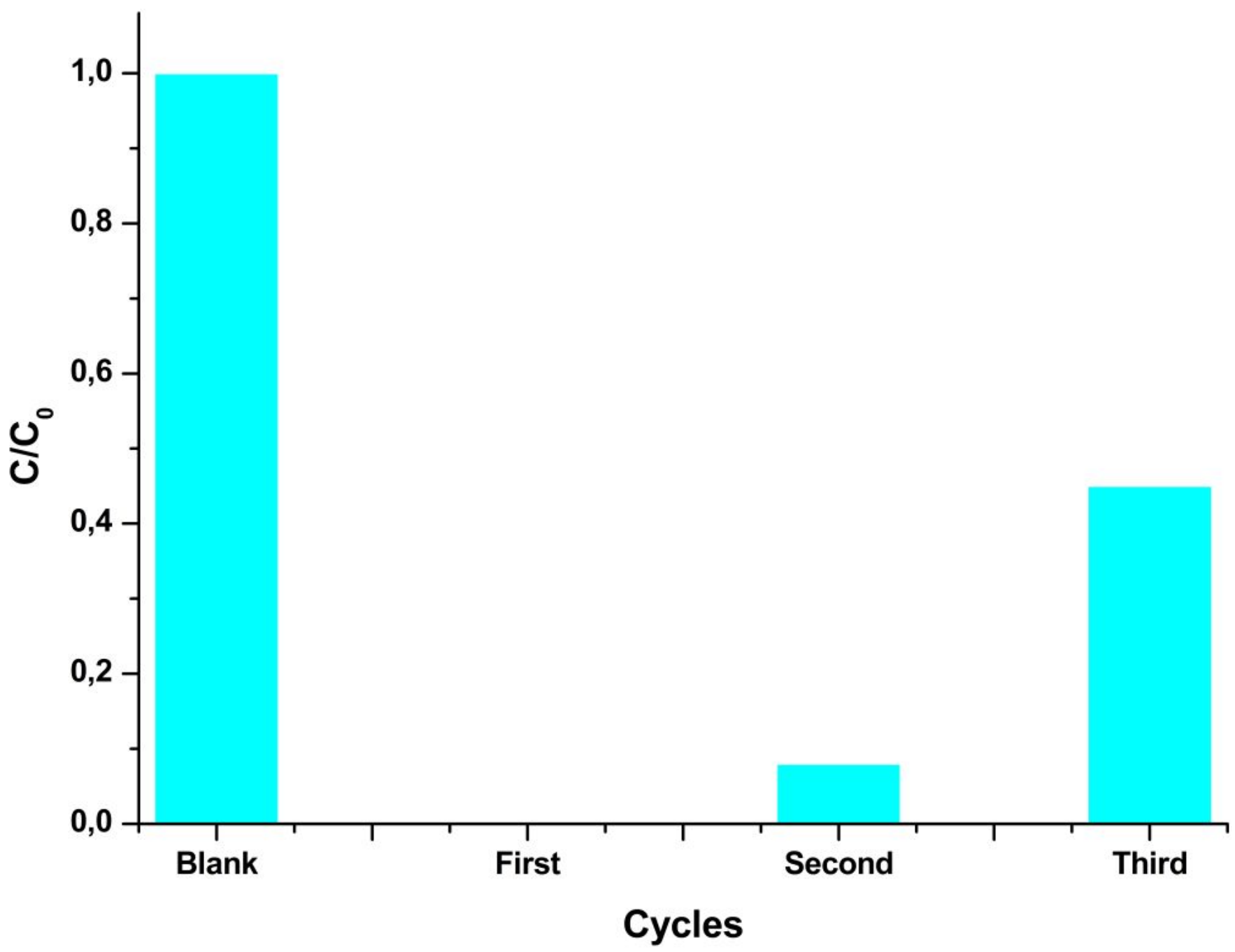

Figure S19. Reusability and cycling stability of the **CP1** for MO degradation.

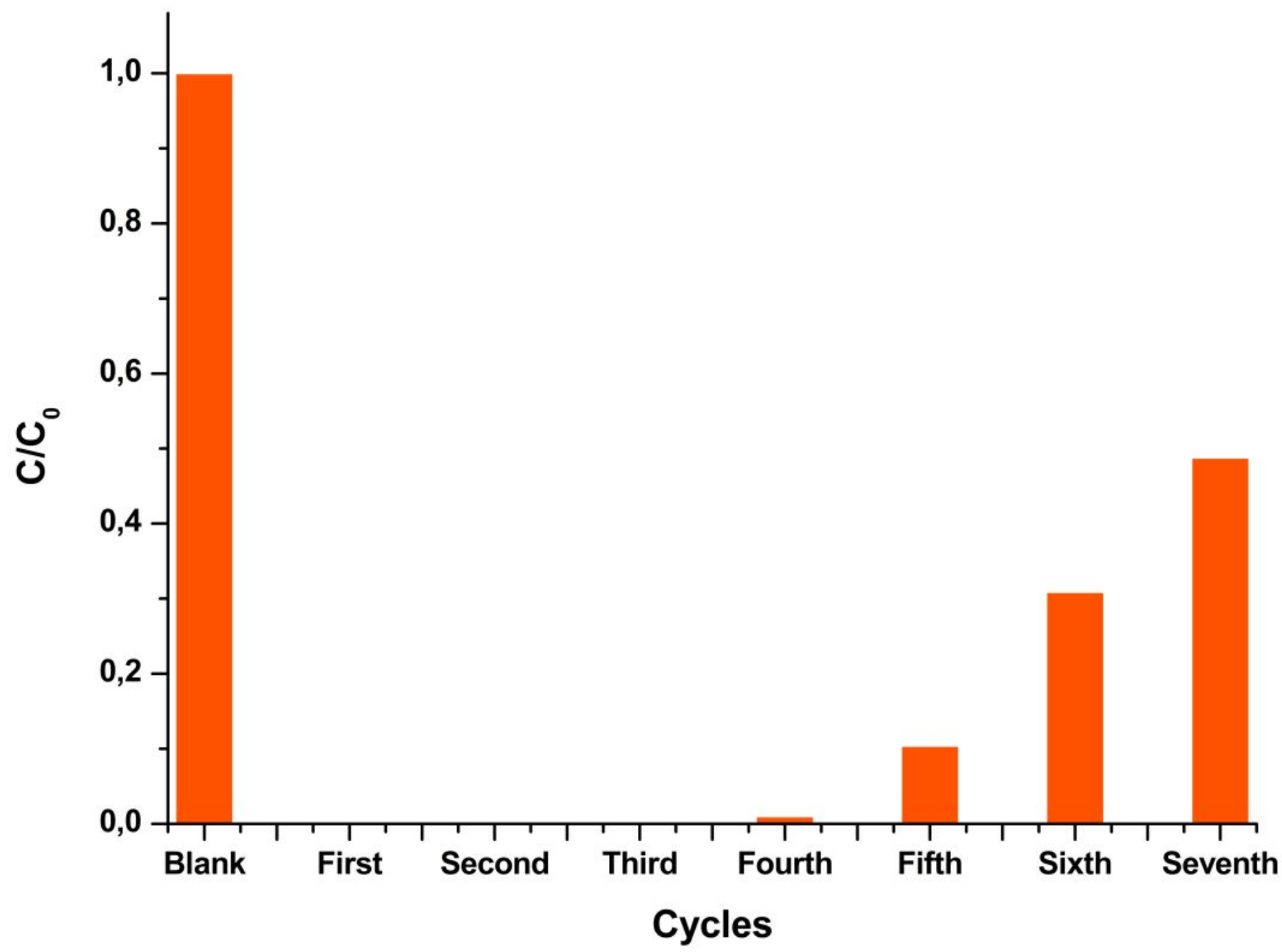

Figure S20. Reusability and cycling stability of the **CP2** for MO degradation.

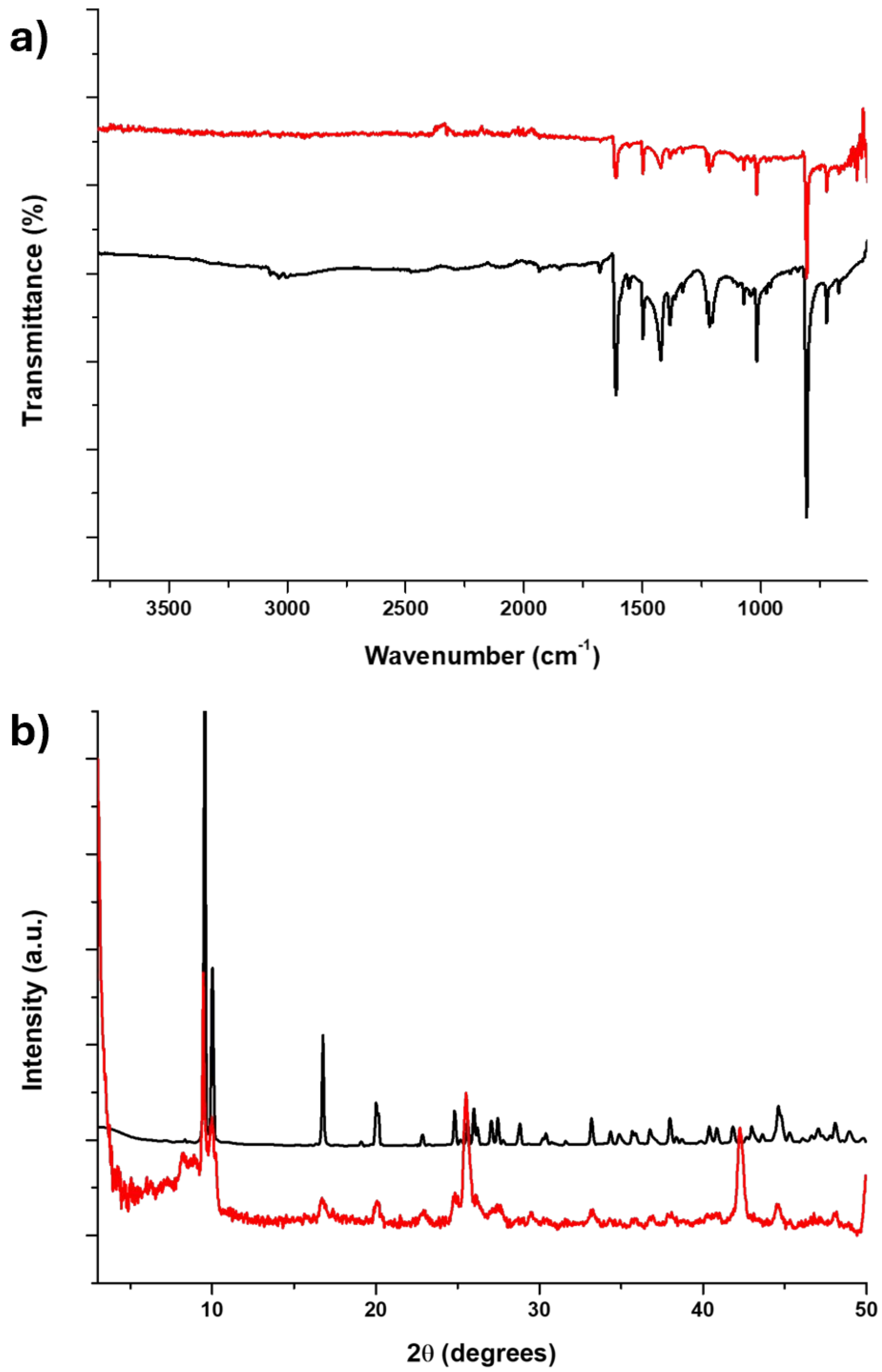

Figure S21. Characterization a) FTIR and b) PXRD of **CP1** after 3 cycles of MO degradation: **CP1-RT** before MO degradation (black line) and **CP1-RT** after MO degradation (red line).

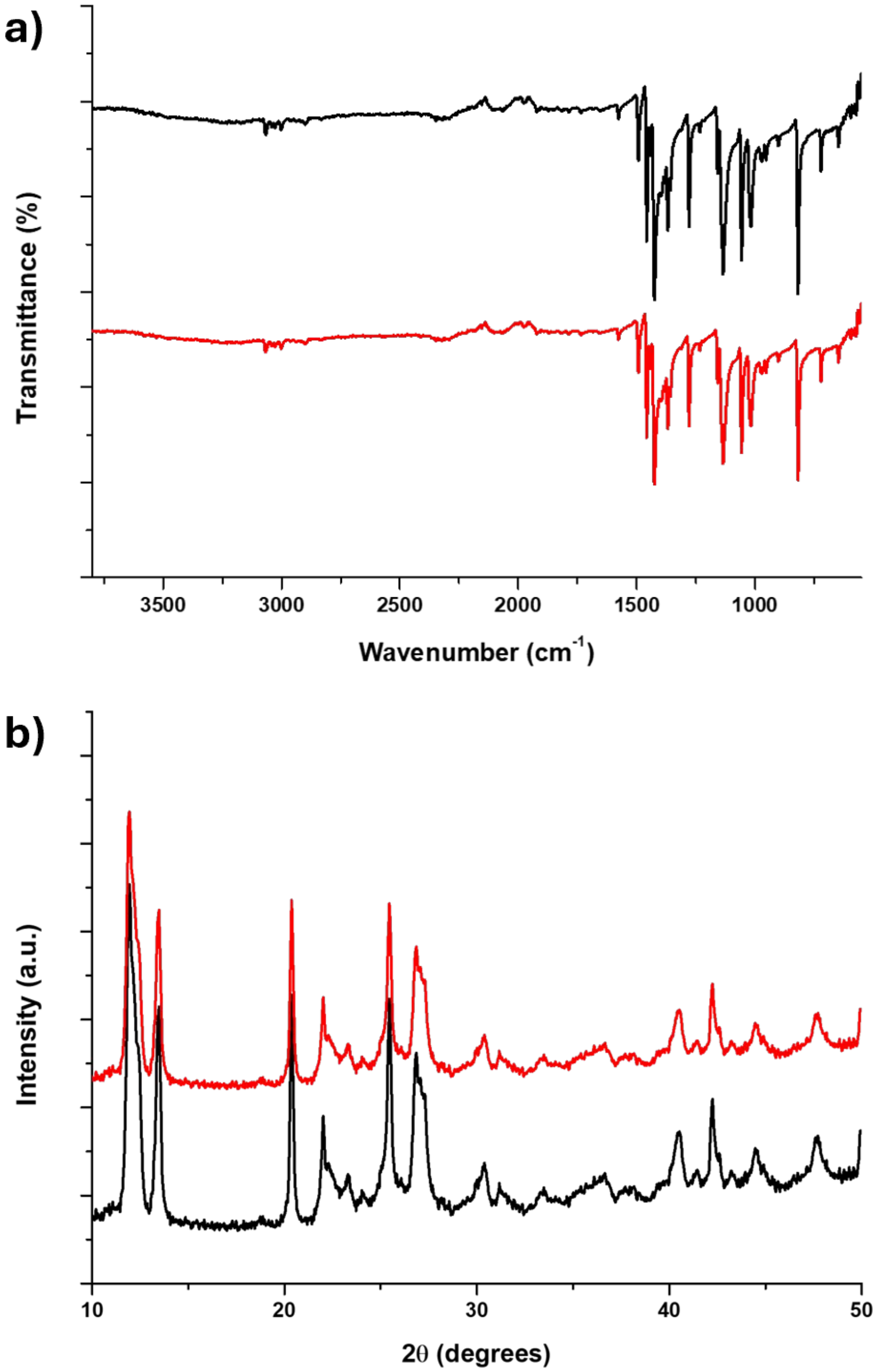

Figure S22. Characterization a) FTIR and b) PXRD of **CP2** after 7 cycles of MO degradation: **CP2-RT** before MO degradation (black line) and **CP2-RT** after MO degradation (red line).

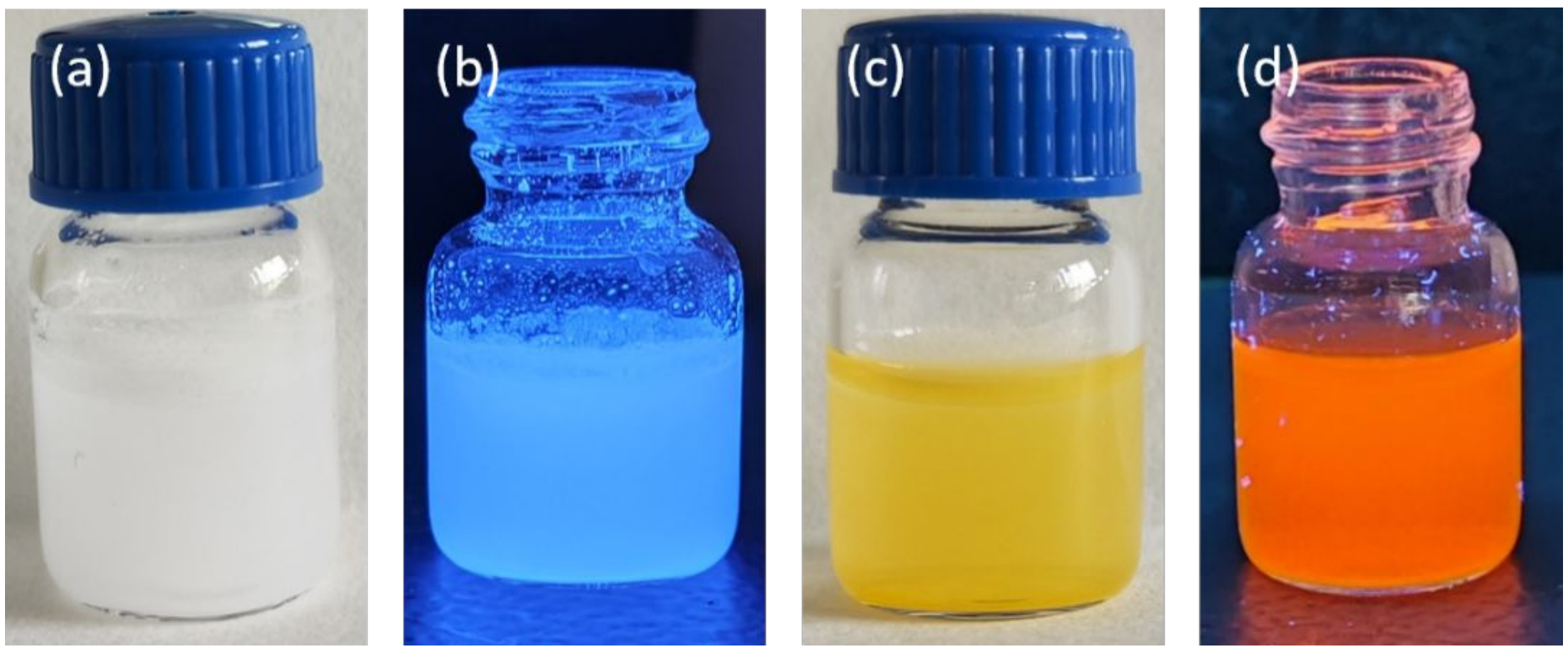

Figure S23. Stability of the suspensions in water 5 min after sonication under visible light for **CP1-US** (a) and **CP2-US** (c), and under UV light at 365 nm for **CP1-US** (b) and **CP2-US** (d).

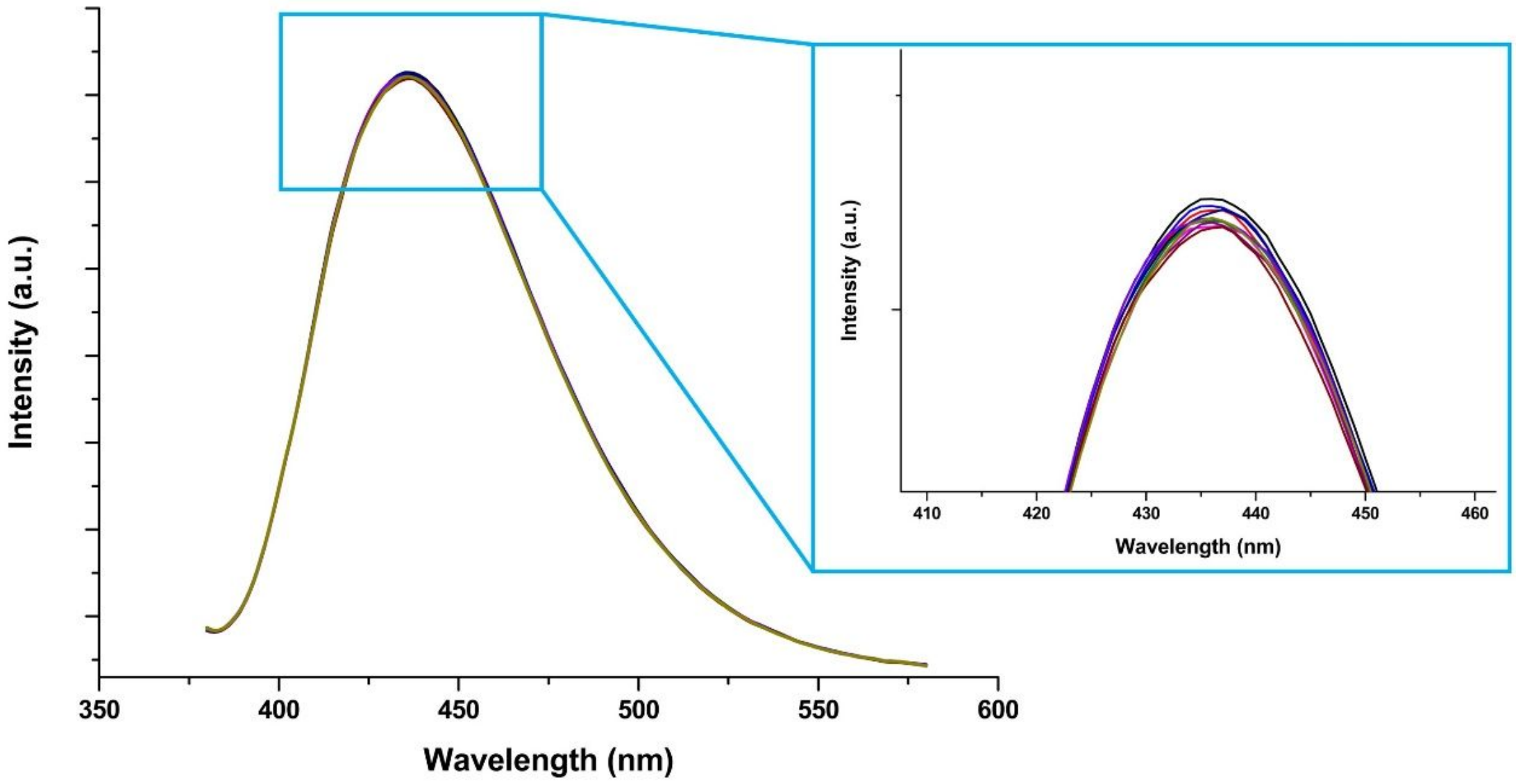

Figure S24. Blank emission measurements of **CP1-US** aqueous suspension in the absence of tetracycline. Experimental conditions: deionized water, room temperature, λ_{ex}= 365 nm, **CP1-US** concentration = 1 mg. mL^{-1}. The suspension was freshly vortexed before each measurement.

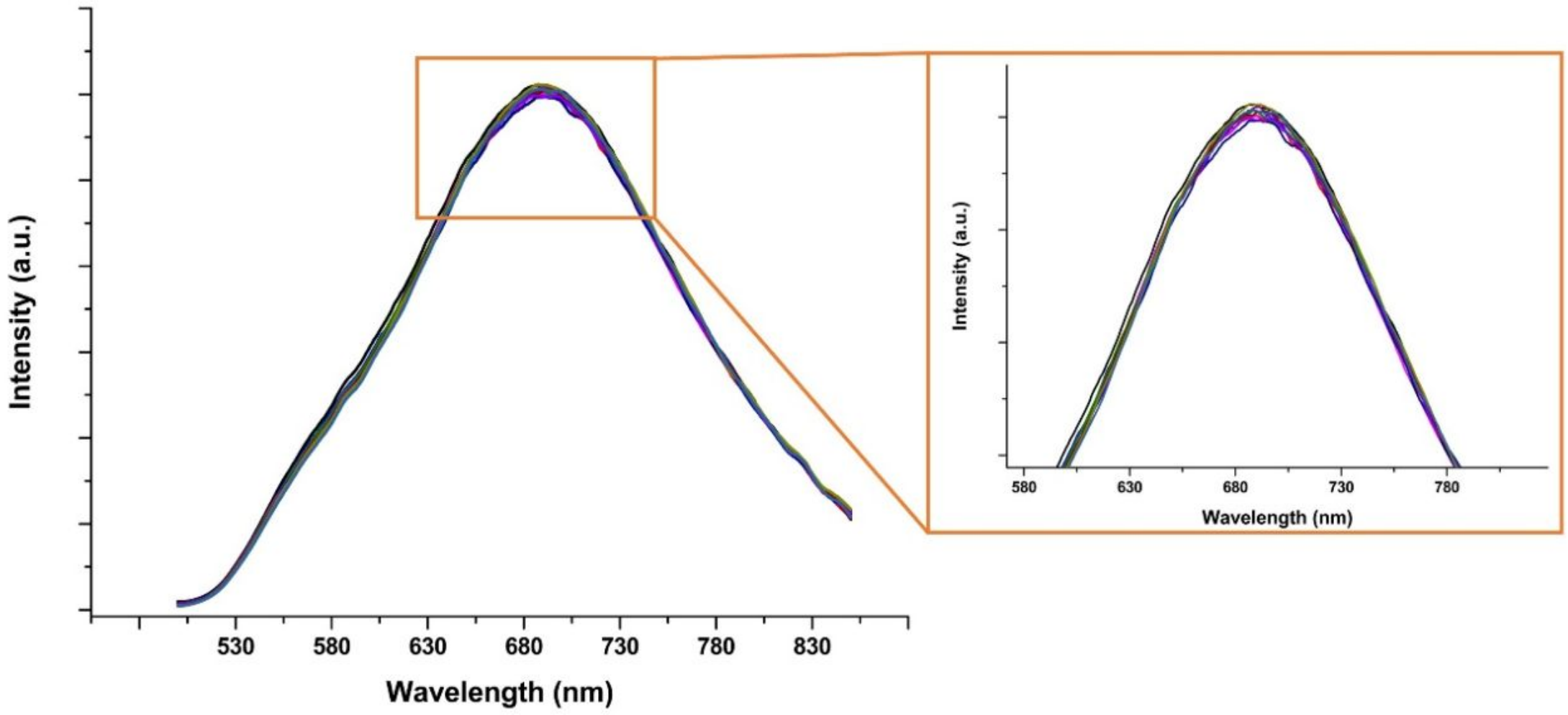

Figure S25. Blank emission measurements of **CP2-US** aqueous suspension in the absence of tetracycline. Experimental conditions: deionized water, room temperature, λ_{ex}= 365 nm, **CP2-US** concentration = 1 mg. mL^{-1}. The suspension was freshly vortexed before each measurement.

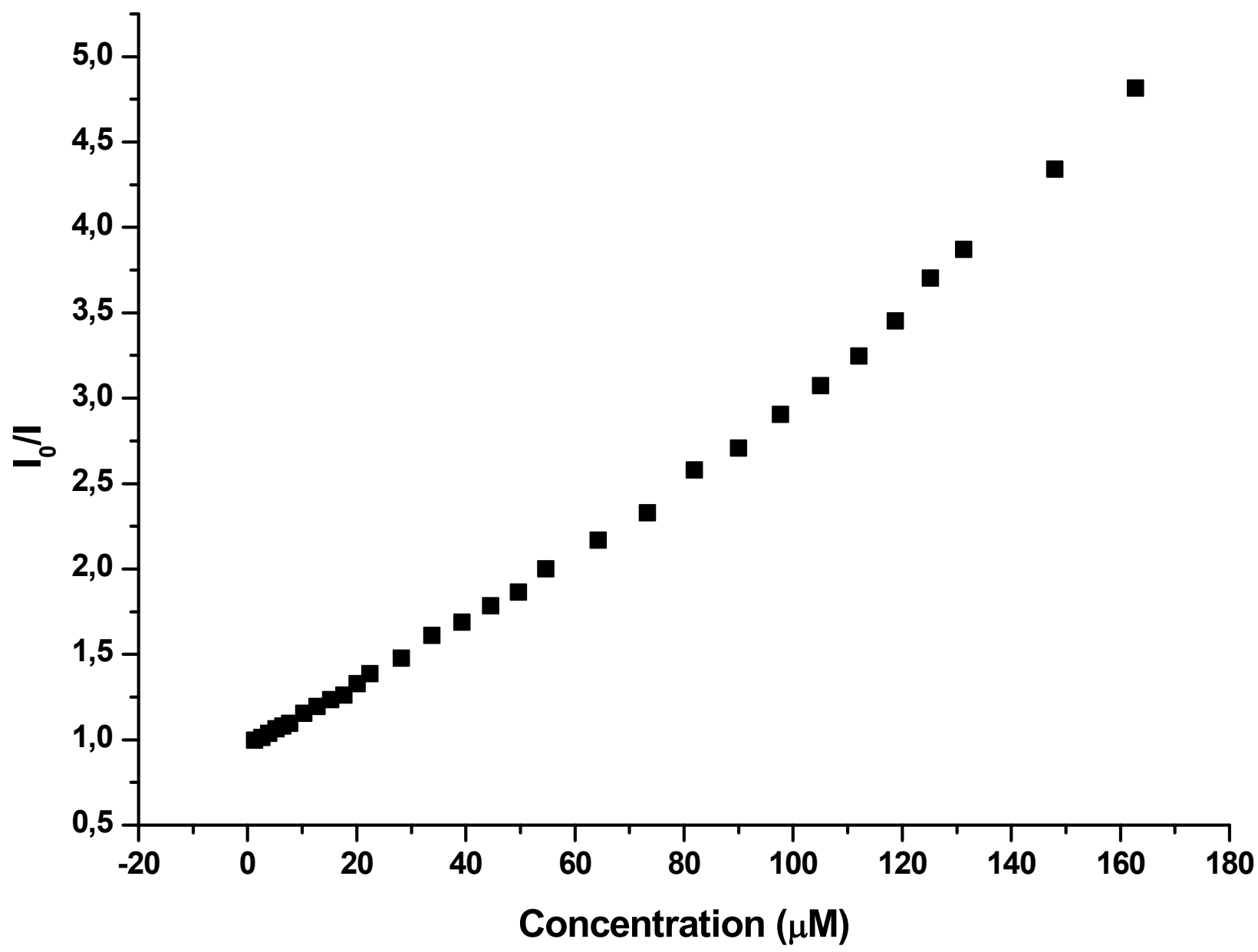

Figure S26. Stern–Volmer plot for the quenching of the emission of **CP1-US** aqueous suspension upon addition of TC. Experimental conditions: deionized water, room temperature,

λ_{ex} = 365 nm, **CP1-US** concentration= 1 mg. mL^{-1}, TC concentration range = 0–180 μM. The suspension was freshly vortexed before each measurement.

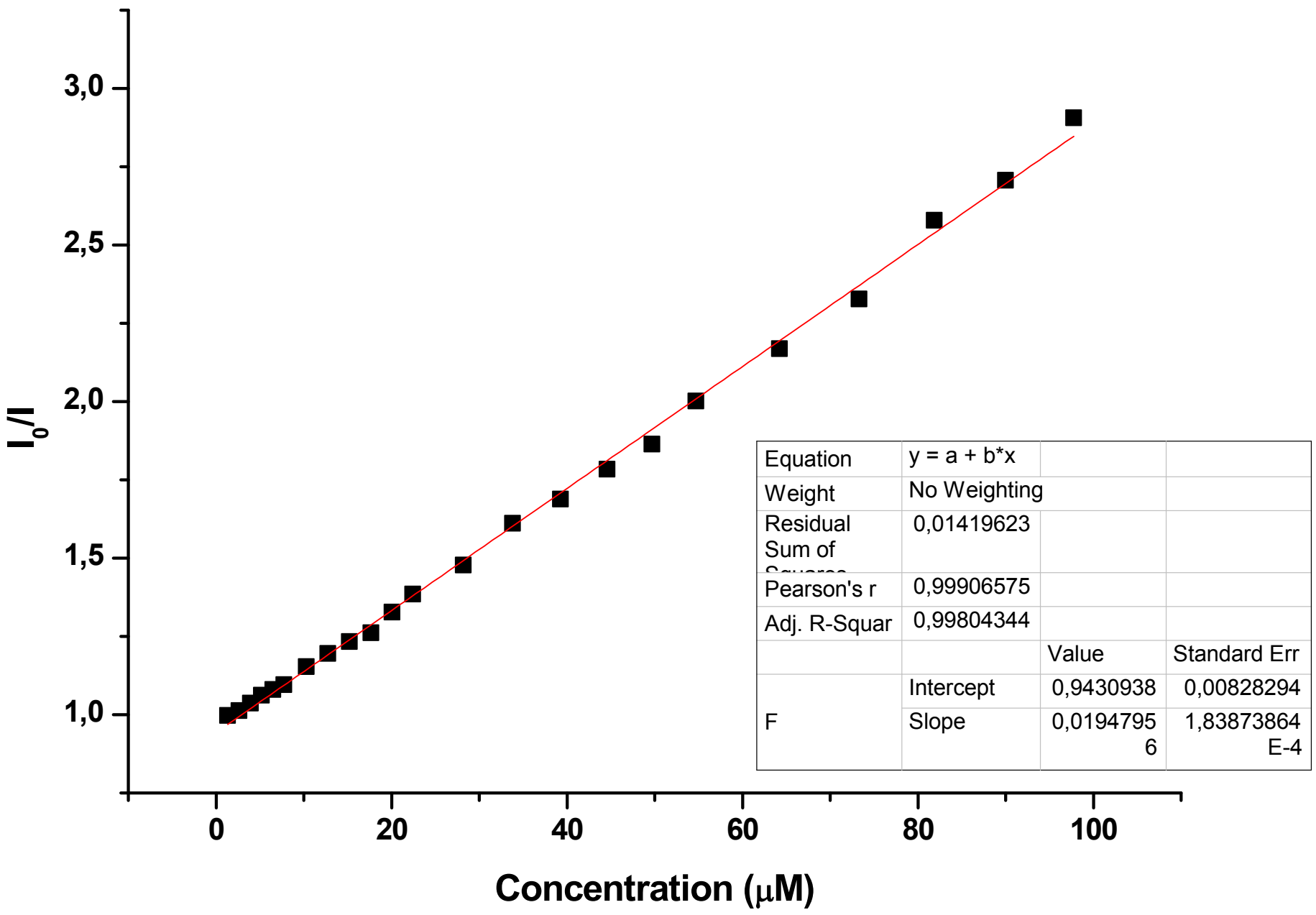

Figure S27. Expanded Stern-Volmer plot in the linear range 0 - 100 μM.

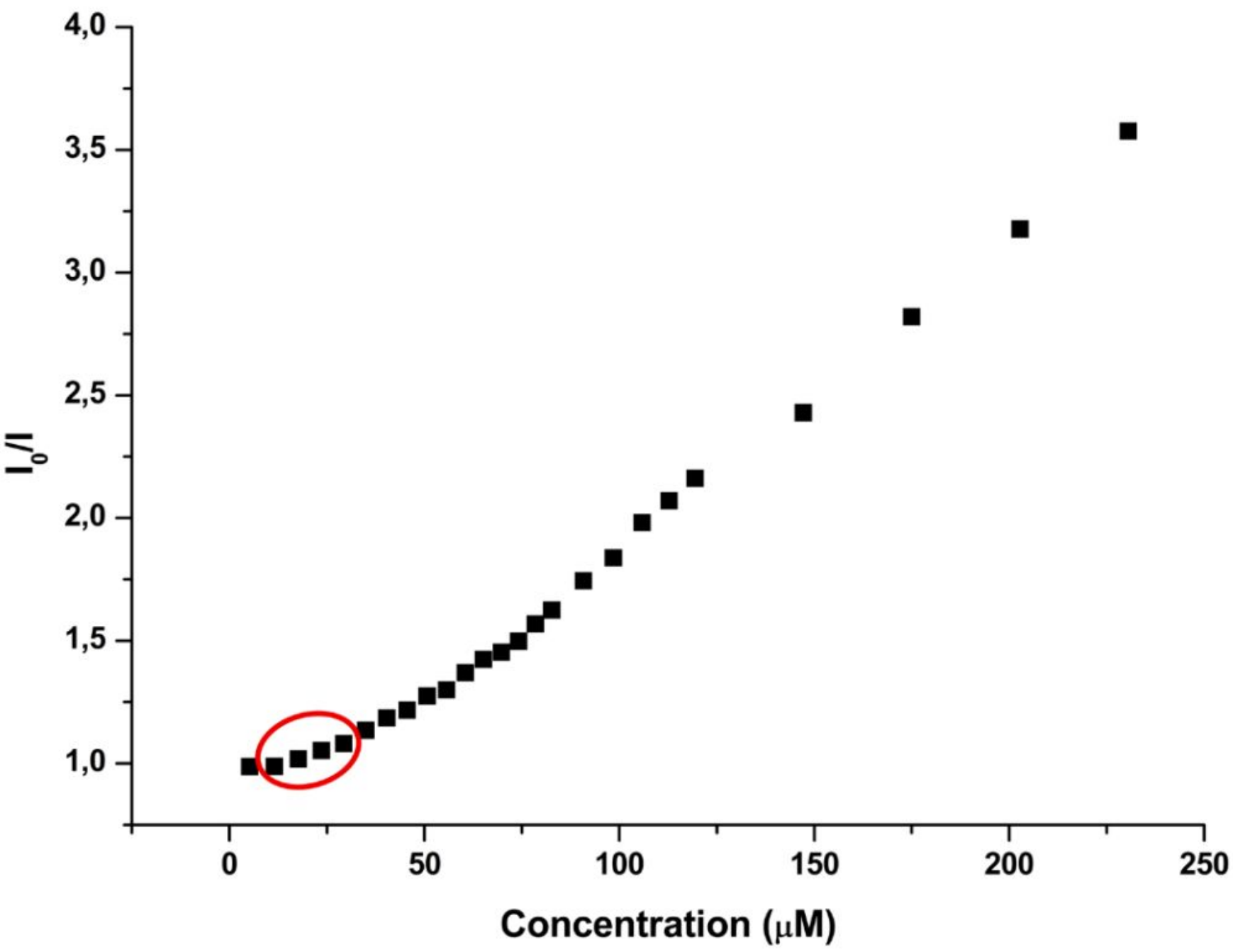

Figure S28. Stern–Volmer plot for the quenching of the emission of **CP2-US** aqueous suspension upon addition of TC. Experimental conditions: deionized water, room temperature, λ_{ex} = 365 nm, **CP2-US** concentration= 1 mg. mL^{-1}, TC concentration range = 0-250 μM. The suspension was freshly vortexed before each measurement.

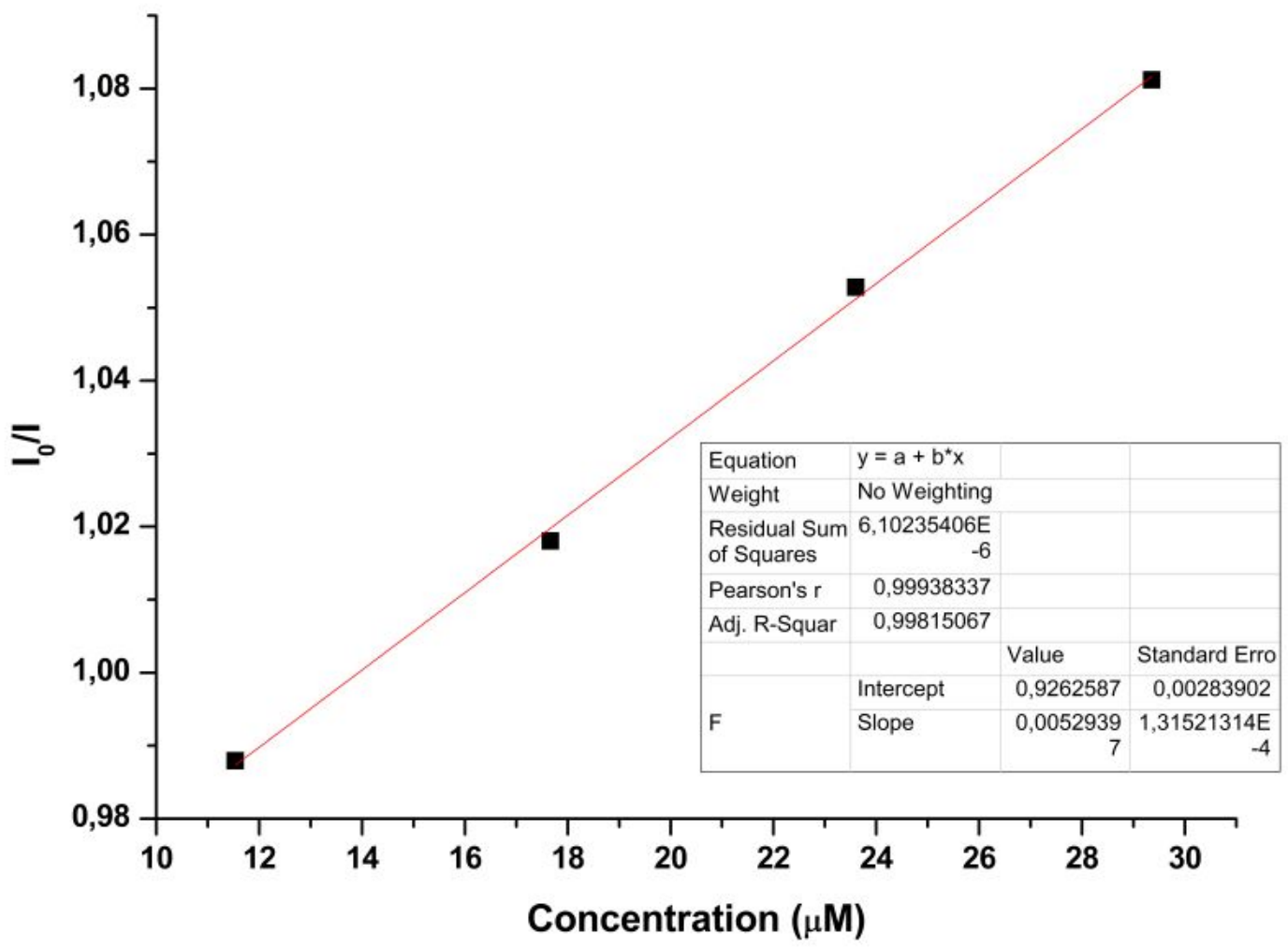

Figure S29. Expanded Stern-Volmer plot in the linear range at low concentrations (12 - 30 μM).

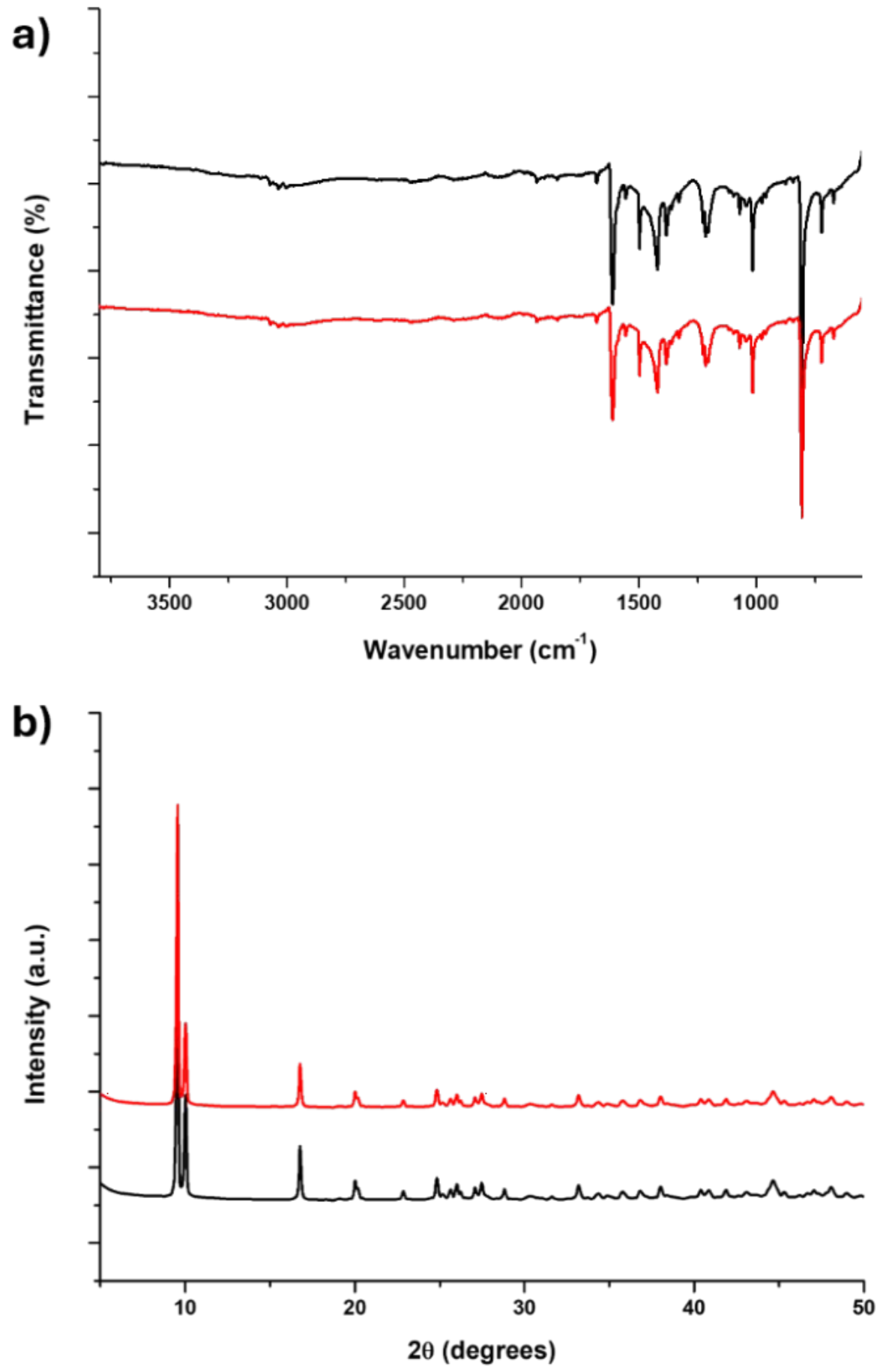

Figure S30. Characterization a) FTIR and b) PXRD of **CP1** after 10 cycles of sensing TC: **CP1-RT** before sensing TC (black line) and **CP1-RT** after sensing TC (red line).

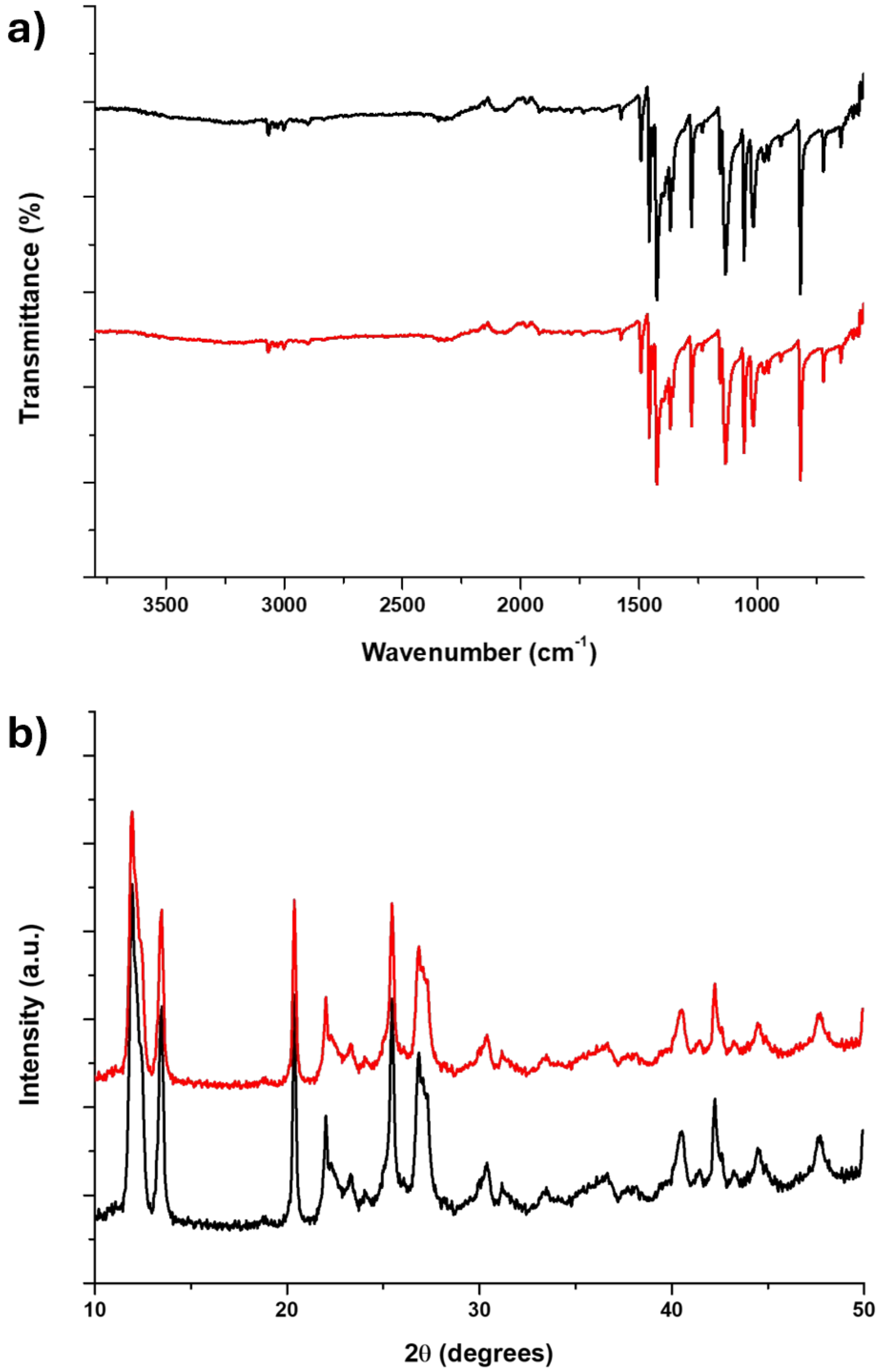

Figure S31. Characterization a) FTIR and b) PXRD of **CP2** after 10 cycles of sensing TC: **CP2-US** before sensing TC (black line) and **CP2-US** after sensing TC (red line).

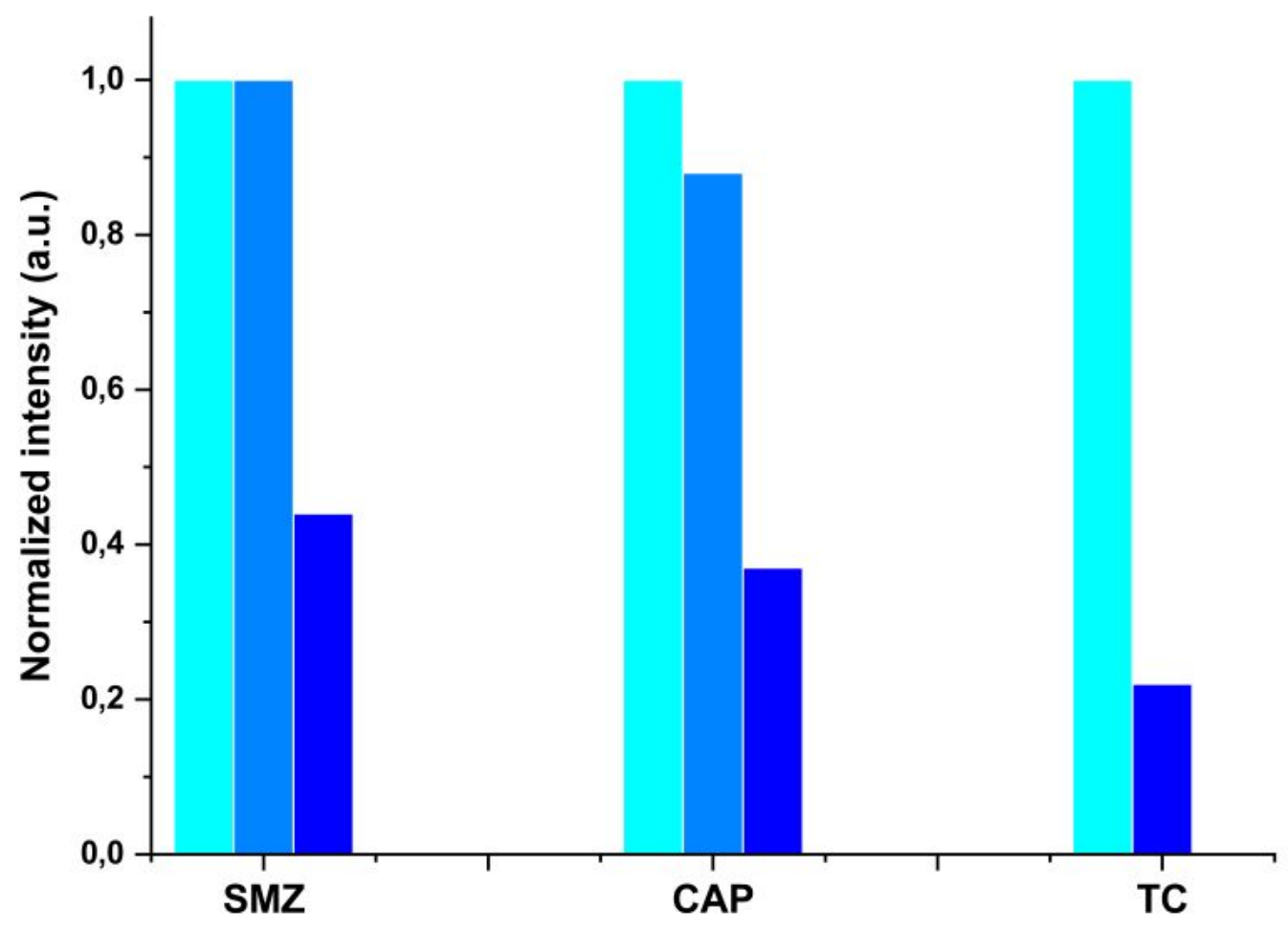

Figure S32. Normalized emission intensity (λ_{ex} = 365 nm) of the aqueous suspension of **CP1-US** particles (5mg, 1 mg. mL^{-1}) after reacting for 1 minute with 0.80 μmol of various commonly used antibiotics. Light blue bar (blank), blue bar (interfering agent: SMZ or CAP), dark blue bar (TC). Measurements were taken at room temperature.

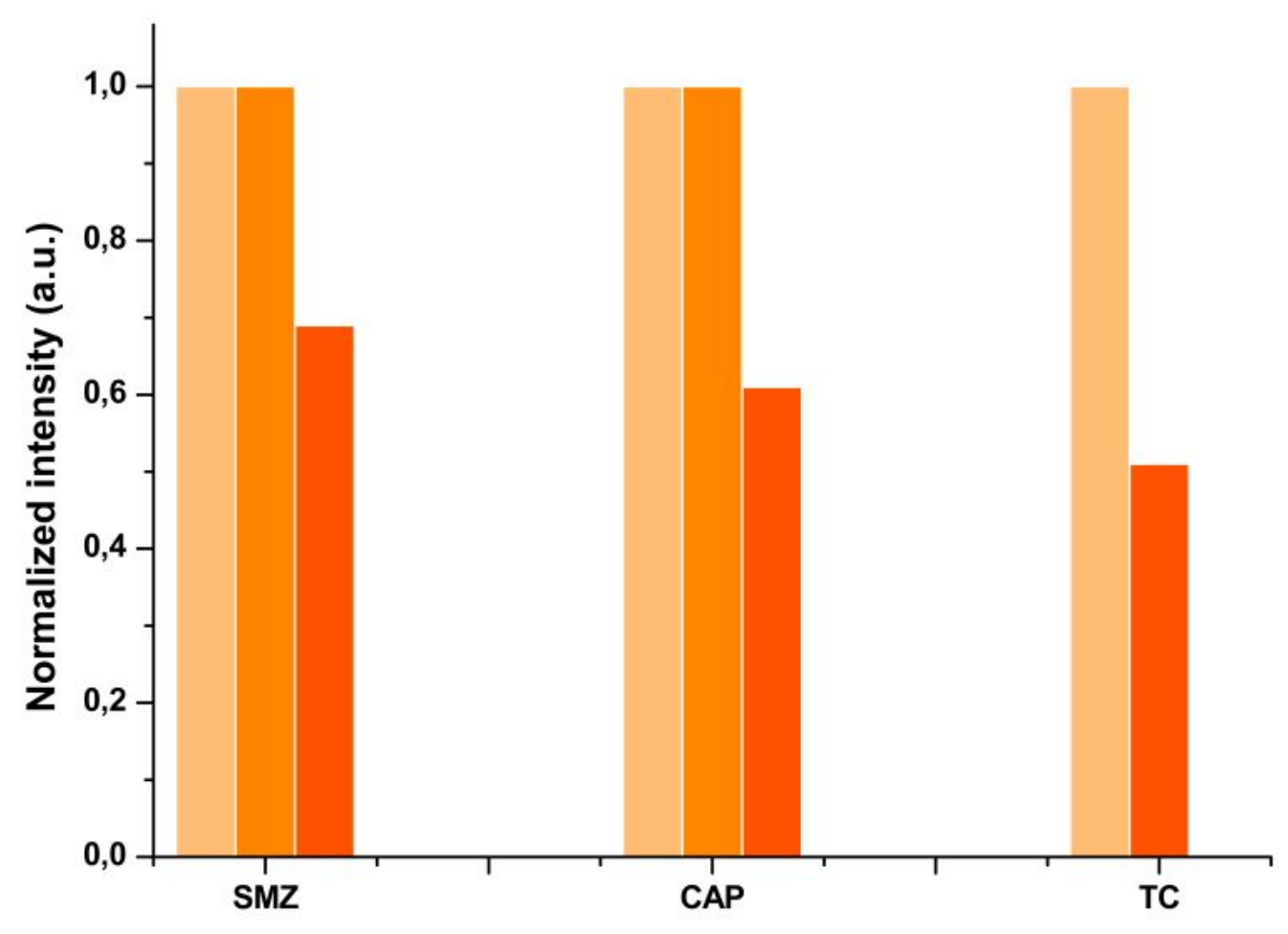

Figure S33. Normalized emission intensity (λ_{ex} = 365 nm) of the aqueous suspension of **CP2-US** particles (5mg, 1 mg. mL^{-1}) after reacting for 1 minute with 0.80 μmol of various

commonly used antibiotics. Light orange bar (blank), orange bar (interfering agent: SMZ or CAP), dark orange bar (TC). Measurements were taken at room temperature.

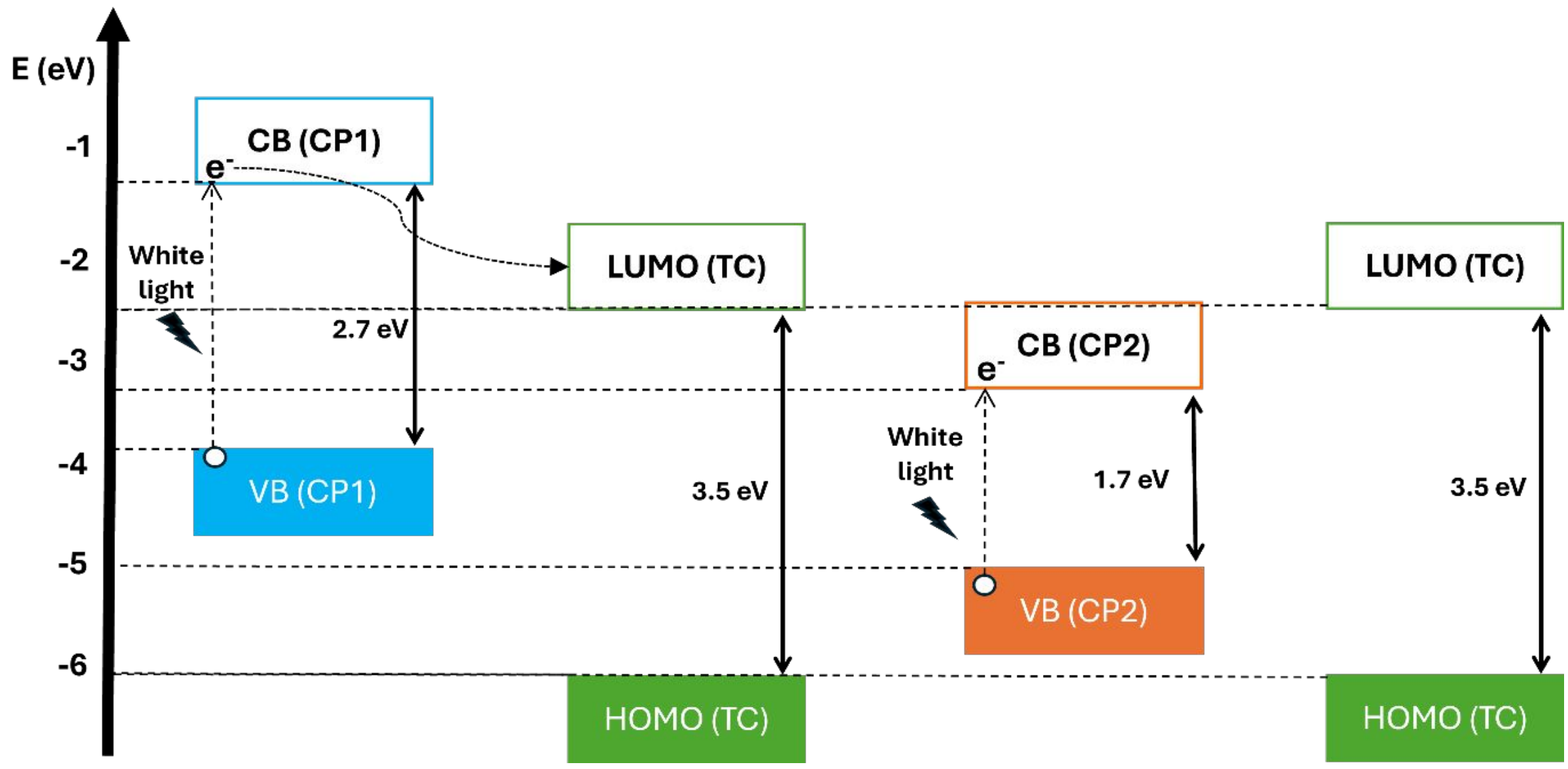

Figure S34. Energy values of the conduction band (CB) and valence band (VB) edges of **CP1** and **CP2**, and the HOMO-LUMO values of tetracycline (TC).

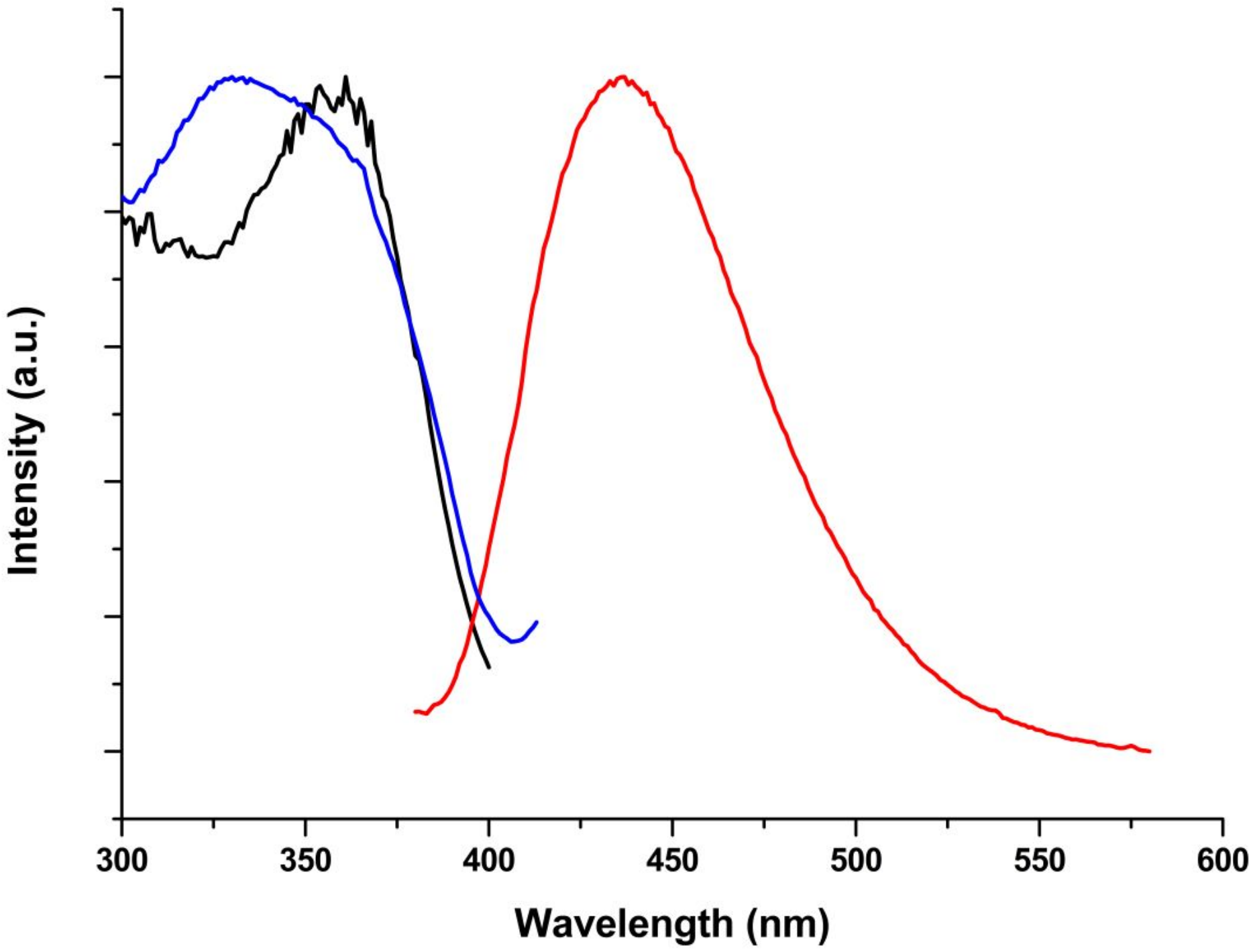

Figure S35. Spectral overlap between the absorption (blue) and emission (red) spectra of **CP1-US** and the absorption spectrum of TC (black).

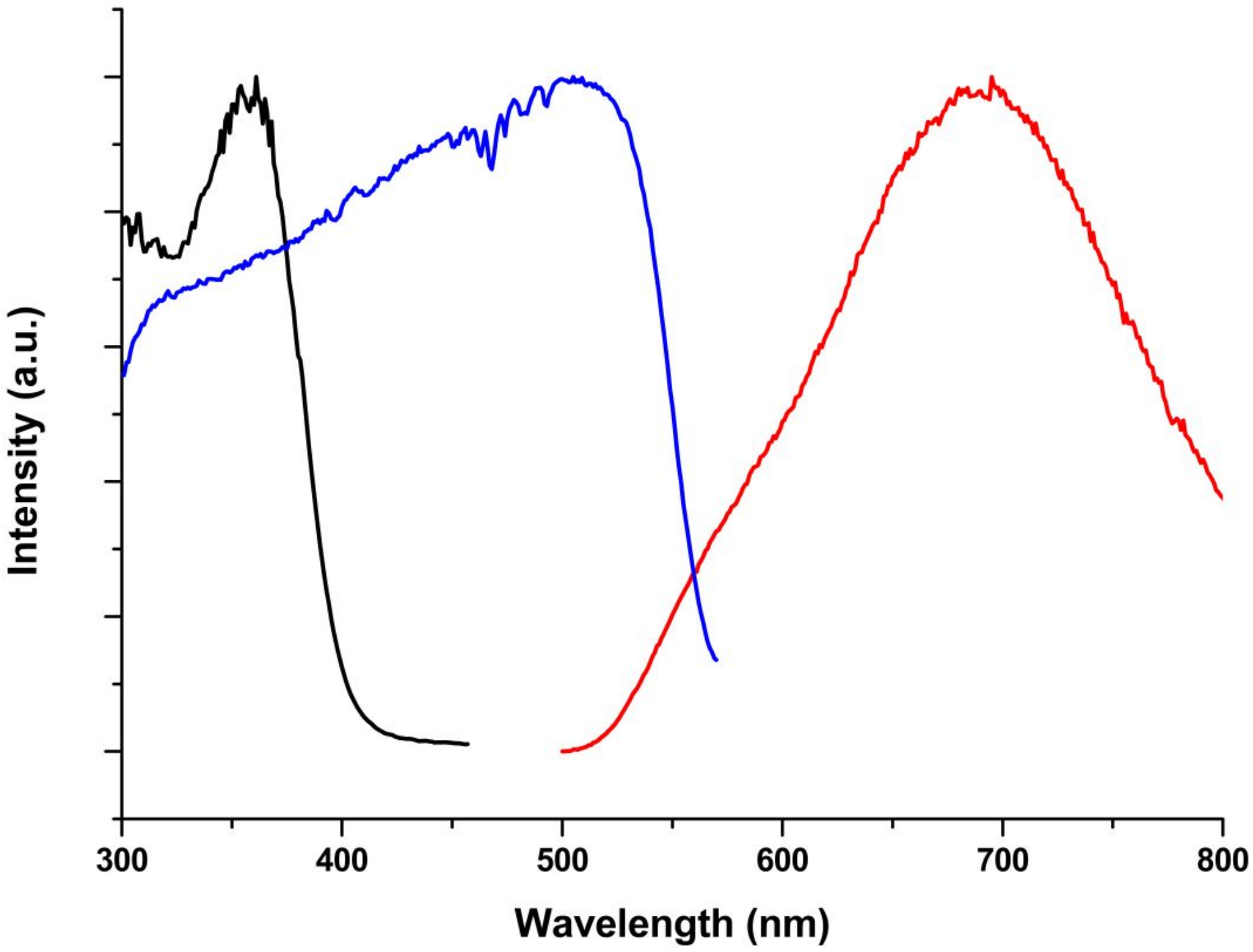

Figure S36. Spectral overlap between the absorption (blue) and emission (red) spectra of **CP2-US** and the absorption spectrum of TC (black).

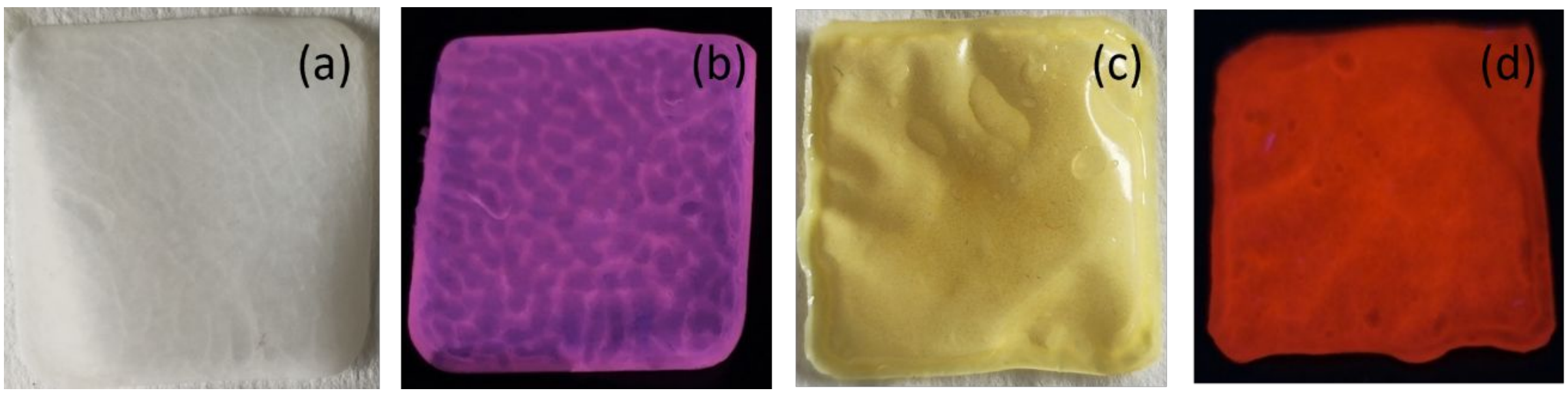

Figure S37. **CP1-US** and **CP2-US** portable devices: drop casting in dioxane viewed under visible light of **CP1-US** (a) and **CP2-US** (c); and drop casting in dioxane viewed under UV light at 365 nm of **CP1-US** (b) and **CP2-US** (d).

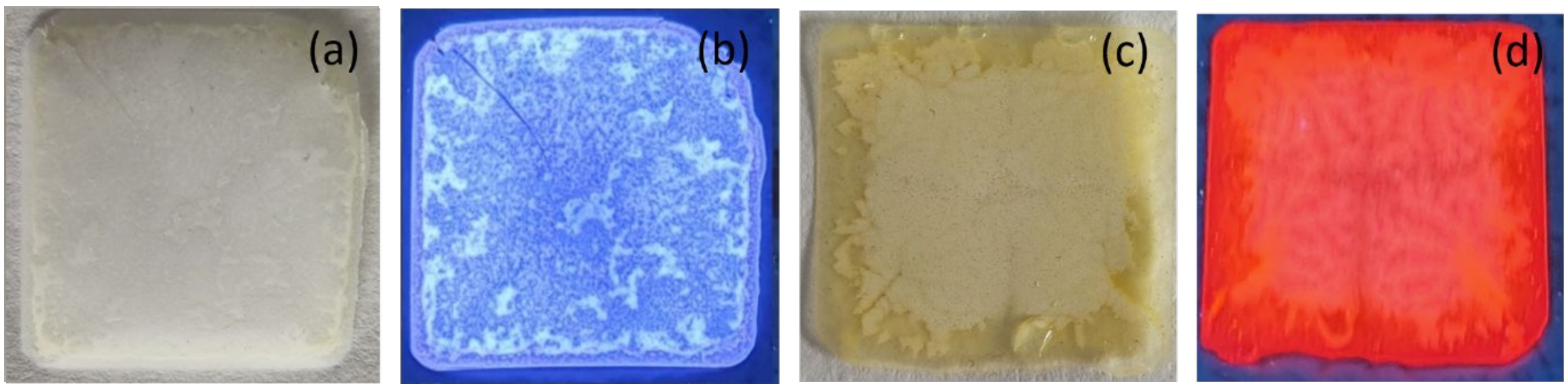

Figure S38. **CP1-US** and **CP2-US** portable devices: drop casting in acetonitrile at 60 °C viewed under visible light of **CP1-US** (a) and **CP2-US** (c); and drop casting in acetonitrile at 60 °C viewed under UV light at 365 nm of **CP1-US** (b) and **CP2-US** (d).

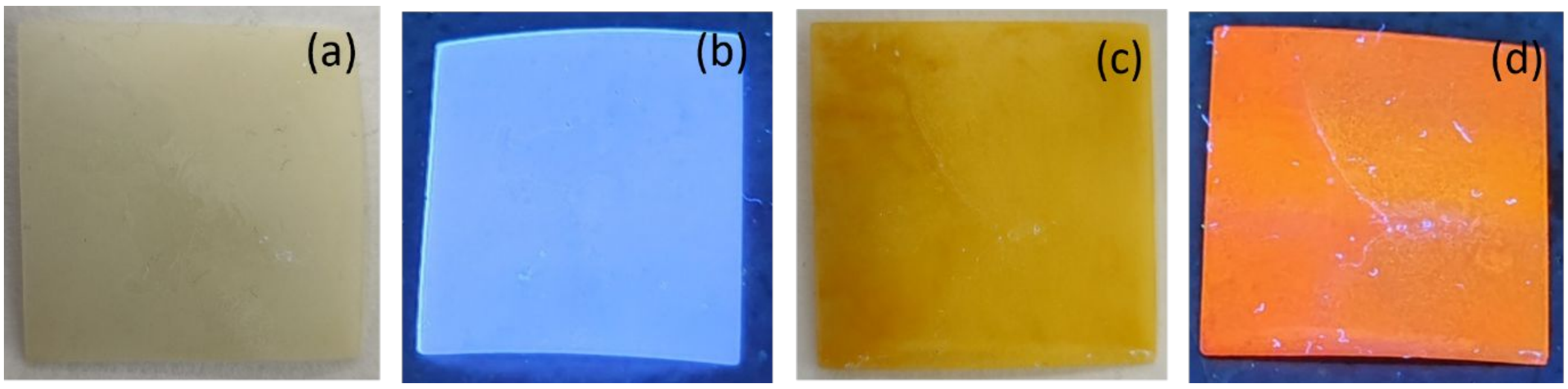

Figure S39. **CP1-US** and **CP2-US** portable devices: 3D printing under visible light of **CP1-US** (a) and **CP2-US** (c); and 3D printing under UV light at 365 nm of **CP1-US** (b) and **CP2-US** (d).

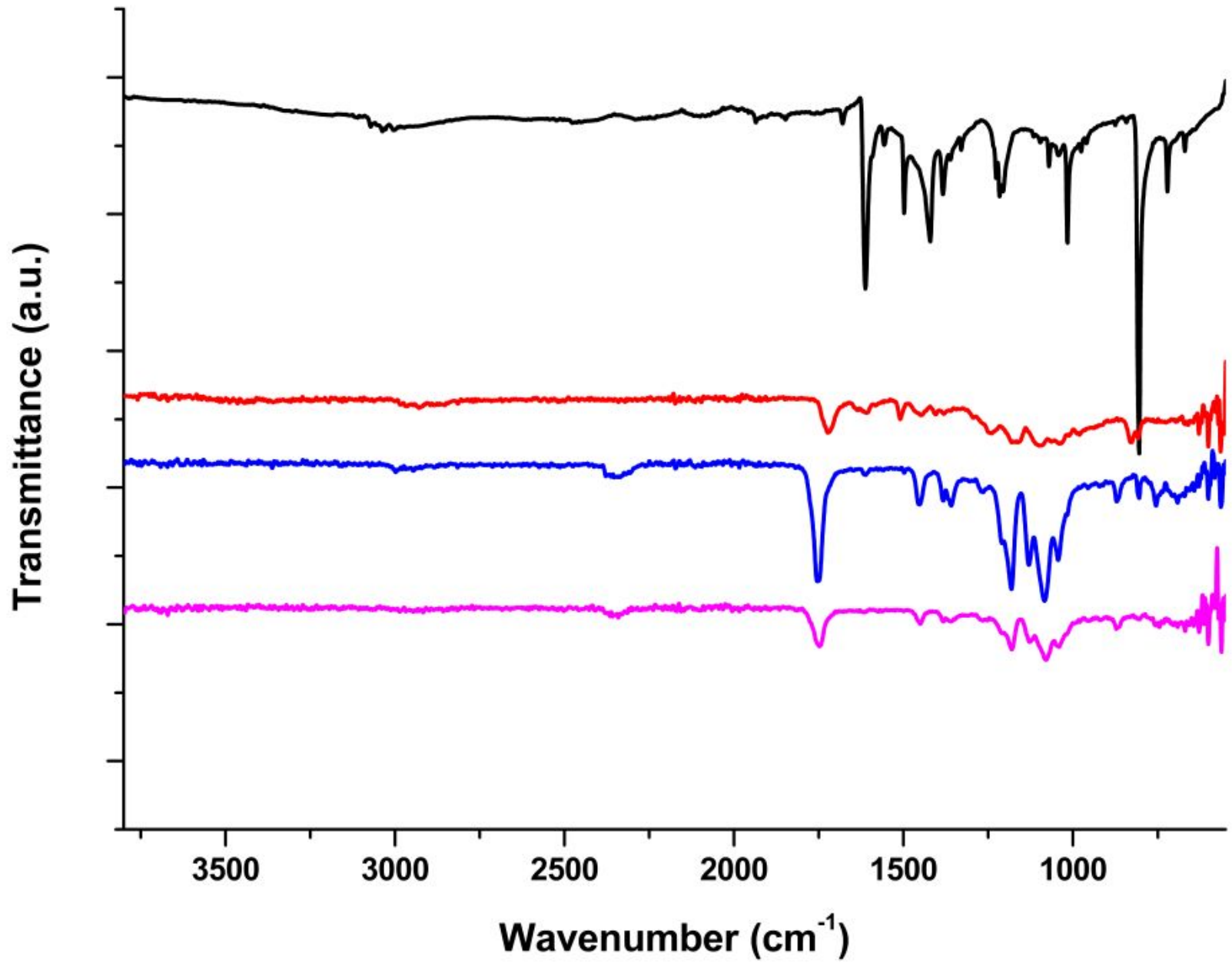

Figure S40. IR spectrum of experimental of **CP1-US** particles (black), **CP1-3D** (red), **CP1-DC** in acetonitrile (blue) and **CP1-DC** in dioxane (pink).

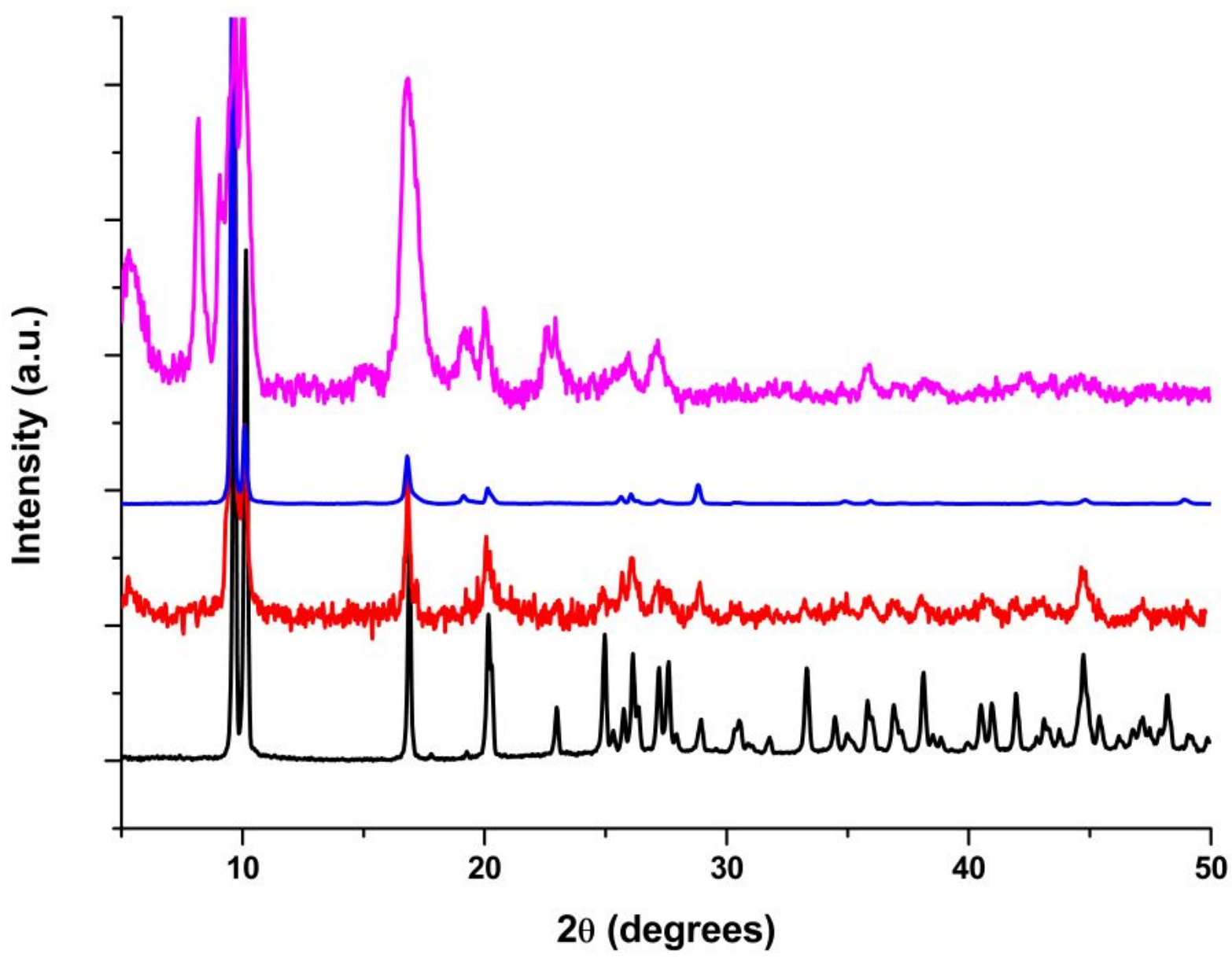

Figure S41. X-ray powder diffractograms (PXRD) of **CP1-US** particles (black), **CP1-3D** (red), **CP1-DC** in acetonitrile (blue) and **CP1-DC** in dioxane (pink).

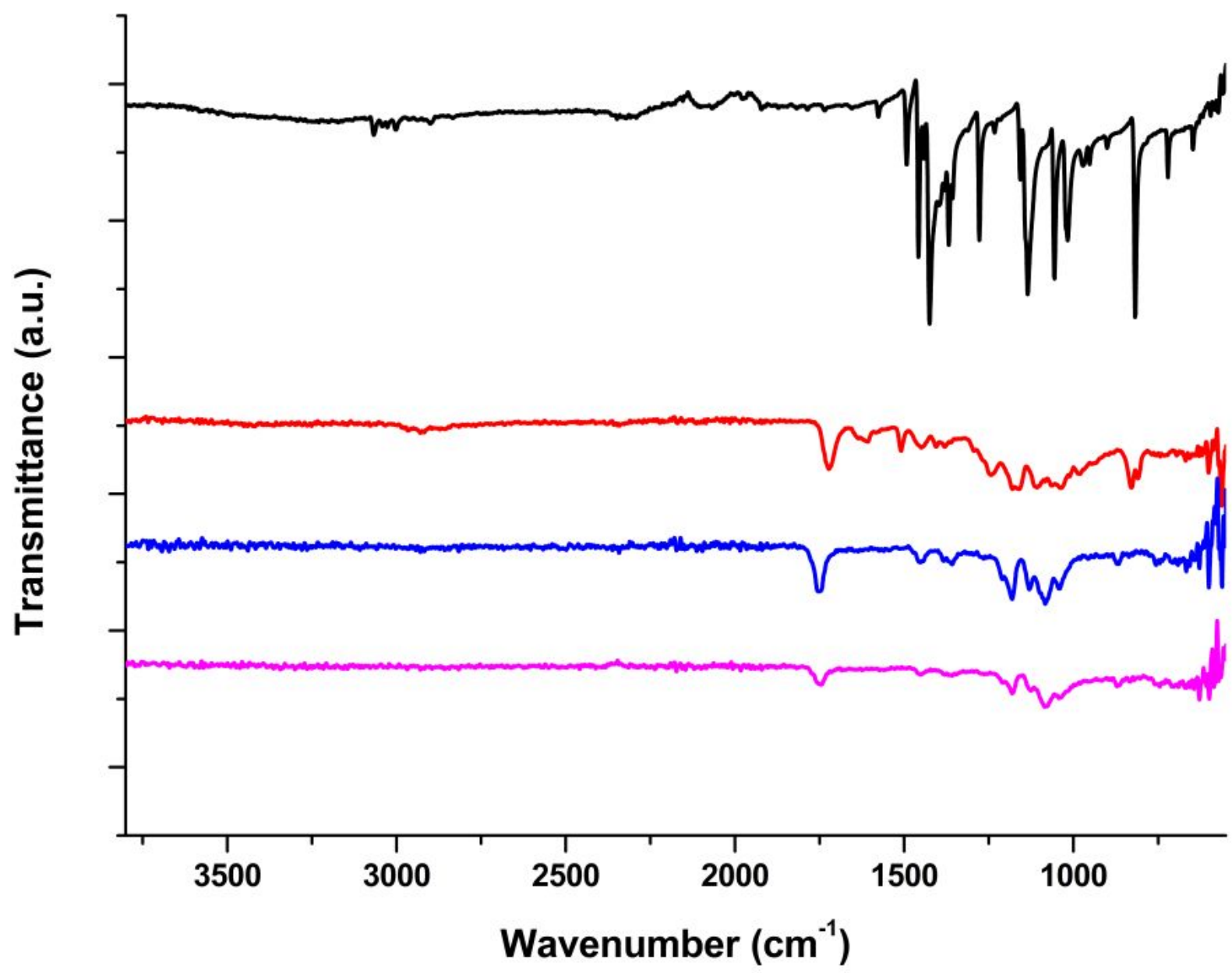

Figure S42. IR spectrum of experimental of **CP2-US** particles (black), **CP2-3D** (red), **CP2-DC** in acetonitrile (blue) and **CP2-DC** in dioxane (pink).

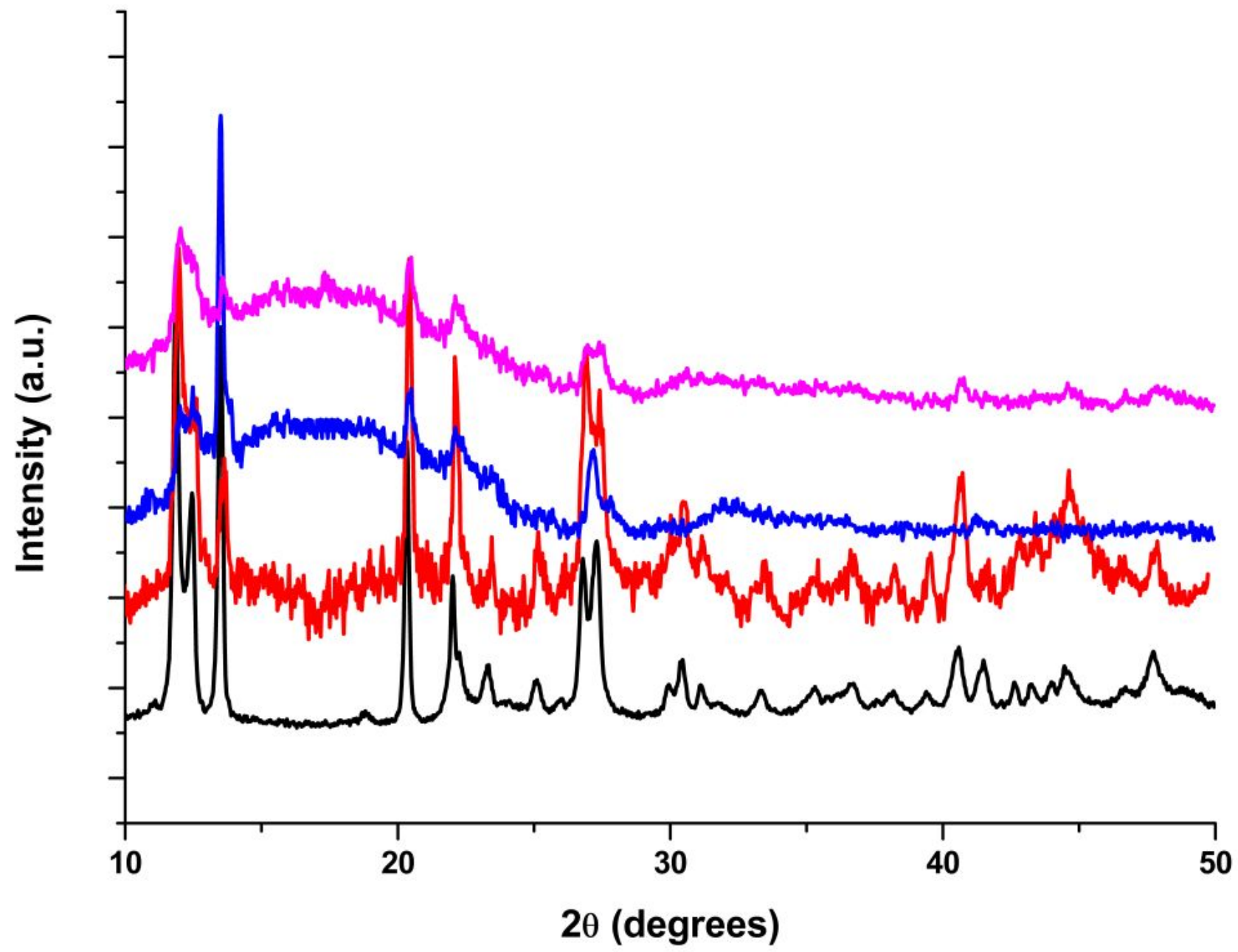

Figure S43. X-ray powder diffractograms (PXRD) of **CP2-US** particles (black), **CP2-3D** (red), **CP2-DC** in acetonitrile (blue) and **CP2-DC** in dioxane (pink).

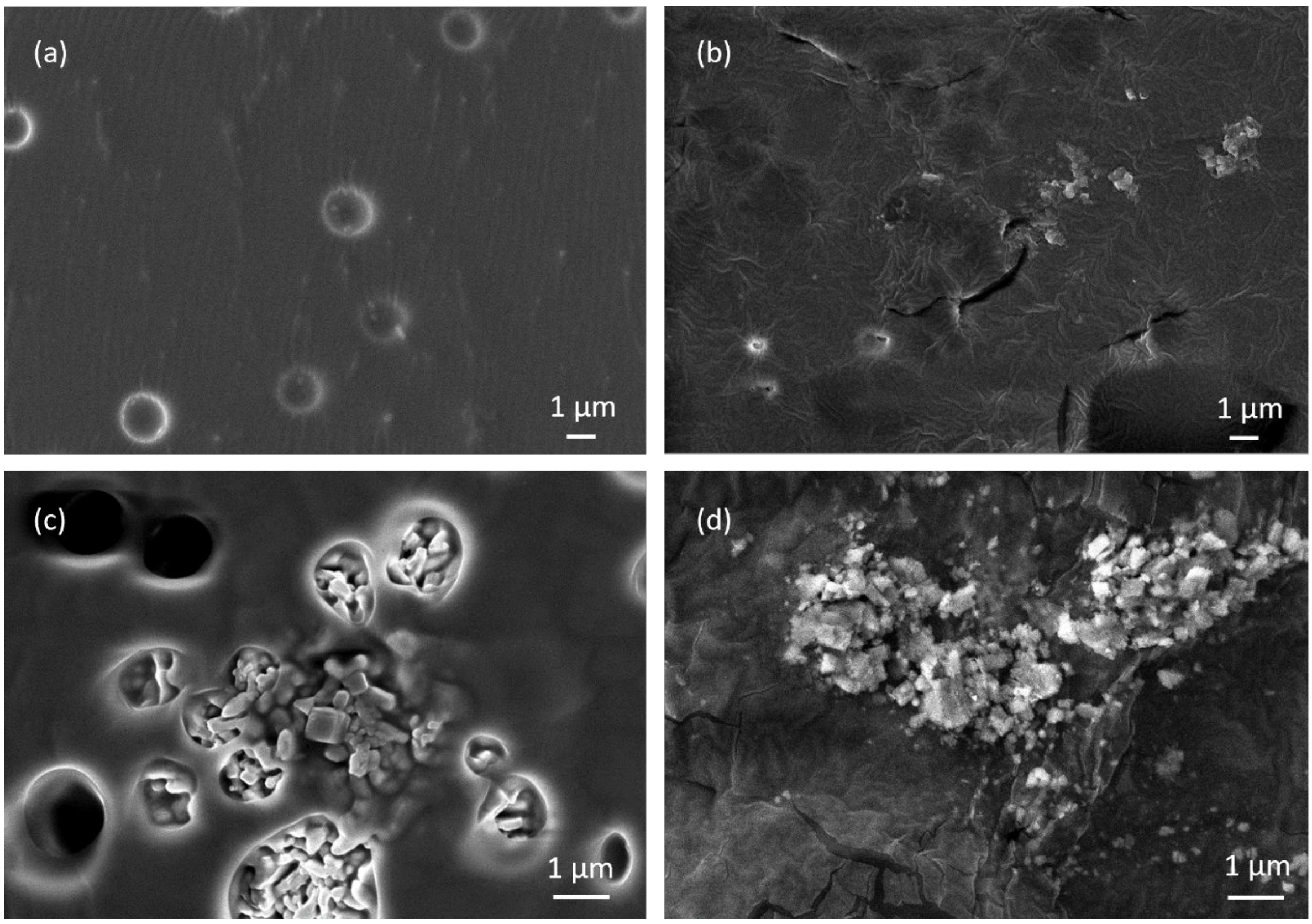

Figure S44. SEM micrographs of **CP1-3D** (a), **CP1-DC** (b), **CP2-3D** in acetonitrile (c) and **CP2-DC** in acetonitrile (d).

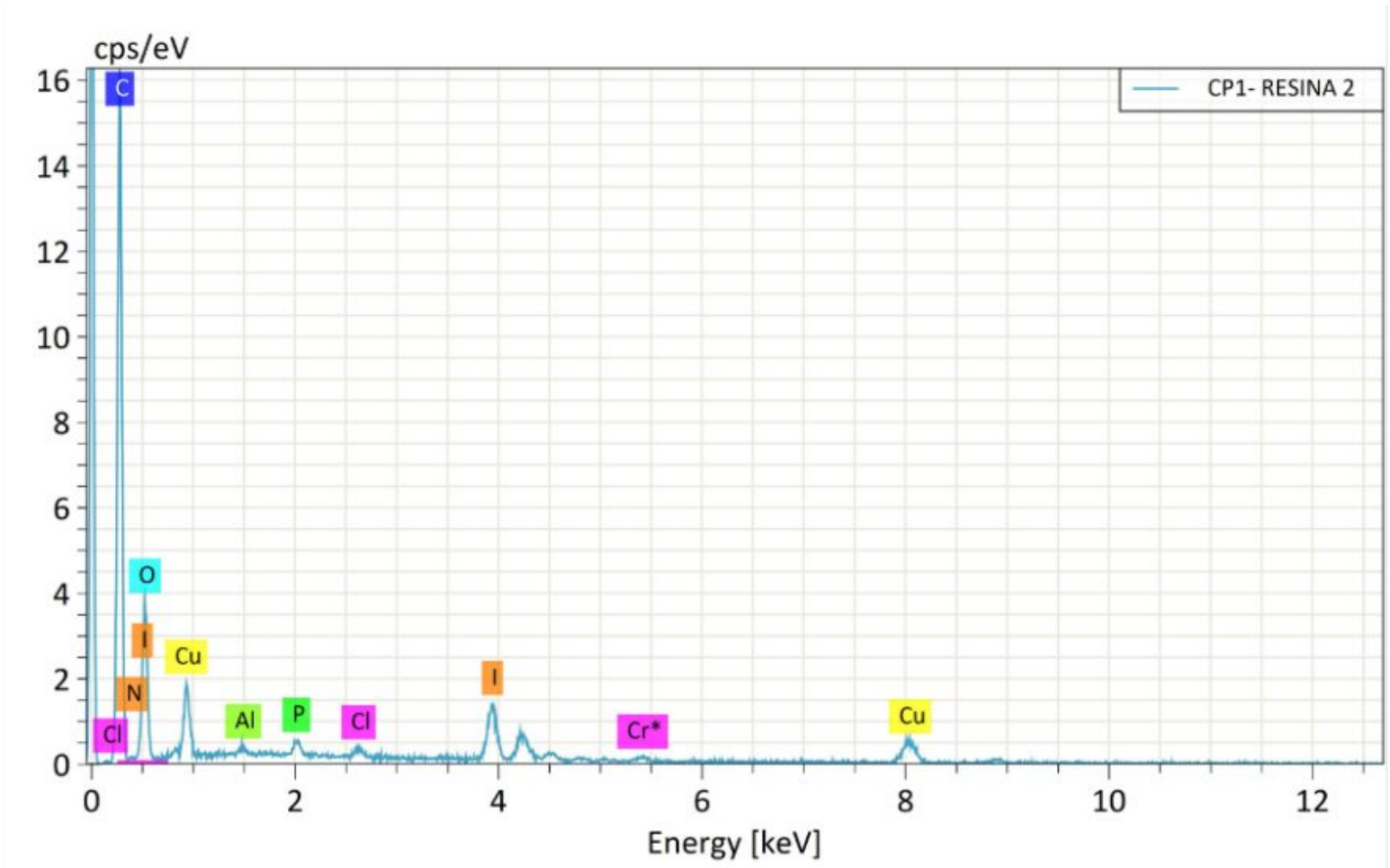

Element	Line series	Mass Norm. [%]	Atom [%]	abs. error [mass%] (1 σ)
C	K	57.02	71.65	3.22
O	K	22.95	21.65	1.42
N	K	3.40	3.66	0.33
Cu	K	5.94	1.41	0.40
I	L	9.69	1.15	0.50
P	K	0.47	0.23	0.04
Cl	K	0.36	0.15	0.03
Al	K	0.17	0.10	0.03
Cr	K	0.00	0.00	0.00
		100.00	100.00	

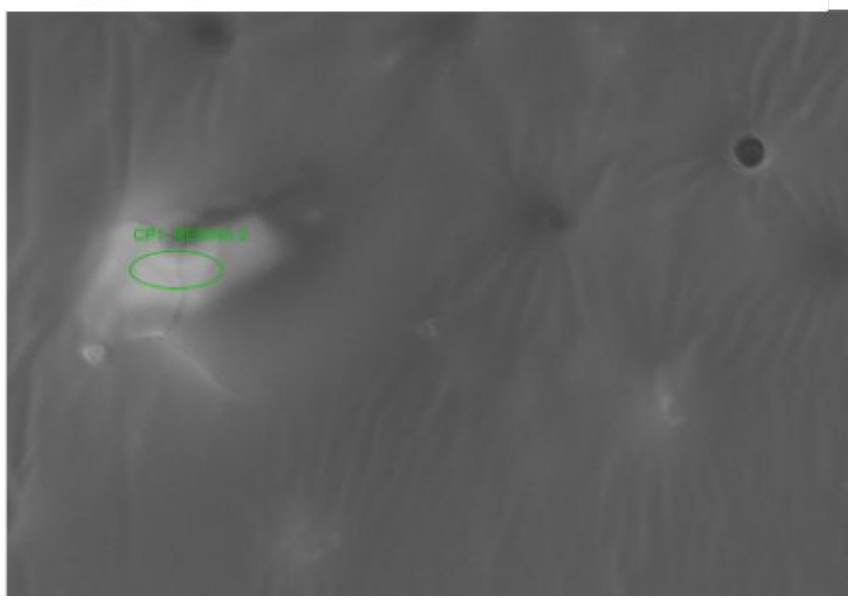

Figure S45. SEM-EDX of **CP1-3D** in acetonitrile.

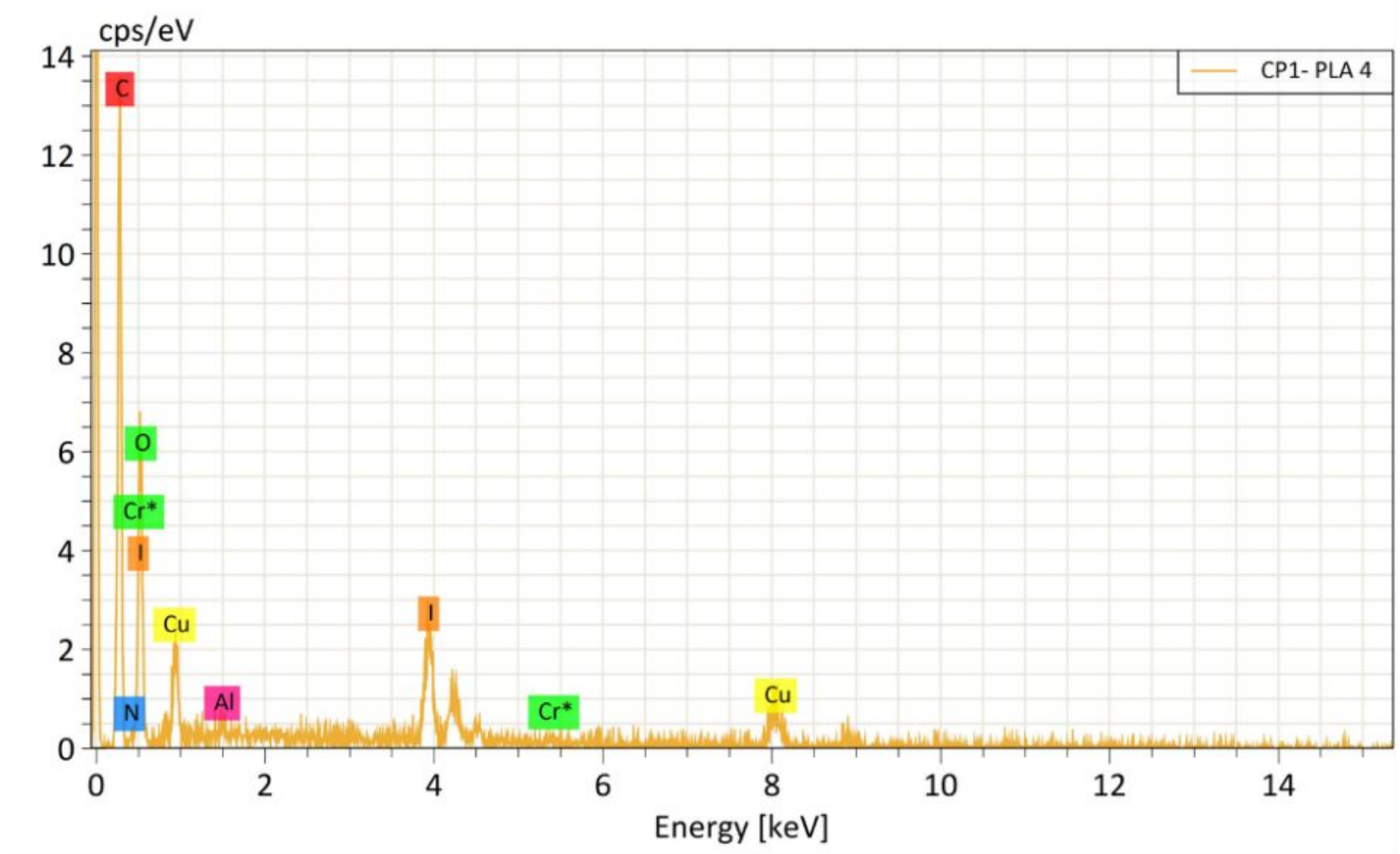

CP1- PLA 4

Element	Line series	Mass Norm. [%]	Atom [%]	abs. error [mass%] (1 σ)
C	K	45.35	63.90	2.66
O	K	27.72	29.32	1.80
Cu	K	9.55	2.54	1.35
I	L	15.44	2.06	1.60
N	K	1.65	1.99	0.50
Al	K	0.29	0.18	0.09
Cr	K	0.00	0.00	0.00
		100.00	100.00	

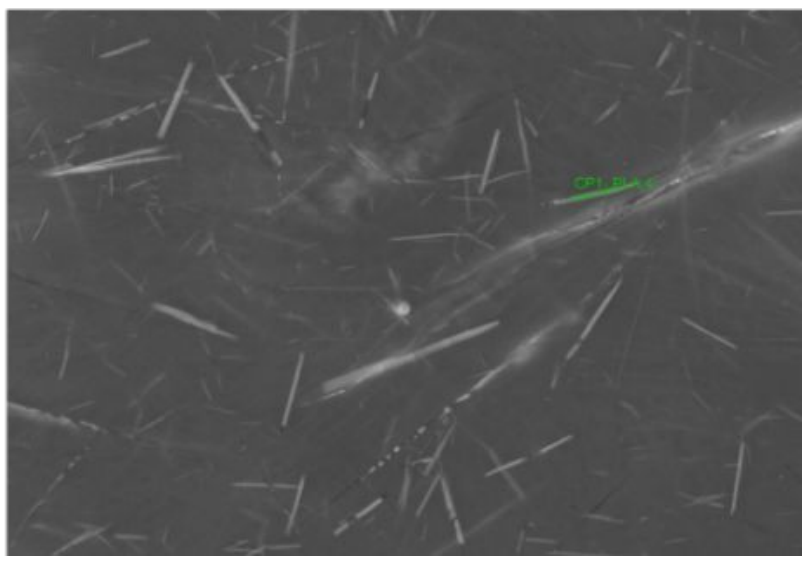

Figure S46. SEM-EDX of **CP1-DC**.

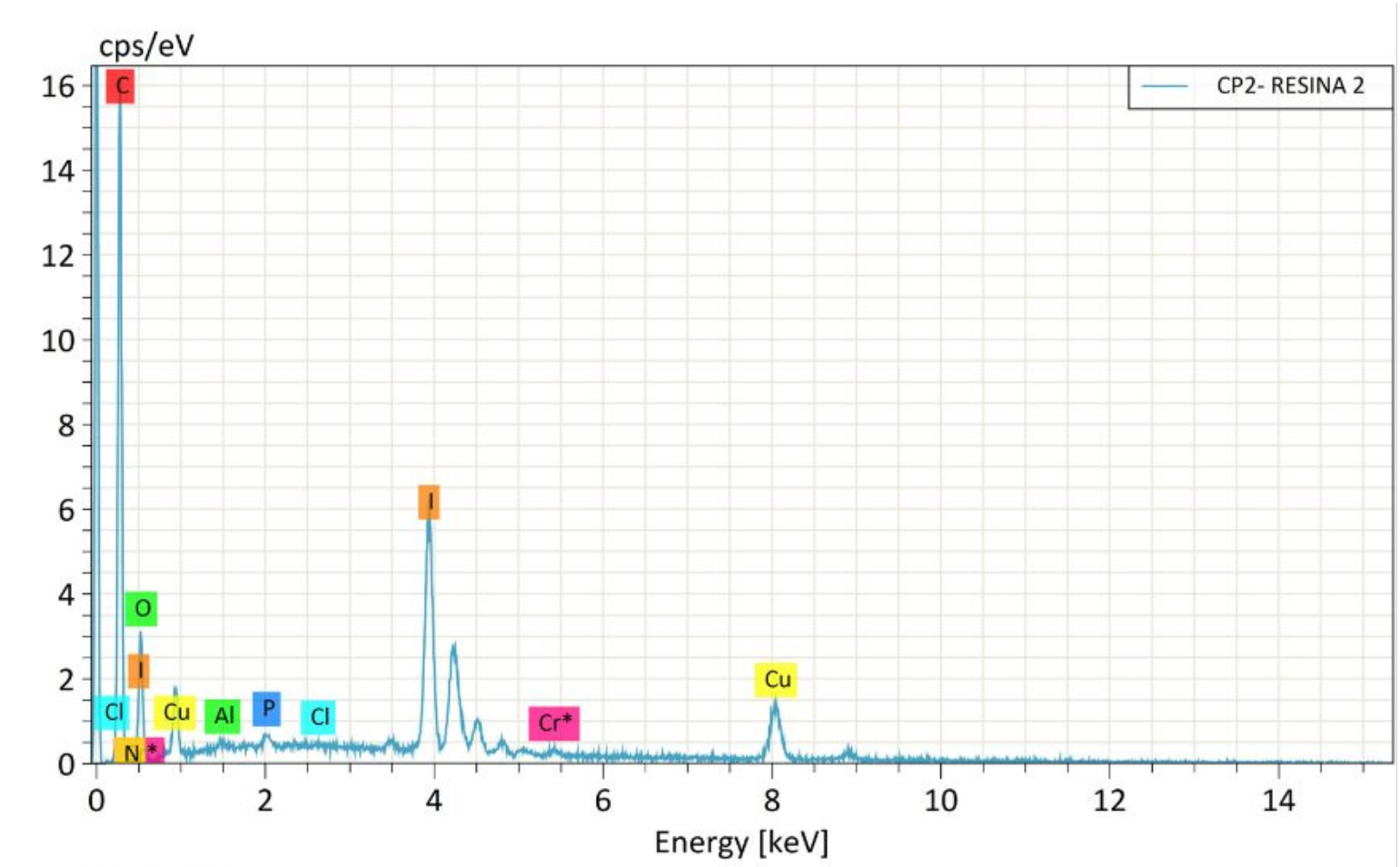

CP2- RESINA 2

Element	Line series	Mass Norm. [%]	Atom [%]	abs. error [mass%] (1 σ)
C	K	45.04	76.78	2.10
O	K	10.24	13.11	0.51
I	L	31.06	5.01	0.84
Cu	K	12.60	4.06	0.44
N	K	0.41	0.60	0.08
P	K	0.35	0.23	0.03
Al	K	0.22	0.17	0.03
Cl	K	0.06	0.04	0.02
Cr	K	0.00	0.00	0.00
		100.00	100.00	

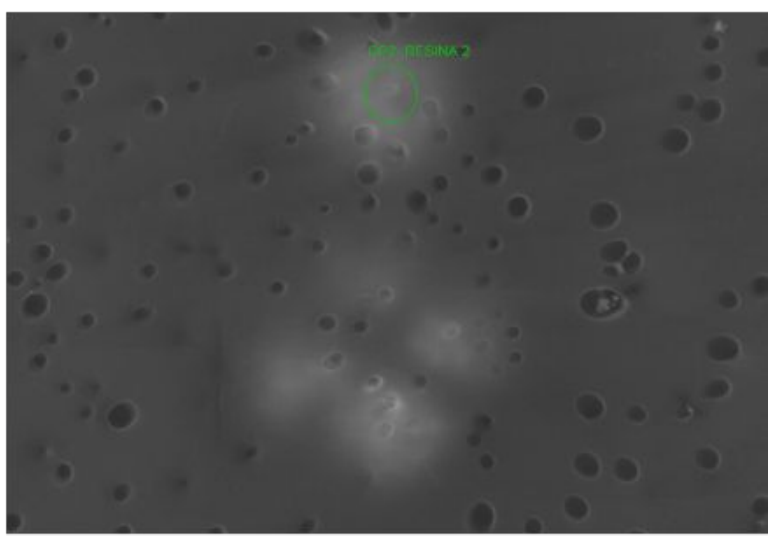

Figure S47. SEM-EDX of **CP2-3D** in acetonitrile.

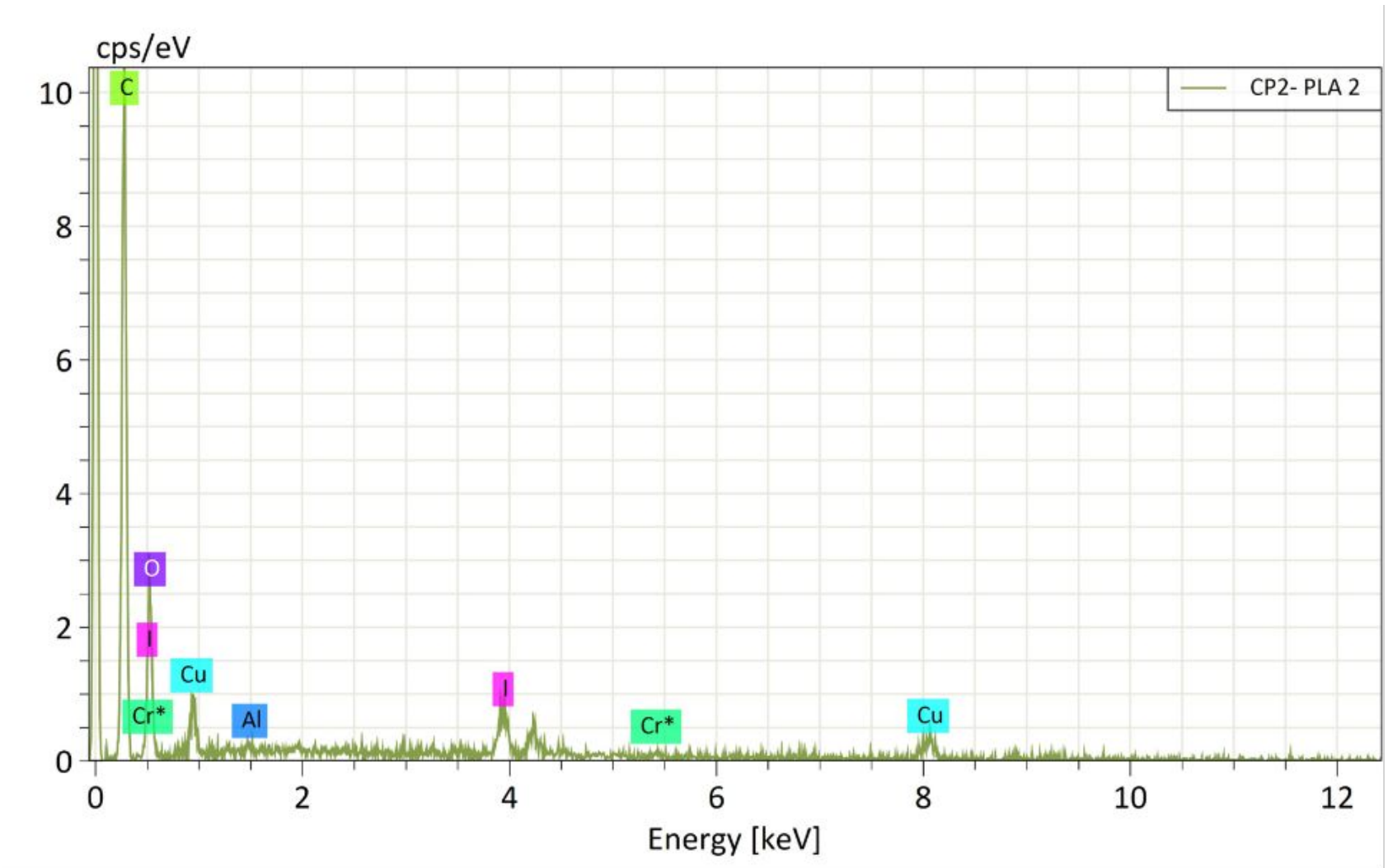

CP2- PLA 2

Element	Line series	Mass Norm. [%]	Atom [%]	abs. error [mass%] (1 σ)
C	K	58.92	73.25	2.48
O	K	25.83	24.10	1.29
Cu	K	6.24	1.47	0.62
I	L	8.74	1.03	0.63
Al	K	0.28	0.15	0.05
Cr	K	0.00	0.00	0.00
		100.00	100.00	

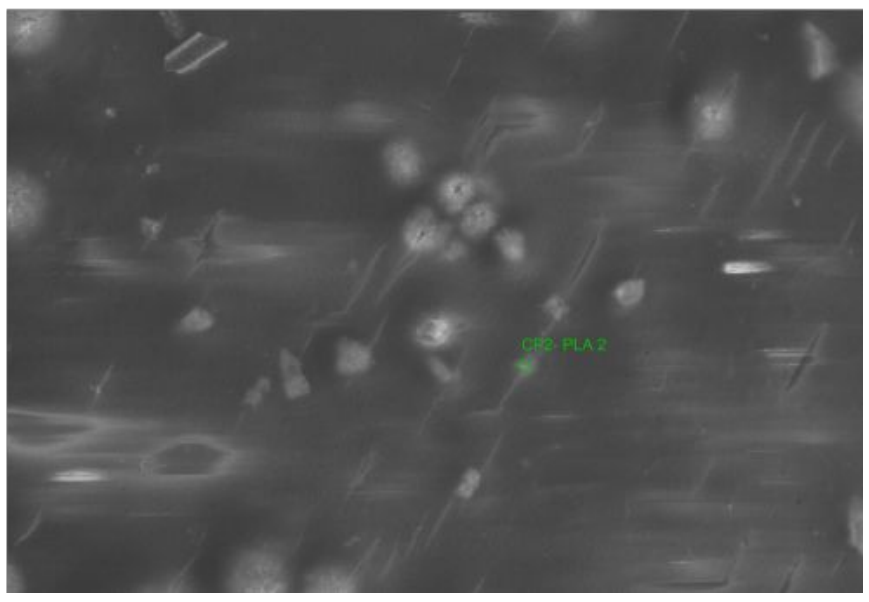

Figure S48. SEM-EDX of **CP2-DC**.

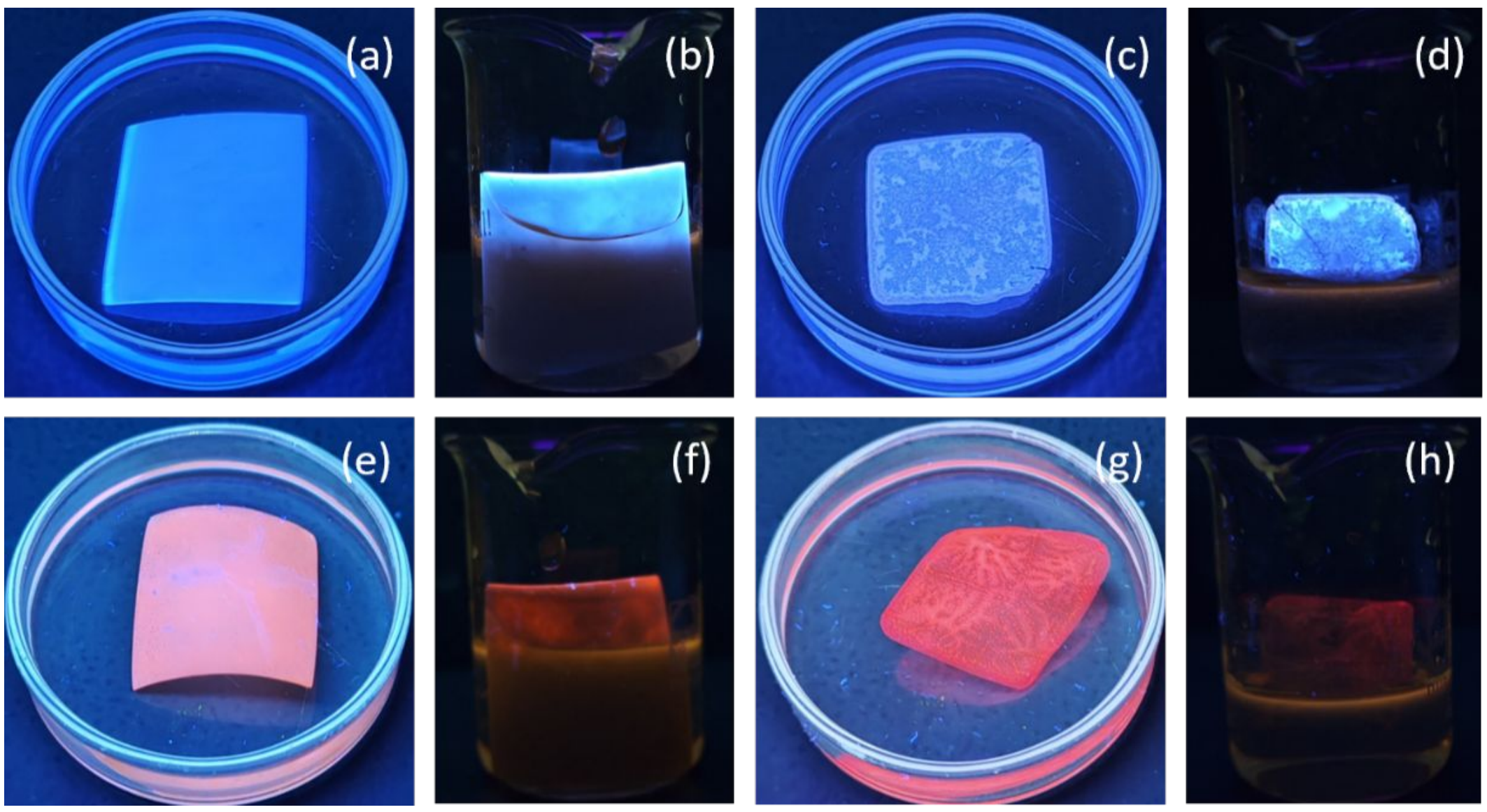

Figure S49. CP1-US (top) and **CP2-US** (bottom) portable devices viewed under UV light at 365 nm: **CP@3D** in water (a,e), **CP@3D** in 400μM aqueous solution of TC (b,f), **CP@DC**

fabricated in acetonitrile in water (c,g), and **CP@DC** fabricated in acetonitrile in 400μM aqueous solution of TC.

References

(1) Brill, M. H.; Vik, M. Kubelka, Paul. In *Encyclopedia of Color Science and Technology*, Luo, R. Ed.; Springer Berlin Heidelberg, 2014; pp 1–3.
(2) Kubelka, P. a. M., F. *Zeitschrift für Technische Physik* **1931**, *12*, 593–601.
(3) Tauc, J.; Grigorovici, R.; Vancu, A. Optical Properties and Electronic Structure of Amorphous Germanium. *physica status solidi (b)* **1966**, *15* (2), 627–637. DOI: https://doi.org/10.1002/pssb.19660150224.
(4) Gehlen, M. H. The centenary of the Stern-Volmer equation of fluorescence quenching: From the single line plot to the SV quenching map. *Journal of Photochemistry and Photobiology C: Photochemistry Reviews* **2020**, *42*, 100338. DOI: https://doi.org/10.1016/j.jphotochemrev.2019.100338.
(5) Singh, V.; Mondal, P. C.; Kumar, A.; Jeyachandran, Y. L.; Awasthi, S. K.; Gupta, R. D.; Zharnikov, M. Surface confined heteroleptic copper(ii)–polypyridyl complexes for photonuclease activity. *Chem Commun* **2014**, *50* (78), 11484–11487, 10.1039/C4CC05063K. DOI: 10.1039/C4CC05063K.
(6) Allegrini, F.; Olivieri, A. C. IUPAC-Consistent Approach to the Limit of Detection in Partial Least-Squares Calibration. *Anal Chem* **2014**, *86* (15), 7858–7866. DOI: 10.1021/ac501786u.
(7) Kresse, G.; Hafner, J. Ab initio molecular dynamics for liquid metals. *Physical Review B* **1993**, *47* (1), 558–561. DOI: 10.1103/PhysRevB.47.558.
(8) Román-Pérez, G.; Soler, J. M. Efficient Implementation of a van der Waals Density Functional: Application to Double-Wall Carbon Nanotubes. *Phys Rev Lett* **2009**, *103* (9), 096102. DOI: 10.1103/PhysRevLett.103.096102.
(9) Krukau, A. V.; Vydrov, O. A.; Izmaylov, A. F.; Scuseria, G. E. Influence of the exchange screening parameter on the performance of screened hybrid functionals. *The Journal of Chemical Physics* **2006**, *125* (22). DOI: 10.1063/1.2404663 (accessed 4/8/2026).
(10) Blöchl, P. E. Projector augmented-wave method. *Physical Review B* **1994**, *50* (24), 17953–17979. DOI: 10.1103/PhysRevB.50.17953.
